# Boosting Ring Strain and Lewis Acidity of Borirane: Synthesis, Reactivity and Density Functional Theory Studies of an Uncoordinated Arylborirane Fused to *o*‐Carborane

**DOI:** 10.1002/chem.202203265

**Published:** 2022-12-01

**Authors:** Yuxiang Wei, Junyi Wang, Weiguang Yang, Zhenyang Lin, Qing Ye

**Affiliations:** ^1^ Department of Chemistry Southern University of Science and Technology 518055 Shenzhen (P. R. China; ^2^ Department of Chemistry The Hong Kong University of Science and Technology Clear Water Bay Kowloon Hong Kong; ^3^ Institute for Inorganic Chemistry Julius-Maximilians-Universität Würzburg Am Hubland 97074 Würzburg Germany; ^4^ Institute for Sustainable Chemistry& Catalysis with Boron Julius-Maximilians-Universität Würzburg Am Hubland 97074 Würzburg Germany

**Keywords:** borirane, carborane, fused boracycles, Lewis acidity, ring strain

## Abstract

Among the parent borirane, benzoborirene and *ortho*‐dicarbadodecaborane‐fused borirane, the latter possesses the highest ring strain and the highest Lewis acidity according to our density functional theory (DFT) studies. The synthesis of this class of compounds is thus considerably challenging. The existing examples require either a strong π‐donating group or an extra ligand for *B*‐coordination, which nevertheless suppresses or completely turns off the Lewis acidity. The title compound, which possesses both features, not only allows the 1,2‐insertion of P=O, C=O or C≡N to proceed under milder conditions, but also enables the heretofore unknown dearomative 1,4‐insertion of Ar−(C=O)− into a B−C bond. The fusion of strained molecular systems to an *o*‐carborane cage shows great promise for boosting both the ring strain and acidity.

## Graphical abstract

**An uncoordinated** *o*‐carborane‐fused arylborirane was synthesized, which not only allows the 1,2‐insertion of C=O or C≡N to proceed under milder conditions, but also enables the heretofore unknown dearomative 1,4‐insertion of Ar−(C=O)− into a B−C bond. The detailed computational mechanism studies show the relevance of both ring strain and Lewis acidity to the unique reaction profiles.

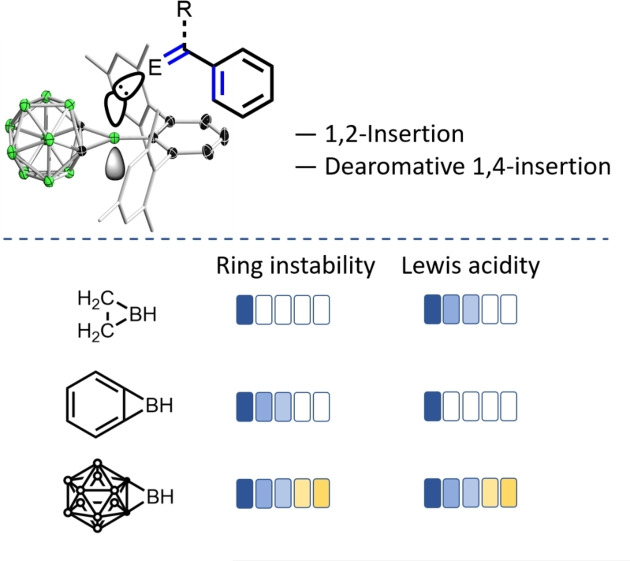

## Introduction

**Figure 1:**
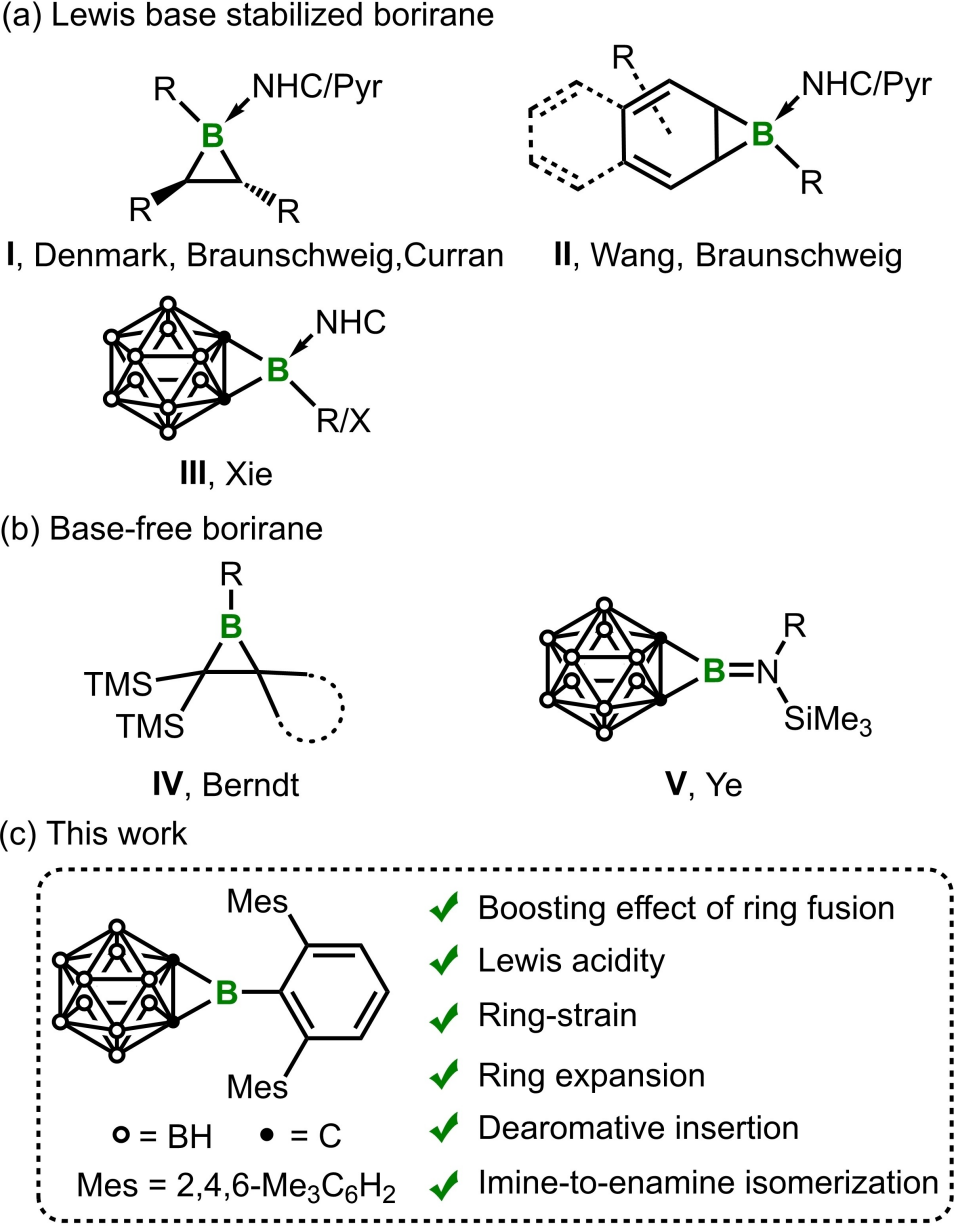
Representative examples of a) coordinated boriranes, b) uncoordinated boriranes and c) this work.

Borirane C_2_BH_5_ features a C_2_B three‐membered ring that is isoelectronic with cyclopropane cation (C_3_H_5_^+^). Unlike the 2π aromatic borirene C_2_BH_3_, another class of highly strained boracycles,^[1]^ boriranes lack the aromatic stabilization. Thus, boriranes are expected to be more Lewis acidic and more reactive than the same *B*‐substituted borirenes. In fact, the majority of the reported boriranes were synthesized as Lewis base adducts, which improves the stability, but also inevitably turns off an important feature of borirane, that is, Lewis acidity. A few different synthetic protocols for the preparation of coordinated boriranes (i. e., **I** monocyclic, **II** bi‐ or polycyclic, **III** carborane‐fused, Figure 1) have been reported, including photoisomerization by the groups of Denmark, Wang and Xie,^[2]^ formal borylene trapping reaction by the Braunschweig group,^[3]^ double hydroboration by the Curran group,^[4]^ and double salt elimination by the Xie group.^[5]^ The pioneering work on the uncoordinated boriranes (**IV**, Figure 1) was done by Berndt and co‐workers in the 1980s–90 s. Yet hardly any new uncoordinated boriranes were reported in the next three decades and the knowledge of their reaction chemistry remained severely limited. Recently, inspired by the unique advantages of carboranes in terms of electronic as well as kinetic stabilization of reactive species,^[6]^ a new class of uncoordinated boriranes (**V**, Figure 1) were developed in our laboratory.^[7]^ Gratifyingly, **V** has found application in inorganic synthesis – the first carboranyl iminoboranes were attained from **V** via a THF‐catalyzed isomerization reaction.^[8]^

On the other hand, in the condition that there are some examples about *o*‐carborane‐fused heterocycle with appealing reactivities,^[9]^ the unique properties of *o*‐carborane, such as the σ delocalized skeletal bonding, prompt us to believe that there is still mystery about this class of compounds to be unraveled. For instance, while the basic structure of a regular borirane consists of three electron‐precise σ bonds, the C_2_B three‐membered ring in **V** consists of two BC σ bonds and one skeletal CC unit. How would this difference affect the ring strain of the carborane‐fused boriranes? In addition, the π‐donating amino group in **V** limits the Lewis acidity. Could we take advantage of the electron‐withdrawing property of the cage‐carbons to construct the boriranes of boosted Lewis acidity by replacing the amino group of **V**?

Inspired by the recent success in applying bulky terphenyl substituent for the isolation and structural characterization of a benzoborirene,^[10]^ we set out to synthesize a three‐dimensional (3D) analogue following the 2D/3D relationship^[11]^ between benzene and carborane. As demonstrated in this work, this 3D analogue represents, in comparison to the conventional boriranes, a rare example with boosted Lewis acidity and boosted ring strain in one.

## Results and Discussion

**Scheme 1:**
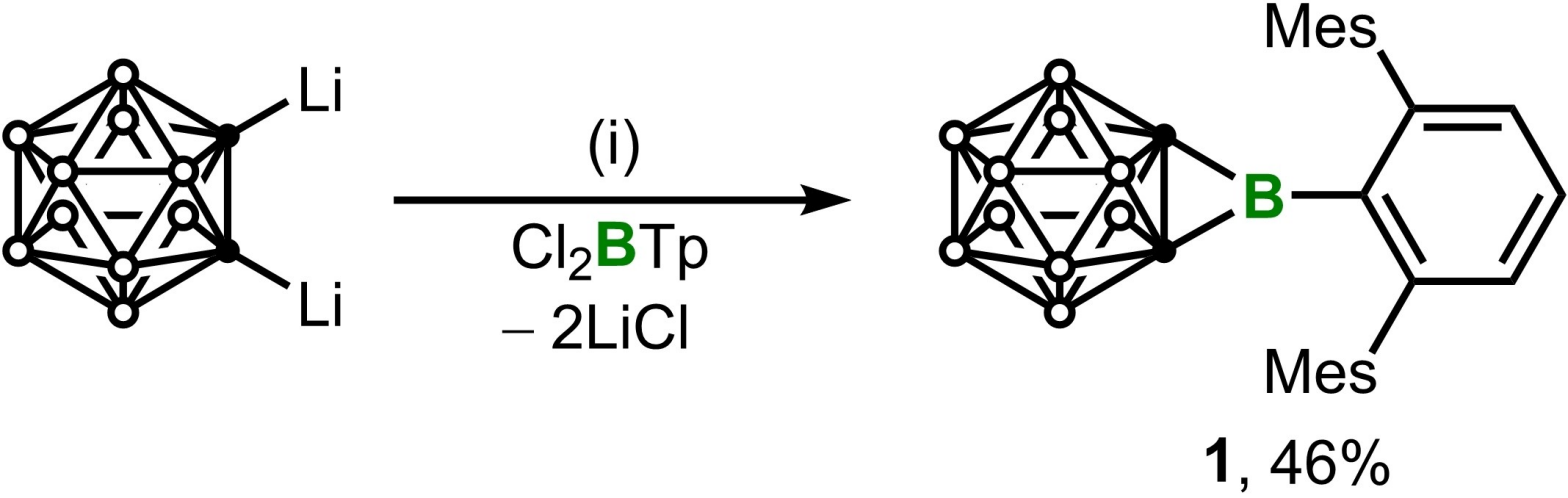
Synthesis of **1**. (i) Toluene, 80 °C, 3 days.

Synthesis of *B*‐aryl carborane‐fused borirane **1** was conducted by treatment of TpBCl_2_ (Tp=2,6‐Mes_2_C_6_H_3_, Mes=2,4,6‐Me_3_C_6_H_2_)^[12]^ with 1.5 equivalents of Li_2_C_2_B_10_H_10_ at 80 °C via salt elimination (Scheme 1). After work‐up, colorless crystals were obtained with isolated yield of 46 %. The ^11^B NMR spectrum of **1** displays a lower‐field signal (δ_B_ 35.9) with respect to the previously reported amino variant (δ_B_ 24.0),^[7]^ which is indicative of less electron density on boron. The ^1^H NMR spectrum shows two sharp singlets at 1.87 and 2.16 ppm with an integration ratio of 12 : 6, which can be assigned to the methyl of mesityl groups, suggesting that the product should be highly symmetric.

**Figure 2:**
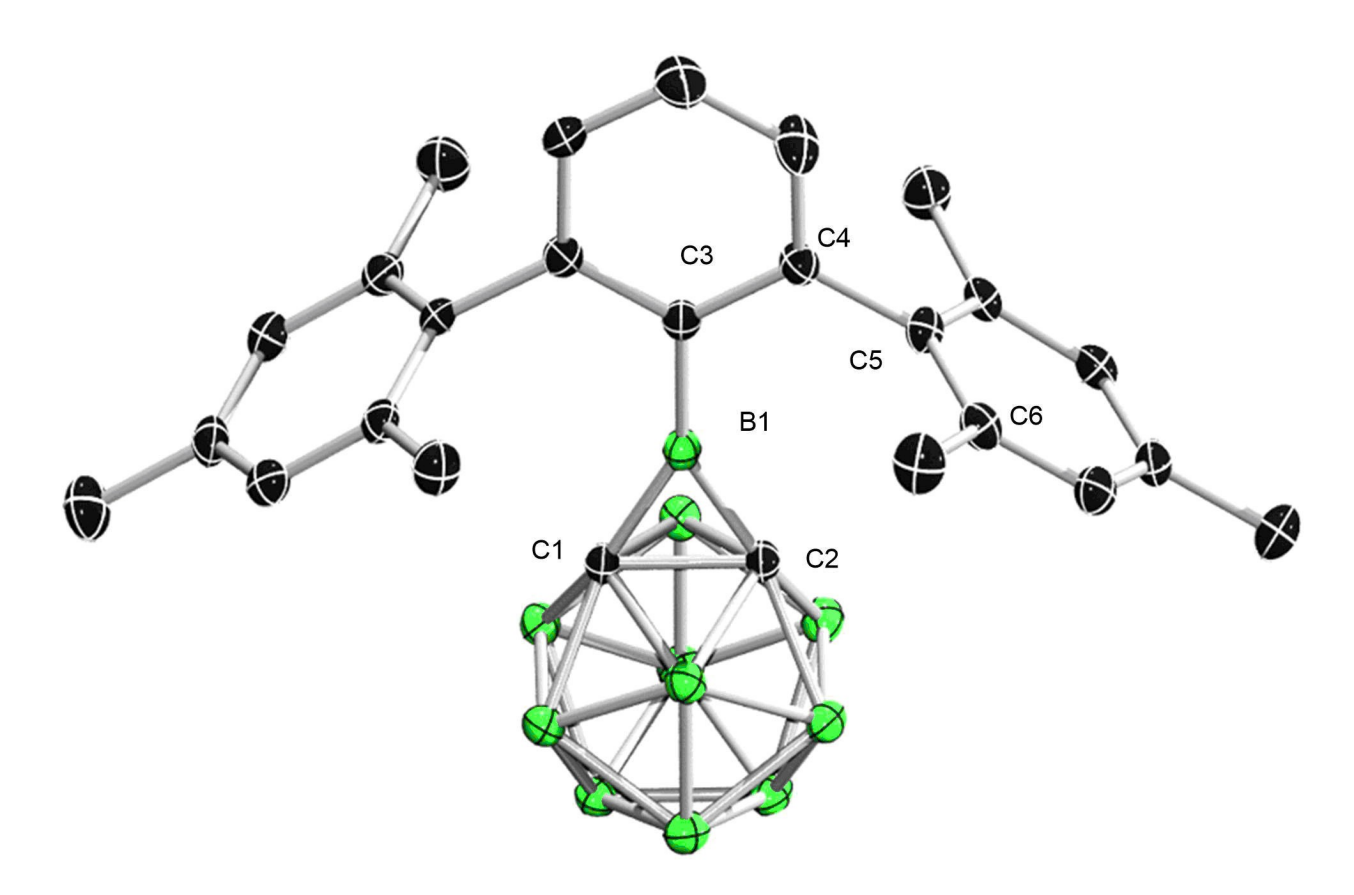
Solid‐state structure of **1**. Hydrogen atoms have been removed for clarity. Thermal ellipsoids are drawn at the 50 % probability level. Selected bond lengths (Å) and angles (deg): For **1**, B1−C1 1.553(2), B1−C2 1.553(2), B1−C3 1.516(3), C1−C2 1.660(2), C1−B1−C3 147.69(6), C2−B1−C3 147.69(6), C1−B1−C2 64.61(12), C4−C3−B1−C2 4.38(12), C6−C5−C4−C3 84.75(16).

Single‐crystal X‐ray diffraction analysis shows that **1** features a desired C_2_B three‐membered ring that is fused to the carborane cluster (Figure 2). The sum of angles around the central boron is 360.0°, indicating its trigonal planar geometry. The boron‐bound phenyl ring is nearly coplanar to the borirane ring (C4−C3−B1−C2 (4.38(12)°), while the flanking mesityl groups are almost perpendicular to the central phenyl ring (C6−C5−C4−C3: 84.75(16)°). Thus, **1** is in an approximate *C_2v_* symmetry in the solid state. The B1−C1, B1−C2 (1.553(2) Å) and C1−C2 (1.660(2) Å) distances are comparable to those (B−C 1.576(4) Å, C−C 1.640(3) Å) in the *B*‐amino variant. Most remarkably, the exocyclic B1−C3 distance of 1.516(3) Å is essentially shorter than the common B(*sp* ^*2*^)−Ar single bonds (e. g., Ar−B(CO)_2_ 1.571(3) Å),^[13]^ and even falls into the range of a the B=C double bond (1.44–1.52 Å),^[14]^ thus indicating the presence of considerable B−Ar p‐π interaction in the solid state. This interaction reveals the enhanced acidity of boron center substituted by electron‐withdrawing *o*‐carborane. The principal interacting orbital analysis^[15]^ supports the claim of considerable B−Ar p‐π interaction (Figure S32 in Supporting Information). The second PIO pair, which represents the B−Ar p‐π interaction, contributes 22.1 % to the overall B−Ar bonding interaction. Besides, the nucleus‐independent chemical shift (NICS) calculations were conducted to evaluate the aromatic character at the center of the Tp aryl ring that is directly bonded to the Lewis acidic boron.^[16]^ Indeed, the NICS value clearly shows a decrease in its aromaticity, a result of the B−Ar p‐π interaction, with respect to TpH (NICS(0): −5.92 for **1** vs. −7.68 for TpH; NICS(1): −8.25 for **1** vs. −9.29 for TpH. see Table S8).

**Figure 3:**
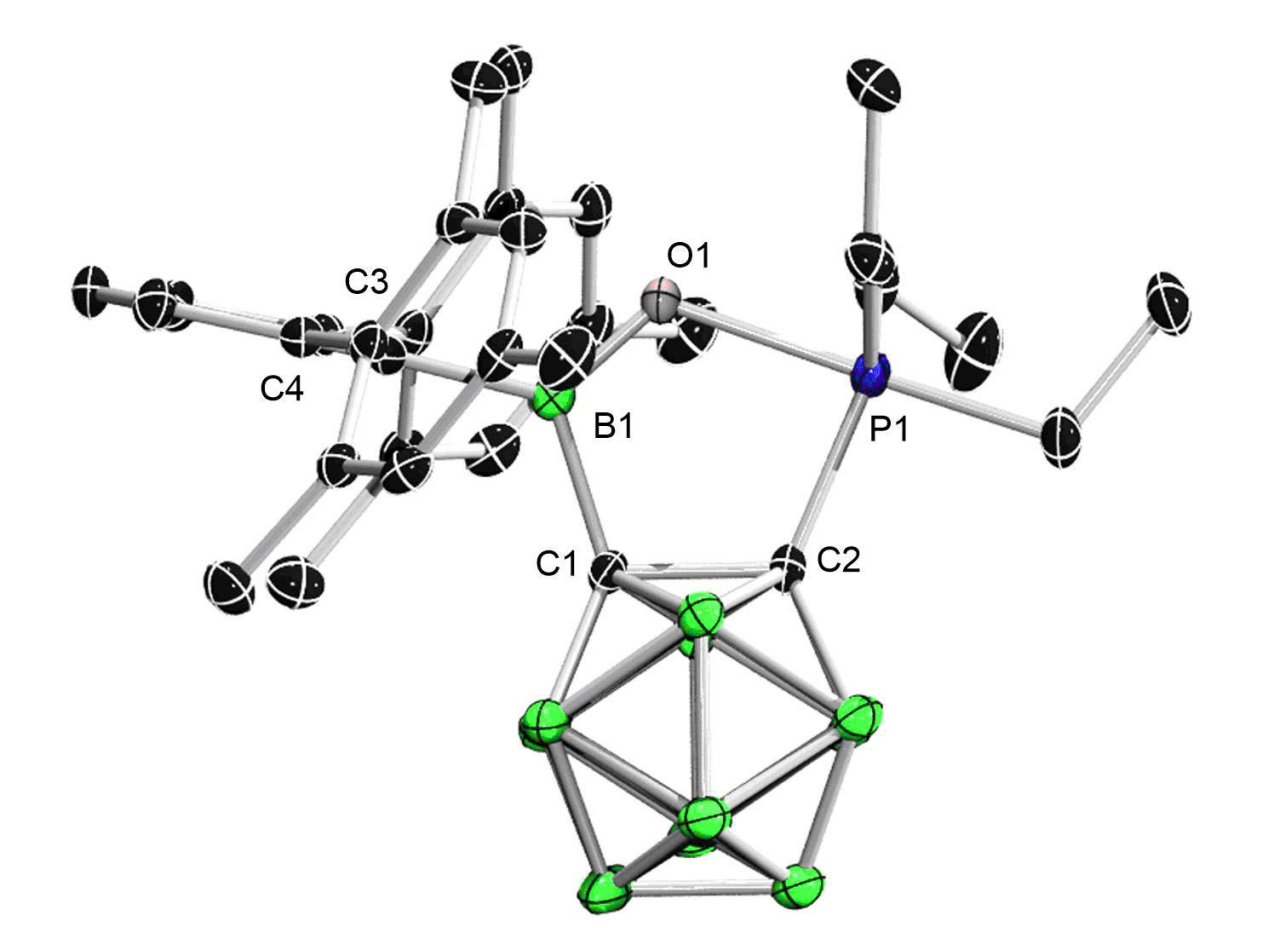
Solid‐state structure of **2**. Hydrogen atoms have been removed for clarity. Thermal ellipsoids are drawn at the 50 % probability level. Selected bond lengths (Å) and angles (deg): B1−C1 1.616(2), C1−C2 1.6664(19), P1−C2 1.8683(15), O1−P1 2.0532(10), B1−O1 1.3096(18), B1−C3 1.594(2), C1−B1−C3 122.09(12), C1−B1−O1 111.80(12), C3−B1−O1 126.04(13).

**Scheme 2:**
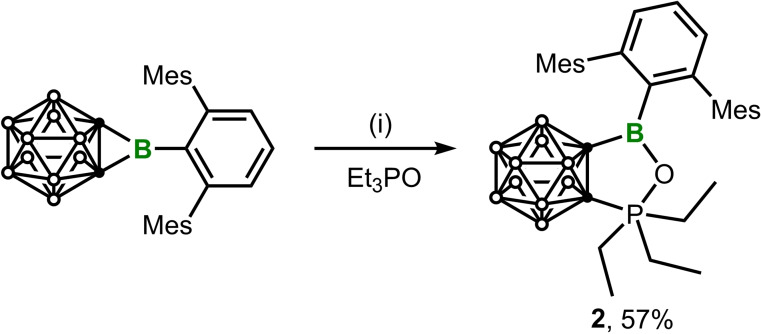
Ring‐expansion reactions of **1** with Et_3_PO. (i) Toluene, r. t., 2 h.

The Gutmann–Beckett method^[17]^ was applied for the experimental assessment of the Lewis acidity of **1**. To this end, an equimolar amount of Et_3_PO was added to a toluene solution of **1**. The ^11^B NMR spectrum showed a new signal at 35.5 ppm, while the ^31^P NMR spectrum displayed a singlet at–17.64 ppm that is significantly high‐field shifted compared to that of Et_3_PO (44.77 ppm), thus implying the formation of an unexpected product rather than the **1**⋅OPEt_3_ adduct. After workup, the product was isolated as a crystalline solid in 57 % yield. The single‐crystal structure is depicted in Figure 3, which revealed the P=O insertion into one of the BC bonds, affording the ring expansion product **2** (Scheme 2). The central boron remains trigonal planar as indicated by the sum (359.9°) of the angles around boron. The C_2_BOP five‐membered heterocycle is nearly planar as indicated by the sum (539.9°) of internal angles. In addition, the exocyclic B1−C3 bond of 1.594(2) Å is elongated by 5 % compared to that (1.516(3) Å) in **1**, which can be explained by the lack of B−Ar p‐π interaction due to the nearly perpendicular orientation between the Ar and the boron‐centered trigonal plane (C1−B1−C3−C4 82.99(18)°). The unusual reactivity with Et_3_PO is perhaps attributable to the pronounced ring strain in **1** that is imposed by the fusion to a carborane cage.

**Scheme 3:**
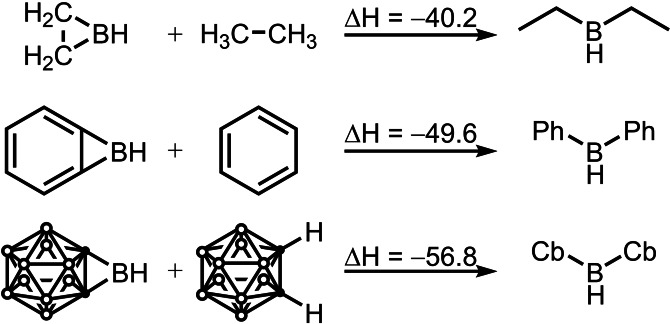
Strain energies calculated for the parent borirane, benzoborirene and carborane‐fused borirane based on the designed isodesmotic equations. The relative energies are given in kcal/mol.

To provide additional support to our claim that carborane‐fused boriranes are expected to show pronounced ring strain, we calculated and compared the ring‐opening reaction enthalpies for the parent borirane, benzoborirene, and carborane‐fused borirane based on the designed isodesmotic reactions shown in Scheme 3. The results show that the strain energy of carborane‐fused borirane is considerably higher than that of borirane, a non‐fused borirane. Interestingly, the reaction enthalpies of benzoborirene and carborane‐fused borirane, which are expected to be comparable, also differ significantly by 7.2 kcal/mol. This unexpected result can be conveniently related to the fact that π delocalization to the boron center is significant in benzoborirene, but not in carborane‐fused borirane.

Besides, we evaluated the Lewis acidity of the three parent species discussed above by calculating their fluoride ion affinity (FIA) and hydride ion affinity (HIA) energies (Tables S9 and S10) following a literature protocol.[^15a^, ^18^] For the purpose of comparison, we also calculated FIA and HIA for the experimentally characterized carborane‐fused borirane **1** and the *B*‐amino analogue **V**.^[7]^ The calculation results indicate that the parent carborane‐fused borirane has the highest FIA and HIA energies, and therefore possesses the highest acidity, a finding that can be attributed to both the highly electron‐withdrawing *o*‐carborane substituent and lack of π delocalization. More remarkably, the parent carborane‐fused borirane shows even larger FIA and HIA than SbF_5_ and B(C_6_F_5_)_3_, respectively (Tables S9 and S10). The parent benzoborirene, due to the π delocalization, is found to be less acidic than the parent borirane. As expected, a notable increase in acidity was also found in **1** when compared with the *B*‐amino analogue **V**,^[7]^ as a result of the considerable N−B π interaction in the latter. All of these calculations clearly indicate a boosted ring strain and increased Lewis acidity in the experimentally characterized carborane‐fused borirane **1**, thus leading us to expect a significantly enhanced reactivity of **1**.

**Scheme 4:**
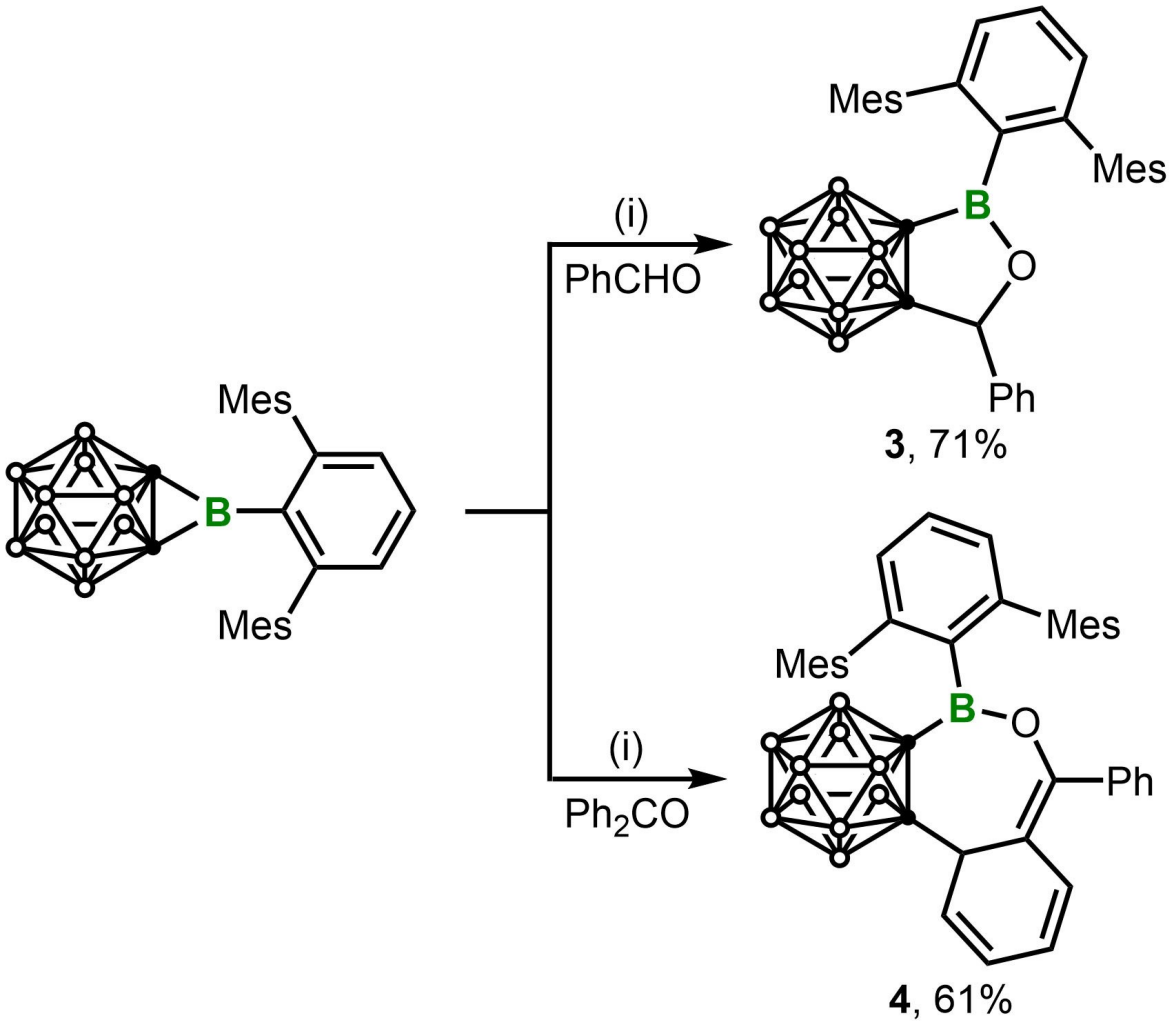
Ring‐expansion reactions of **1** with PhCHO and Ph_2_CO. (i) Toluene, r. t., 2 h.

**Figure 4:**
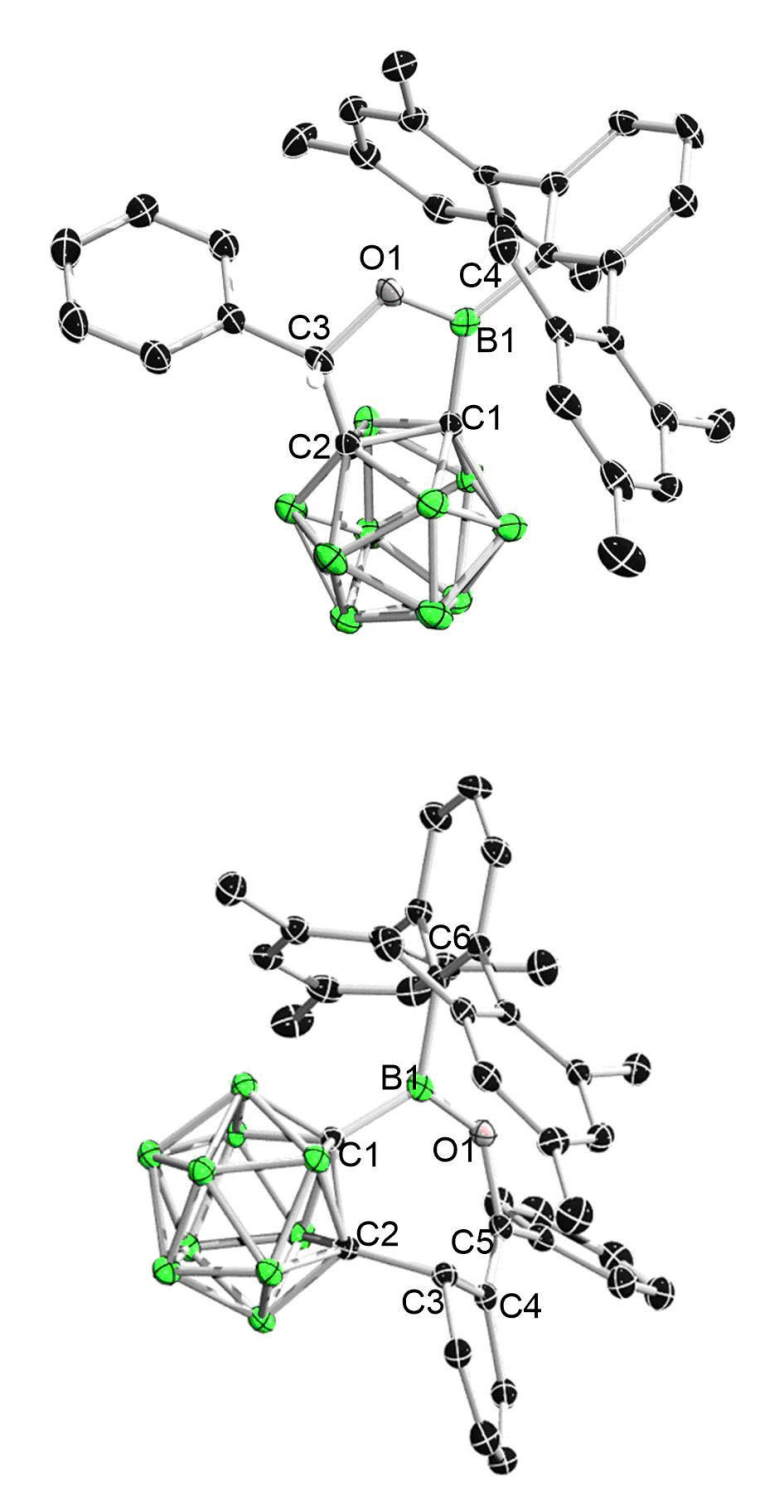
Solid‐state structure of **3** (upper) and **4** (bottom). Hydrogen atoms have been removed for clarity. Thermal ellipsoids are drawn at the 50 % probability level. Selected bond lengths (Å) and angles (deg): For **3**, C1−B1 1.587(5), B1−O1 1.365(4), O1−C3 1.453(4), C3−C2 1.520(5), C1−C2 1.653(4), B1−C4 1.575(5), C1−B1−C4 131.3(3), O1−B1−C1 109.5(3), O1−B1−C4 119.2(3); For **4**, B1−C1 1.608(2), C1−C2 1.6646(19), C2−C3 1.5551(19), C3−C4 1.5151(19), C4−C5 1.344(2), C5−O1 1.3979(17), B1−O1 1.3573(19), B1−C6 1.597(2), C1−B1−C6 124.02(13), C1−B1−O1 120.65(13), O1−B1−C6 115.28(12).

To probe the versatility of the ring expansion reaction, the polar and unsaturated organic substrates such as benzaldehyde, benzophenone, benzonitrile and acetonitrile were reacted with **1**. The reaction with benzaldehyde (PhCHO) in toluene at room temperature afforded **3** in 71 % yield (Scheme 4). Compound **3** displays a singlet at δ_H_ 5.29 for C*H*O, and a resonance at δ_B_ 46.2 for the 3‐coordinate boron. Single‐crystal X‐ray diffraction analysis shows a planar five‐membered ring with a short B1−O1 (1.365(4) Å) single bond(Figure 4).^[14]^ It is worth noting that this reaction is accomplished at room temperature within minutes, while a similar reaction of a NHC‐coordinated *o‐*carborane‐fused borirane reported by the Xie group^[5a]^ requires 80 °C. This difference should be a result of the combination of ring strain and Lewis acidity achieved in **1**. In fact, the unique features of **1** not only allow reactions to proceed under milder conditions, but also lead to a unique reaction profile. The reaction of **1** with an equimolar amount of benzophenone (Ph_2_CO) in toluene afforded a white powder **4**. The ^11^B NMR spectrum displayed a new resonance at δ_B_ 43.3, which is comparable with that of **3**. However, it is noted that the ^1^H spectrum showed 6 different singlets from 1.70‐2.37 for the six methyl groups of Tp, indicating a more crowded environment in the product. Most remarkably, it displays a new set of signals at 2.65 (d, 1H, *sp* ^*3*^‐CH), 5.35 (dd, 1H, *sp* ^*2*^‐CH), 5.50 (dd, 1H, *sp* ^*2*^‐CH), 5.73 (dd, 1H, *sp* ^*2*^‐CH) and 5.84 (d, 1H, *sp* ^*2*^‐CH), which completely fall out of the aromatic region. After workup, the product was isolated as a white solid in 61 % yield. Single‐crystal

X‐ray diffraction analysis indicated that the benzophenone is inserted in a [4+3] manner with one of the phenyl groups being dearomatized, thus giving a C_5_OB seven‐membered ring (Figure 4). The C5−O1 bond (1.3979(17) Å) in **4** is notably shorter than the C3−O1 bond (1.453(4)) in **3**, which could be explained by the higher *s*‐character of C5 (*sp* ^*2*^) in **5** than that of C3 (*sp* ^*3*^) in **3** for C−O bonding. In addition, the distances between C3−C4 and C4−C5 are 1.5151(19) Å and 1.344(2) Å respectively, which are in the range for typical C−C single and C=C double bonds, further confirming the Lewis structure of **4** as depicted in Scheme 4. Indeed, this unique reactivity pattern represents a striking example of dearomative 1,4‐insertion of Ar−(C=O)− into a C−B bond, especially when considering that C−B has a higher average σ‐bond enthalpy than C−C bond (*D_0_* (kJ/mol) C−B 365 vs. C−C 358). The relevance of the boosted Lewis acidity and ring strain is further reflected by the fact that no reaction was observed between the benzoborirene [C_6_H_4_{μ‐BTrip}]^[10]^ (Trip=2,4,6‐^*i*^Pr_3_C_6_H_2_), a 2D analogous compound of **1** that is less Lewis acidic and less strained, and benzophenone even at 60 °C for hours.

**Figure 5:**
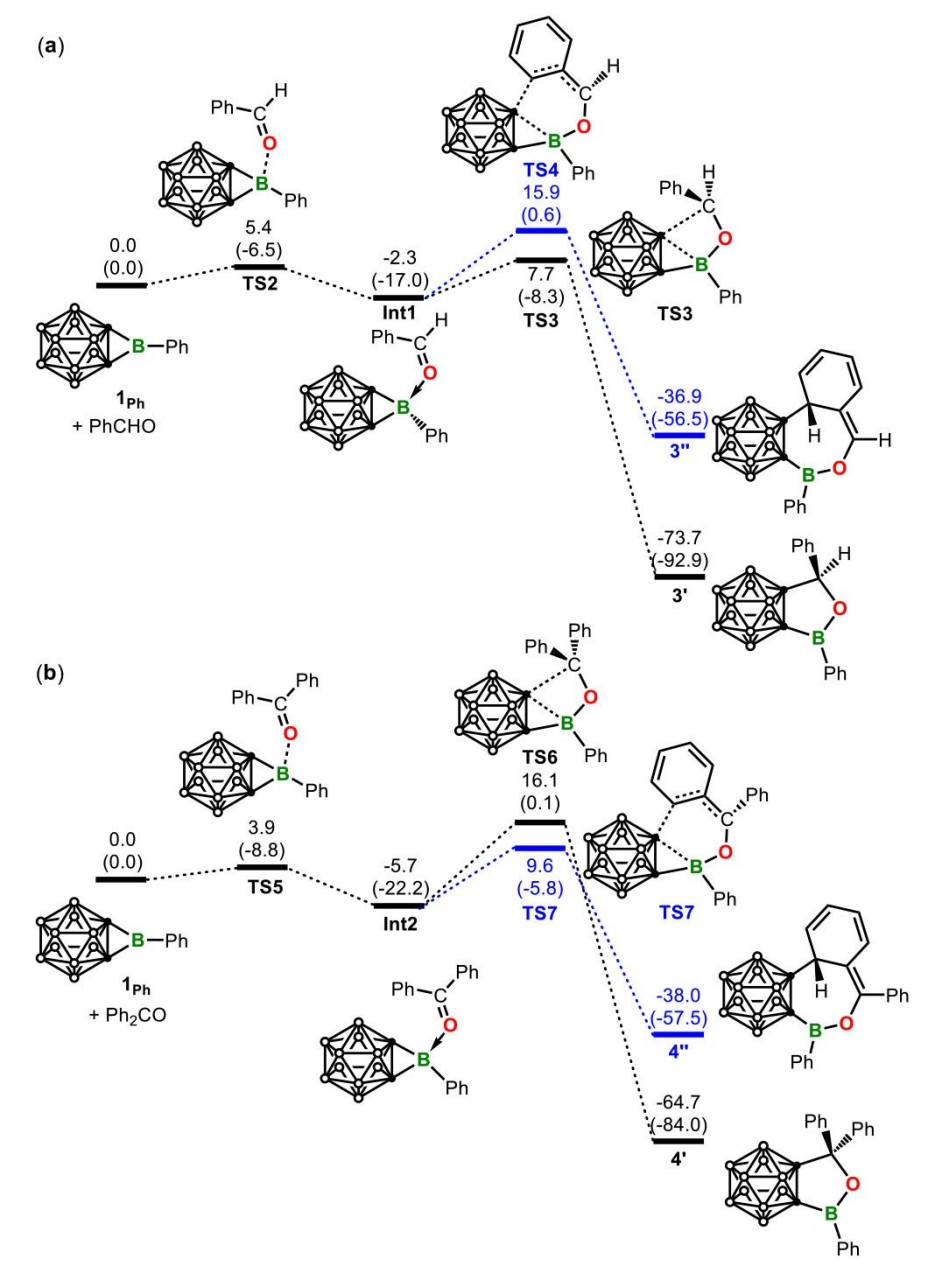
Energy profiles calculated for the ring expansion reaction of **1_Ph_** with PhCHO (**a**) and Ph_2_CO (**b**). The relative free energies (calculated at 298 K) and electronic energies (in parentheses) are given in kcal/mol.

To further investigate the reason why the reactions of borirane **1** with PhCHO and Ph_2_CO render distinctive products **3** and **4**, mechanisms of the above reactions were analyzed by DFT calculations using the *B*‐phenyl carborane‐fused borirane **1_Ph_** as the model (Figure 5). We found that coordination of carbonyl oxygen to the tricoordinate boron center is the first event for both reactions, which activates the carbonyl C=O double bond, weakens the two B−C bonds within the borirane 3‐membered ring, and facilitates the followed migratory insertion involving cleavage of a B−C bond. In the reaction with PhCHO, carbonyl oxygen coordination followed by insertion of carbonyl moiety into one of the two B−C σ bonds occurs via a [2σ+2π] addition to give the product **3’** (a model for **3**) with a barrier of 10.0 kcal/mol. In the reaction with Ph_2_CO, steric hindrance, as a result of an additional phenyl group when compared to PhCHO, makes the [2σ+2π] addition less favorable with a barrier of 21.8 kcal/mol. Instead, a [2σ+4π] addition, which involves one C=C unit from the relevant phenyl group, to give the product **4’** (a model for **4**) becomes more favorable with a barrier of 15.3 kcal/mol.

One fine point to note is that in literature an NHC‐coordinated borirane was reported to react with PhCHO to give a product similar to **3**. However, the reaction occurs only upon heating,^[5a]^ in contrast to the reaction of **1** under ambient temperature. Because of the additional coordination, the barrier for the carbonyl moiety insertion was calculated to be 21.3 kcal/mol (Figure S33), 11.3 kcal/mol higher than the insertion barrier calculated for the reaction of the tricoordinated borirane **1_Ph_**, consistent with the experimental observation that thermal condition is needed.

**Figure 6:**
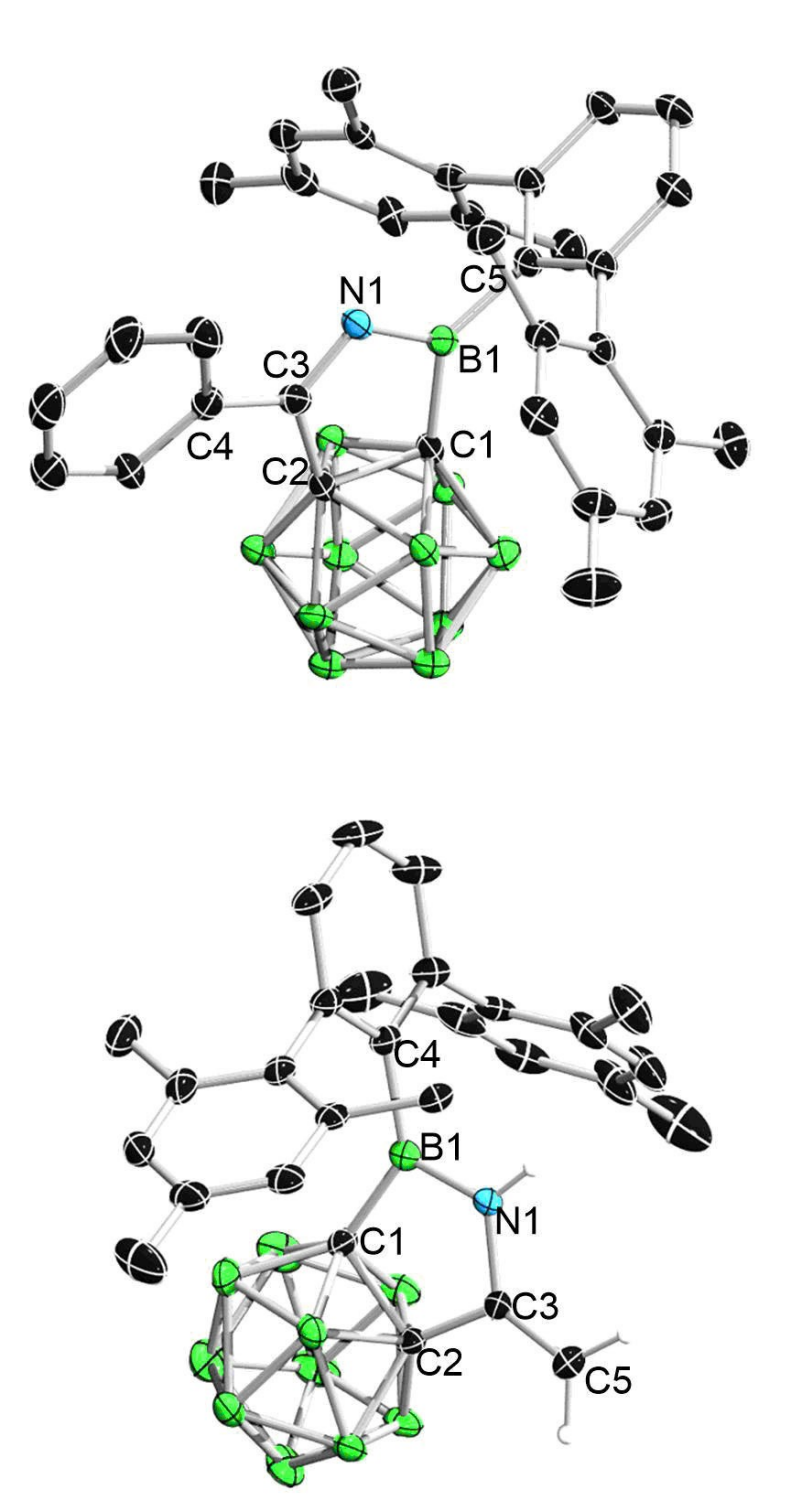
Solid‐state structure of **5** (upper) and **6** (bottom). Hydrogen atoms have been removed for clarity. Thermal ellipsoids are drawn at the 50 % probability level. Selected bond lengths (Å) and angles (deg): For **5**, B1−C1 1.599(3), C1−C2 1.657(2), N1−B1 1.463(3), N1−C3 1.297(2), C3−C2 1.535(3), C3−C4 1.476(3), B1−C5 1.571(3), C1−B1−N1 108.76(15), C1−B1‐C5 128.85(16), N1−B1−C5 122.38(16); For **6**, C1−B1 1.712(3), B1−N1 1.399(2), N1−C3 1.411(2), C3−C2 1.491(2), C3−C5 1.322(2), C1−C2 1.650(2), B1−C4 1.572(2), C1−B1−C4 130.32(14), N1−B1−C1 105.43(13), N1−B1−C4 124.23(14).

**Scheme 5:**
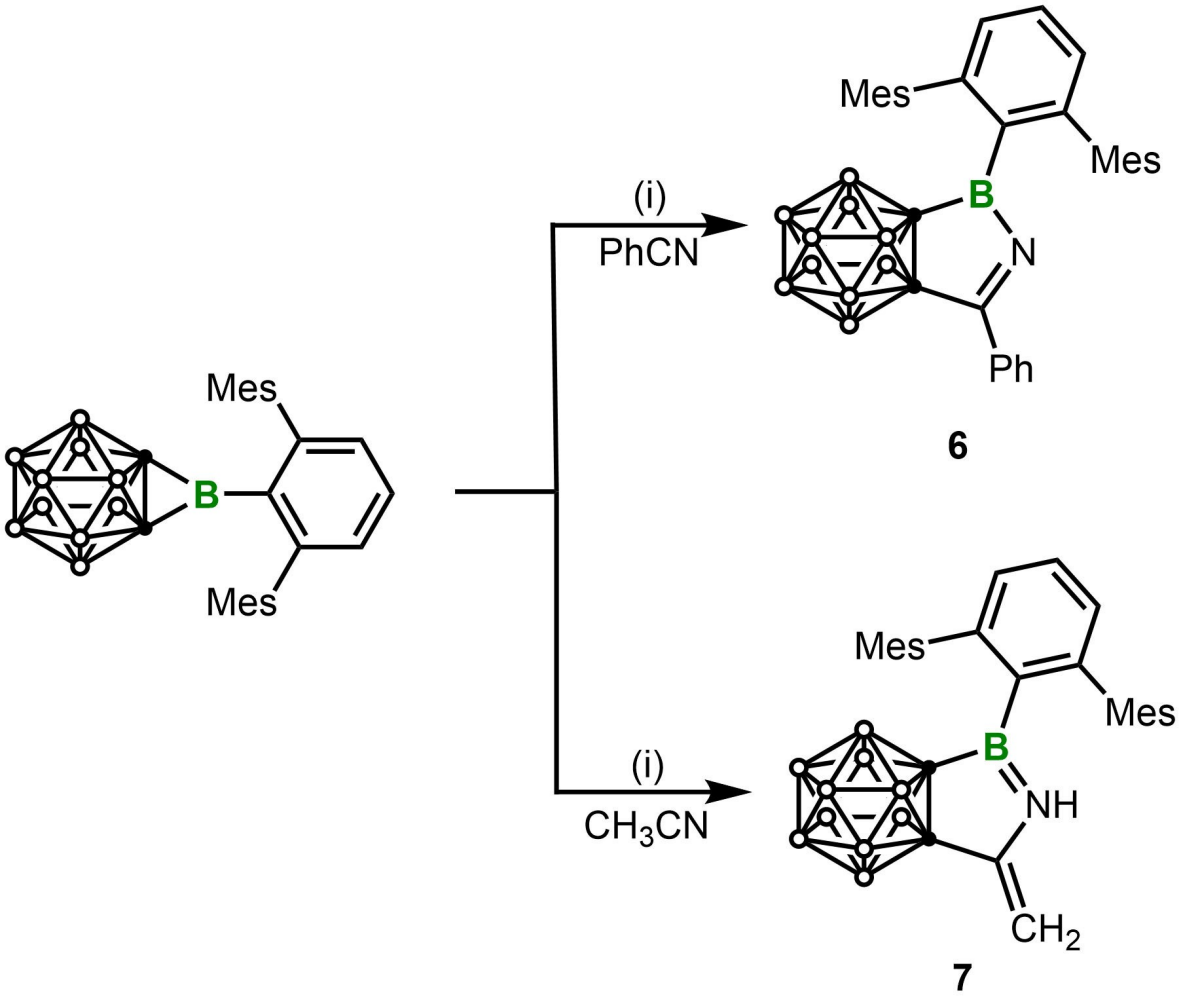
Ring‐expansion reactions of **1** with PhCN and CH_3_CN. (i) Toluene, r. t., 2 h.

Finally, we studied the reaction between **1** and nitrile with (i. e., acetonitrile) or without (i. e., benzonitrile) α‐H. The reaction with an equimolar amount of benzonitrile (PhCN) in toluene at room temperature afforded the CN insertion product **5** in 62 % yield. The atom connectivity of **5** was confirmed by single‐crystal X‐Ray diffraction analysis. The N1−C3 bond length of 1.297(2) Å is typical for a N=C double bond. It should be noted that the same reaction with Xie's NHC‐coordinated borirane requires 80 °C.^[5a]^ Furthermore, the reaction with an equimolar amount of acetonitrile led to the formation of a new 3‐coordinate boron‐containing species showing a resonance at δ_B_ 42.1. Instead of displaying a singlet for the methyl group of the expected insertion product, ^1^H NMR spectrum displayed two doublets at 3.67 ppm and 4.06 ppm, and a singlet at 5.85 ppm, with an integration ratio of 1 : 1 : 1. Thus, the reaction should differ from that with benzonitrile. After workup, the product was isolated as white solid in 62 % yield. Single‐crystal X‐ray diffraction analysis revealed the atom connectivity that is not different form the expected product upon the first glance (Figure 6). However, the B1−N1 of 1.399(2) Å is significantly shorter than that (1.463(3) Å) in in **5**, while the exocyclic C3−C5 of 1.322(2) Å falls in the range of C=C double bonds. Thus combined, **6** (Scheme 5) should be formed via CN insertion followed by an imine‐to‐enamine isomerization. And there is no equilibrium between imine and enamine in solution as indicated by the NMR spectra of **6**.

## Conclusion

In summary, the DFT calculation results predict that borirane should possess higher ring strain and Lewis acidity when fused to an *o*‐carborane cage through the skeletal CC unit. The successful synthesis of the title compound and the reactivity investigation have brought this prediction to fruition. In the reactions with triethylphosphine oxide, benzaldehyde, and benzonitrile, the P=O, C=O or C≡N bonds were 1,2‐inserted into one of the B−C bonds, affording products **2**, **3** and **5**, respectively. In contrast, benzophenone (Ph_2_CO) was inserted in a rare [4+2] manner with one of the phenyl groups being dearomatized. Treatment of **1** with acetonitrile gave an enamine **6** via CN insertion followed by an imine‐to‐enamine irreversible isomerization. The detailed computational mechanism studies show the relevance of both ring strain and Lewis acidity to the unique reaction profiles. Clearly, fusion of strained molecular systems to an *o*‐carborane cage deserves more attention.

## Experimental Section

**Synthesis of 1**: TpBCl_2_ (790 mg, 2 mmol) was slowly added to a toluene (30 ml) solution of *ortho*‐carborane dilithium salt (462 mg, 3 mmol) and stirred two days at 80 °C. The suspension was then filtrated and all the volatiles were removed under reduced pressure to obtain a colorless‐yellow solid. The solid was recrystallized in toluene at−30 °C in the refrigerator. The crystal was filtrated and dried under vacuum to obtain the colorless crystalline solid product (427 mg, 0.91 mmol, 45.6 %).^**11**^ **B NMR** (C_6_D_6_): δ [ppm]=37.93 (s, *B*Tp), −15.12 (d, J=160.3 Hz, *B*_carborane_), −6.73 (d, J=162.6 Hz, *B*_carborane_), 2.62 (t, J=157.3 Hz, *B*_carborane_).

**Synthesis of 2**: Et_3_PO (22 mg, 0.16 mmol) was added to the toluene solution (20 ml) of **1** (76.7 mg, 0.16 mmol) and stirred at room temperature overnight. All the volatiles were removed under reduced pressure to obtain a white solid. A colorless crystalline solid (56 mg, 0.09 mmol, 56.7 %) was obtained by recrystallizing in toluene at −30 °C. ^**11**^ **B NMR** (C_6_D_6_): δ [ppm]=35.53 (s, *B*Tp), −1.36 (t, J=121.5 Hz, *B*_carborane_), −6.67 (d, J=135.7 Hz, *B*_carborane_), −12.04 (m *B*_carborane_).

**Synthesis of 3**: PhCHO (22 mg, 0.21 mmol) was added to the toluene solution (40 ml) of **1** (100 mg, 0.21 mmol) and stirred at room temperature for 2 h. All the volatiles were then removed under vacuum to obtain a white solid. A colorless crystalline solid (86 mg, 0.15 mmol, 71.0 %) was obtained by recrystallizing in toluene at −30 °C. ^**11**^ **B NMR** (C_6_D_6_): δ [ppm]=45.75 (s, *B*Tp), −1.71 (s, *B*_carborane_), −6.10 (s, *B*_carborane_), −8.35 (s, *B*_carborane_), −12.14 to −12.91 (m, *B*_carborane_).

**Synthesis of 4**: Ph_2_CO (38.9 mg, 0.21 mmol) was added to the toluene solution (25 ml) of **1** (100 mg, 0.21 mmol) and stirred at room temperature overnight. All the volatiles were removed under reduced pressure to obtain a white solid. A colorless crystalline solid (84 mg, 0.13 mmol, 60.5 %) was obtained by recrystallizing in toluene at −30 °C. ^**11**^ **B NMR** (C_6_D_6_): δ [ppm]=43.32 (s, C*B*O), 2.76 (m, *B*_carborane_), −4.11 (m, *B*_carborane_), −9.73 (m, *B*_carborane_).

**Synthesis of 5**: PhCN (21.6 mg, 0.21 mmol) was added to the toluene solution (25 ml) of **1** (100 mg, 0.21 mmol) and stirred at room temperature for 2 h. All the volatiles were then removed under vacuum to obtain a white solid. A light yellow crystalline solid (75 mg, 0.13 mmol, 62.1 %) was obtained by recrystallizing in toluene at −30 °C. ^**11**^ **B NMR** (C_6_D_6_): δ [ppm]=62.60 (s, C*B*N), −2.29 (s, *B*_carborane_), −5.06 (s, *B*_carborane_), −8.48 (s, *B*_carborane_), −12.86 (s, *B*_carborane_), −14.11 (s, *B*_carborane_).

**Synthesis of 6**: Acetonitrile (4.4 mg, 0.107 mmol) was added to the toluene solution (25 ml) of **1** (50 mg, 0.107 mmol) and stirred at room temperature overnight. All the volatiles were removed under reduced pressure to obtain a white solid. A colorless crystalline solid (39 mg, 0.077 mmol, 71.6 %) was obtained by recrystallizing in toluene at −30 °C. ^**11**^ **B NMR** (C_6_D_6_): δ [ppm]=42.11 (s, *B*Tp), −4.19 (s, *B*_carborane_), −5.38 (s, *B*_carborane_), −6.73 (s, *B*_carborane_), −7.89 (s, *B*_carborane_), −9.70 (s, *B*_carborane_), −12.29 (s, *B*_carborane_).

## Conflict of interest

The authors declare no conflict of interest.

1

## Supporting information

As a service to our authors and readers, this journal provides supporting information supplied by the authors. Such materials are peer reviewed and may be re‐organized for online delivery, but are not copy‐edited or typeset. Technical support issues arising from supporting information (other than missing files) should be addressed to the authors.

Supporting Information

## Data Availability

The data that support the findings of this study are available in the supplementary material of this article.
