# Supplementary material for: Boosting Ring Strain and Lewis Acidity of Borirane: Synthesis, Reactivity and Density Functional Theory Studies of an Uncoordinated Arylborirane Fused to o‐Carborane
Source: Chemistry. 2022 Dec 1;29(5):e202203265. doi: 10.1002/chem.202203265 (PMC10107145; doi:10.1002/chem.202203265)
Supplement: Supplementary file 1 — Supporting Information [file CHEM-29-0-s001.pdf]

Chemistry–A European Journal

Supporting Information

Boosting Ring Strain and Lewis Acidity of Borirane: Synthesis, Reactivity and Density Functional Theory Studies of an Uncoordinated Arylborirane Fused to *o*-Carborane

Yuxiang Wei, Junyi Wang, Weiguang Yang, Zhenyang Lin,* and Qing Ye*

Table of Contents

Experimental procedures S1
NMR spectra S4
Crystal structures S20
Computational details S27
References S48
Author contributions S48

Experimental procedures

General considerations. All manipulations were conducted either under an atmosphere of dry argon or in *vacuo* using standard Schlenk line or glovebox techniques. Solvents were purified by distillation from Na/K alloy under dry argon immediately prior to use. C_6D_6 was degassed by three freeze-pump-thaw cycles and stored over molecular sieves. NMR spectra were acquired on a Bruker *Avance 400* (^{1}H: 400.1 MHz, ^{11}B: 128.4 MHz, ^{13}C{^{1}H}: 100.6 MHz, ^{31}P: 162.0 MHz) NMR spectrometer at 298 K. ^{1}H, ^{13}C{^{1}H} and ^{1}H{^{11}B} NMR spectra were referenced to external TMS. ^{11}B and ^{11}B{^{1}H} NMR spectra were referenced to external $BF_3{\cdot}OEt_2$. ^{31}P NMR spectra were referenced to external H_3PO_4. High resolution mass spectrometry (HRMS) was performed with a Thermo Fisher Scientific Q-Exactive MS System. Elemental analysis (C, H, N) was performed on a vario micro cube CHNS analyzer. $TpBCl_2$[1] and $(C_2B_{10}H_{10})_2Li_2$[2] were synthesized according to the literature.

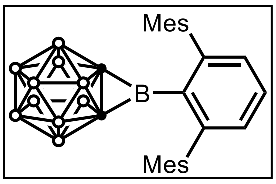

Data of 1: **^{1}H NMR** (C_6D_6): δ [ppm] = 1.87 (s, 12H, Mes ortho-C*H3*), 2.16 (s, 6H, Mes para-C*H3*), 2.1 to 3.8 (br m, 10H, B*H*), 6.80 (s, 4H, Mes meta-Ph*H*), 6.83 (d, 2H, J = 7.63 Hz, meta-Ph*H*), 7.19 (t, 1H, J = 7.67 Hz, para-Ph*H*). **^{1}H{^{11}B} NMR** (C_6D_6): δ [ppm] = 1.65 (s, 2H, B*H*), 1.87 (s, 12H, Mes ortho-C*H3*), 2.16 (s, 6H, Mes para-C*H3*), 2.24 (s, 4H, B*H*), 3.15 (s, 4H, B*H*), 6.80 (s, 4H, Mes meta-Ph*H*), 6.83 (d, 2H, J = 7.63 Hz, meta-Ph*H*), 7.19 (t, 1H, J = 7.67 Hz, para-Ph*H*). **^{11}B NMR** (C_6D_6): δ [ppm] = 37.93 (s, *B*Tp), –15.12 (d, J = 160.3 Hz, $B_{carborane}$), –6.73 (d, J = 162.6 Hz, $B_{carborane}$), 2.62 (t, J = 157.3 Hz, $B_{carborane}$). **^{11}B{^{1}H} NMR** (C_6D_6): δ [ppm] = 35.81 (s, *B*Tp), –15.07 (s, $B_{carborane}$), –6.72 (s, $B_{carborane}$), 1.93 (s, $B_{carborane}$), 3.22 (s, $B_{carborane}$). **^{13}C{^{1}H} NMR** (C_6D_6): δ [ppm] = 20.56 (s, ortho-*C*CH_3 of Mes), 21.04 (s, para-*C*CH_3 of Mes), 128.43 (s, meta-*C*H of Mes), 128.70 (s, para-*C*H of Ph), 134.98 (s, *C*Ph of Mes), 137.34 (s, ortho-*C*CH_3 of Mes), 137.40 (s, meta-*C*H of Ph), 137.90 (s, para-*C*CH_3 of Mes), 155.56 (s, *C*Mes of Ph). **Elemental analysis**: calcd. for $C_{26}H_{35}B_{11}$, C, 66.95; H, 7.560; found C, 66.09; H, 7.51.

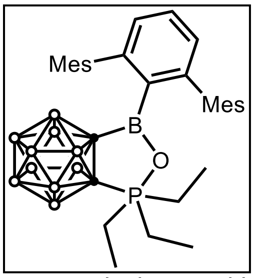

Data of 2: **^{1}H NMR** (C_6D_6): δ [ppm] = 0.68 (m, 9H, CH_2C*H3*), 1.43 (m, 6H, C*H2*CH_3), 0 to 3.8 (br m, 10H, B*H*), 2.19 (s, 6H, Mes ortho-C*H3*), 2.26 (s, 6H, Mes para-C*H3*), 2.30 (s, 6H, Mes ortho-C*H3*), 6.79 (s, 2H,Mes Ph*H*), 6.92, (s, 2H, Mes Ph*H*), 6.98 (d, 2H, J = 7.57Hz, Ph*H*), 7.24 (t, 1H, J = 7.60 Hz, Ph*H*). **^{1}H{^{11}B} NMR** (C_6D_6): δ [ppm] = 0.71 (m, 9H, CH_2C*H3*), 1.44 (m, 6H, C*H2*CH_3), 1.61 (s, 2H, B*H*), 2.21 (s, 6H, Mes para-C*H3*), 2.19 (s, 6H, Mes ortho-C*H3*), 2.26 (s, 6H, Mes para-C*H3*), 2.30 (s, 6H, Mes para-C*H3*), 2.14-2.36 (m, 4H, B*H*), 2.4 (s, 2H, B*H*), 2.93 (s, 1H, B*H*), 3.05 (s, 1H, B*H*), 6.79, (s, 2H, Mes Ph*H*), 6.92 (s, 2H, Mes Ph*H*), 6.98 (d, 2H, J = 7.55 Hz, Ph*H*), 7.23 (t, 1H, J = 7.51 Hz, Ph*H*). **^{11}B NMR** (C_6D_6): δ [ppm] = 35.53 (s, *B*Tp), –1.36 (t, J =

121.5 Hz, $B_{carborane}$), –6.67 (d, J = 135.7 Hz, $B_{carborane}$), –12.04 (m $B_{carborane}$). **^{11}B{^{1}H} NMR** (C_6D_6): δ [ppm] = 37.88 (s, *B*Tp), –0.65 (s, $B_{carborane}$), –1.62 (s, $B_{carborane}$), –6.55 (s, $B_{carborane}$), –11.36 (s, $B_{carborane}$). **^{31}P NMR** (C_6D_6): δ [ppm] = –17.64 (s, O*P*). **^{13}C{^{1}H} NMR** (C_6D_6): δ [ppm] = 144.43 (s, ortho-*C* of Ph), 140.57 (s, situ-*C* of Mes), 137.44 (s, ortho-*C*CH_3 of Mes), 136.16 (s, ortho-*C*CH_3 of Mes), 136.47 (s, para-*C*CH_3 of Mes), 128.70(s, *C*H of Mes), 128.52 (s, *C*H of Mes), 128.08 (s, para-*C*H of Ph), 127.87 (s, meta-*C*H of Ph), 26.62 (d, J = 70.16Hz, *C*H_2CH_3), 22.36 (s, Mes ortho-CH_3), 21.77 (s, Mes ortho-*C*H_3), 21.56 (s, Mes para-*C*H_3), 9.27 (d, J = 4.52 Hz, CH_2CH_3). **Elemental analysis**: calcd. for $C_{32}H_{50}B_{11}OP$, C, 63.99; H, 8.39; found C, 63.12; H, 8.45.

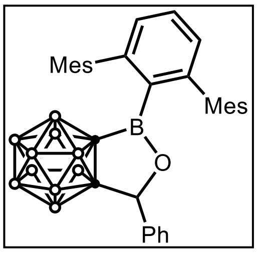

Data of 3: **^{1}H NMR** (C_6D_6): δ [ppm] = 2.15 (s, 6H, Mes C*H$_3$*), 2.16 (s, 6H, Mes C*H$_3$*), 0.60 to 3.60 (br m, 10H, B*H*), 2.22 (s, 6H, Mes C*H$_3$*), 5.29 (s, 1H, PhC*H*OR), 6.67, (d, 2H, J = 6.48 Hz, ortho-Ph*H*), 6.85 (s, 2H, Mes*H*), 6.89 (d, 2H, J = 7.64 Hz, Tp Ph*H*), 6.92 (s, 2H, Mes*H*), 7.01 to 7.06 (m, 3H, Ph*H*), 7.20 (t, 1H, J = 7.64 Hz, Ph*H*). **^{1}H{^{11}B} NMR** (C_6D_6): δ [ppm] = 1.30 (s, 1H, B*H*), 1.45 (s, 1H, B*H*), 1.65 (s, 1H, B*H*), 2.14 to 2.22 (m, 2H, B*H*), 2.30 (s, 1H, B*H*), 2.42 (s, 1H, B*H*), 2.50 (s, 1H, B*H*), 2.78 (s, 1H, B*H*), 2.82 (s, 1H, B*H*), 2.15 (s, 6H, Mes C*H$_3$*), 2.16 (s, 6H, Mes C*H$_3$*), 2.22 (s, 6H, Mes C*H$_3$*), 5.29 (s, 1H, PhC*H*OR), 6.67, (d, 2H, J = 6.48 Hz, ortho-Ph*H*), 6.85 (s, 2H, Mes*H*), 6.89 (d, 2H, J = 7.64 Hz, Tp Ph*H*), 6.92 (s, 2H, Mes*H*), 7.01 to 7.06 (m, 3H, Ph*H*), 7.20 (t, 1H, J = 7.64 Hz, Ph*H*). **^{11}B NMR** (C_6D_6): δ [ppm] = 45.75 (s, *B*Tp), –1.71 (s, $B_{carborane}$), –6.10 (s, $B_{carborane}$), -8.35 (s, $B_{carborane}$), –12.14 to -12.91 (m, $B_{carborane}$). **^{11}B{^{1}H} NMR** (C_6D_6): δ [ppm] = 46.62 (s, *B*Tp), –1.74 (s, $B_{carborane}$), –5.38 (s, $B_{carborane}$), –7.20 (s, $B_{carborane}$), –9.15 (s, $B_{carborane}$), –12.37 (s, $B_{carborane}$). **^{13}C{^{1}H} NMR** (C_6D_6): δ [ppm] = 21.20 (s, Mes *C*H_3), 21.40 (s, Mes *C*H_3), 21.95 (s, Mes *C*H_3), 86.50 (s, Ph*C*HOR), 126.52 (s, ortho-Ph*C*), 127.93 (s, Tp meta-Ph*C*), 128.17 (s, Ph*C*), 128.38 (s, Mes meta-*C*H), 128.87 (s, Mes meta-*C*H), 129.02 (s, Ph*C*), 130.56 (s, Tp para-Ph*C*), 134.60 (s, Ph*C*-CHOR), 136.85 (s, Mes *C*CH_3), 137.31 (s, Mes *C*CH_3), 137.96 (s, Mes *C*CH_3), 139.12 (s, Mes*C*-Ph), 145.46 (s, Ph*C*-Mes). **Elemental analysis**: calcd. for $C_{33}H_{41}B_{11}O$, C, 69.22; H, 7.22; found C, 69.40; H, 7.20.

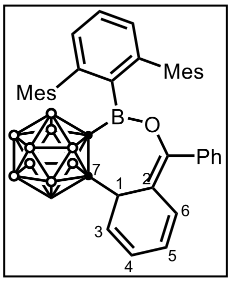

Data of 4: **^{1}H NMR** (C_6D_6): δ [ppm] = 1.40 to 3.80 (br m, 10H, B*H*), 1.70 (s, 3H, Mes C*H$_3$*), 1.99 (s, 3H, Mes C*H$_3$*), 2.12 (s, 3H, Mes C*H$_3$*), 2.17 (s, 3H, Mes C*H$_3$*), 2.29 (s, 3H, Mes C*H$_3$*), 2.37 (s, 3H, Mes C*H$_3$*), 2.65 (d, 1H, J = 5.50 Hz, C^1*H*), 5.35 (dd, 1H, J^1 = 9.64 Hz, J^2 = 5.62 Hz, C^5*H*), 5.50 (dd, 1H, J^1 = 9.33 Hz, J^2 = 5.67 Hz, C^3*H*), 5.73 (dd, 1H, J^1 = 9.38 Hz, J^2 = 5.63 Hz, C^4*H*), 5.84 (d, 1H, J = 9.67 Hz, C^6*H*), 6.67 (s, 1H, Ph*H*), 6.80 (d, 2H, J = 9.92 Hz, Ph*H*), 6.8 to 6.9 (m, 3H, Ph*H*), 7.13 to 7.21 (m, 4H, Ph*H*), 7.28 to 7.31 (m, 2H, Ph*H*). **^{1}H{^{11}B} NMR** (C_6D_6): δ [ppm] = 1.60 (s, 2H, B*H*), 1.70 (s, 3H, Mes C*H$_3$*), 1.99 (s, 3H, Mes C*H$_3$*), 2.12 (s, 3H, Mes C*H$_3$*; 1H, B*H*), 2.17 (s, 3H, Mes C*H$_3$*), 2.29 (s, 3H, Mes C*H$_3$*), 2.37 (s, 3H, Mes C*H$_3$*), 2.61 (s, 3H, B*H*), 2.65 (d, 1H, J = 5.48 Hz, C^1*H*), 2.75 (s, 1H, B*H*), 2.88 (s, 1H, B*H*), 2.92 (s, 1H, B*H*), 3.14 (s, 1H, B*H*), 5.35 (dd, 1H, J = 5.08 Hz, C^5*H*), 5.50 (dd, 1H, J = 5.00 Hz, C^2*H*), 5.73 (dd, 1H, J = 5.00 Hz, C^4*H*), 5.84 (d, 1H, J = 9.67 Hz, C^6*H*), 6.67 (s, 1H, Ph*H*), 6.80 (d, 2H, J = 9.92 Hz, Ph*H*), 6.8 to 6.9 (m, 3H, Ph*H*), 7.13 to 7.21 (m, 4H, Ph*H*), 7.28 to 7.31 (m, 2H, Ph*H*). **^{11}B NMR** (C_6D_6): δ [ppm] = 43.32 (s, C*B*O), 2.76 (m, $B_{carborane}$), –4.11 (m, $B_{carborane}$), –9.73 (m, $B_{carborane}$). **^{11}B{^{1}H} NMR** (C_6D_6): δ [ppm] = 47.15 (s, C*B*O), 3.41 (s, $B_{carborane}$), –3.37 (s, $B_{carborane}$), –7.77 (s, $B_{carborane}$), –8.83 (s, $B_{carborane}$). **^{13}C{^{1}H} NMR** (C_6D_6): δ [ppm] = 21.02 (s, Mes *C*H_3), 21.13 (s, Mes *C*H_3), 21.23 (s, Mes *C*H_3), 21.80 (s, Mes *C*H_3), 21.92 (s, Mes *C*H_3), 22.55 (s, Mes *C*H_3), 41.25 (s, C^1), 71.42 (s, C^7), 89.28 (s, C^2), 120.60 (s, C^2*C*O), 122.89 (s, C^6), 123.05 (s, C^5), 124.60 (s, C^3), 124.68 (s, C^4), 128.16 (s, C_{Ar}), 128.51 (s, C_{Ar}), 128.59 (s, C_{Ar}), 128.77 (s, C_{Ar}), 128.80 (s, C_{Ar}), 129.13 (s, C_{Ar}), 129.28 (s, C_{Ar}), 129.36 (s, C_{Ar}), 129.75 (s, C_{Ar}), 130.09 (s, C_{Ar}),

133.62 (s, C_{Ar}), 137.41 (s, C_{Ar}), 137.65 (s, C_{Ar}), 137.73 (s, C_{Ar}), 137.92 (s, C_{Ar}), 138.79 (s, C_{Ar}), 139.01 (s, C_{Ar}), 139.40 (s, C_{Ar}), 144.01 (s, C_{Ar}), 144.39 (s, C_{Ar}), 151.27 (s, C_{Ar}). **Elemental analysis**: calcd. for $C_{39}H45B_{11}O$, C, 72.21; H, 6.99; found C, 72.90; H, 7.15.

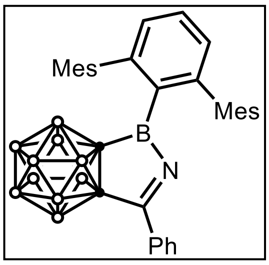

Data of 5: **^{1}H NMR** (C_6D_6): δ [ppm] = 2.06 (s, 6H, Mes para-C*H₃*), 2.27 (s, 12H, Mes ortho-C*H₃*), 0.75 to 3.67 (br m, 10H, B*H*), 6.76 (s, 4H, Mes meta-C*H*), 6.87 (t, 2H, J = 7.74 Hz, Ph*H*), 6.97 (d, 2H, J = 7.64 Hz, Ph*H*), 7.26 (t, 1H, J = 7.62 Hz, Tp Ph para-C*H*), 8.16 (d, 2H, J = 8.00 Hz, Tp Ph meta-C*H*). **^{1}H{^{11}B} NMR** (C_6D_6): δ [ppm] = 1.75 (s, 2H, B*H*), 2.06 (s, 6H, Mes para-CH_3), 2.27 (s, 14H, Mes ortho-CH_3 and B*H*), 2.56 (s, 2H, B*H*), 2.82 (s, 2H, B*H*), 2.98 (s, 1H, B*H*), 3.06 (s, 1H, B*H*). 6.76 (s, 4H, Mes meta-C*H*), 6.87 (t, 2H, J = 7.74 Hz, Ph*H*), 6.97 (d, 2H, J = 7.64 Hz, Ph*H*), 7.26 (t, 1H, J = 7.62 Hz, Tp Ph para-C*H*), 8.16 (d, 2H, J = 8.00 Hz, Tp Ph meta-C*H*). **^{11}B NMR** (C_6D_6): δ [ppm] = 62.60 (s, C*B*N), –2.29 (s, $B_{carborane}$), –5.06 (s, $B_{carborane}$), –8.48 (s, $B_{carborane}$), –12.86 (s, $B_{carborane}$), –14.11 (s, $B_{carborane}$). **^{11}B{^{1}H} NMR** (C_6D_6): δ [ppm] = 61.22 (s, C*B*N), 0.48 (s, $B_{carborane}$), –1.78 (s, $B_{carborane}$), –5.46 (s, $B_{carborane}$), –9.03 (s, $B_{carborane}$), –12.22 (s, $B_{carborane}$). **^{13}C{^{1}H} NMR** (C_6D_6): δ [ppm] = 21.07 (s, Mes para-CH_3), 21.73 (s, Mes ortho-CH_3), 127.94 (s, Ph*C*H), 128.17 (s, Ph*C*H), 128.84 (s, Mes meta-*C*H), 128.93 (s, Mes para-*C*CH_3), 130.57 (s, Tp para-*C*H), 131.68 (s, Tp meta-*C*H), 132.21 (s, Ph*C*-C), 135.22 (s, Ph*C*H), 137.08 (s, Mes ortho-*C*CH_3), 137.90 (s, Mes*C*-Ph), 138.95 (s, N*C*), 145.13 (s, Ph*C*-Mes), 187.28 (s, B*C*). **Elemental analysis**: calcd. for $C_{33}H_{40}B_{11}N$, C, 69.58; H, 7.08, N, 2.46; found C, 69.17; H, 7.11, N, 2.37.

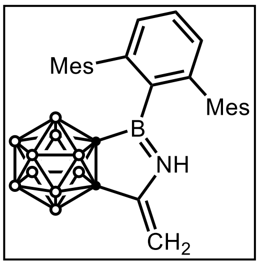

Data of 6: **^{1}H NMR** (C_6D_6): δ [ppm] = 1.8 to 3.8 (br m, 10H, B*H*), 2.00 (s, 12H, Mes ortho-CH_3), 2.13 (s, 6H, Mes para-CH_3), 3.67 (d, 1H, J = 2.34 Hz, CH_2), 4.06 (d, 1H, J = 1.94 Hz, CH_2), 5.85 (s, 1H, N*H*), 6.79 (s, 4H, Mes meta-C*H*), 6.87 (d, 2H, J = 7.60 Hz, Ph meta-C*H*), 7.17 (t, 1H, J = 7.43 Hz, Ph para-C*H*). **^{1}H{^{11}B} NMR** (C_6D_6): δ [ppm] = 1.42 (s, 2H, B*H*), 2.00 (s, 12H, Mes ortho-CH_3), 2.13 (s, 6H, Mes para-CH_3), 2.42 (s, 2H, B*H*), 2.73 (s, 2H, B*H*), 2.80 (s, 2H, B*H*), 3.67 (s, 1H, CH_2), 4.07 (s, 1H, CH_2), 5.85 (s, 1H, N*H*), 6.79 (s, 4H, Mes meta-C*H*), 6.87 (d, 2H, J = 7.60 Hz, Ph meta-C*H*), 7.17 (t, 1H, J = 7.43 Hz, Ph para-C*H*). **^{11}B NMR** (C_6D_6): δ [ppm] = 42.11 (s, *B*Tp), –4.19 (s, $B_{carborane}$), –5.38 (s, $B_{carborane}$), –6.73 (s, $B_{carborane}$), –7.89 (s, $B_{carborane}$), –9.70 (s, $B_{carborane}$), –12.29 (s, $B_{carborane}$). **^{11}B{^{1}H} NMR** (C_6D_6): δ [ppm] = 41.65 (s, *B*Tp), –2.65 (s, $B_{carborane}$), –4.64 (s, $B_{carborane}$), –7.07 (s, $B_{carborane}$), –8.82 (s, $B_{carborane}$), –11.40 (s, $B_{carborane}$), –14.83 (s, $B_{carborane}$). **^{13}C{^{1}H} NMR** (C_6D_6): δ [ppm] = 21.06 (s, Mes para-CH_3), 21.44 (s, Mes ortho- CH_3), 76.16 (s, $C_{carborane}$), 90.99 (s, CH_2), 128.53 (s, Ph meta-*C*H), 128.86 (s, Mes meta-*C*H), 130.31 (s, Ph para-*C*H), 135.87 (s, Mes ortho-*C*CH_3), 137.80 (s, Mes para- *C*CH_3), 139.11 (s, Mes *C*Ph), 144.33 (s, N*C*), 145.77 (s, Ph *C*-Mes). **Elemental analysis**: calcd. for $C_{28}H_{38}B_{11}N$, C, 66.26; H, 7.55; N, 2.76 found C, 66.20; H, 7.44, N, 2.72.

NMR spectra

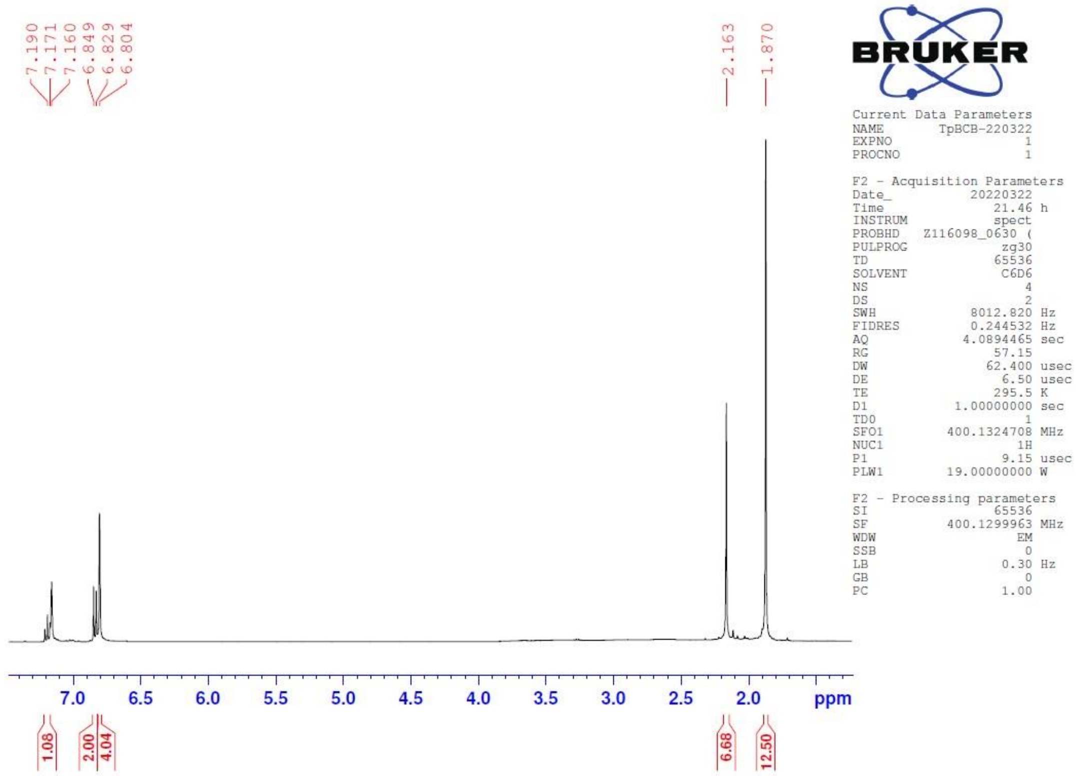

Figure S1. ^{1}H NMR spectrum of **1** in C_6D_6

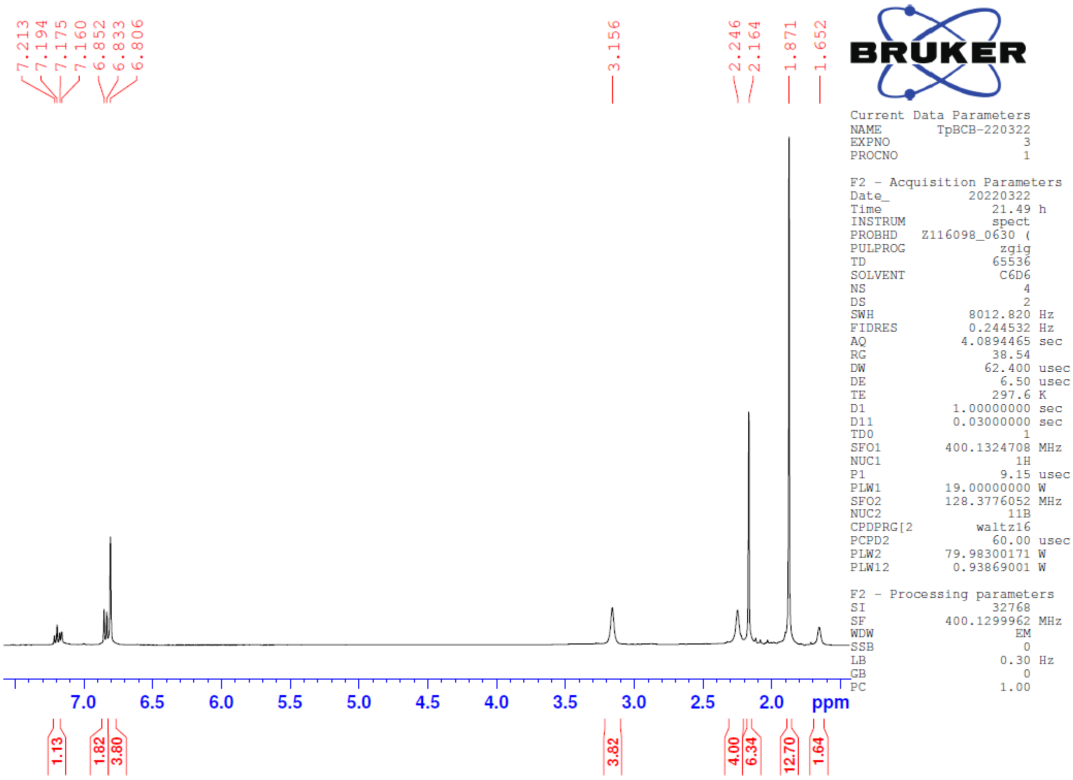

Figure S2. $^{1}H\{^{11}B\}$ NMR spectrum of **1** in C_6D_6

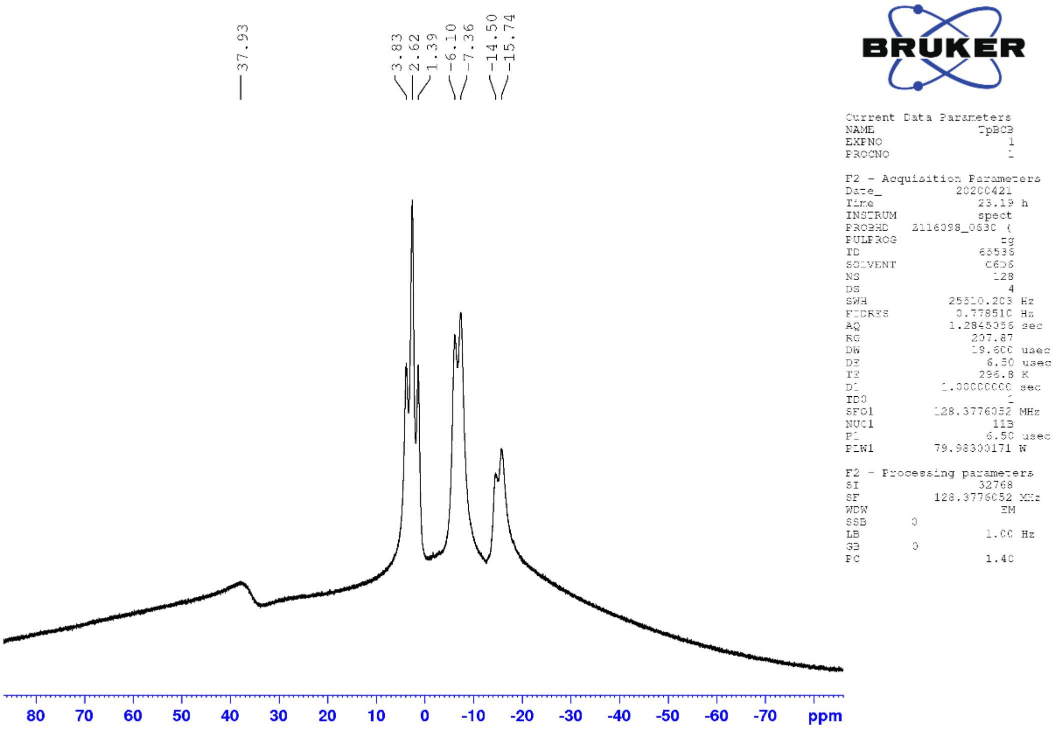

Figure S3. ^{11}B NMR spectrum of **1** in C_6D_6

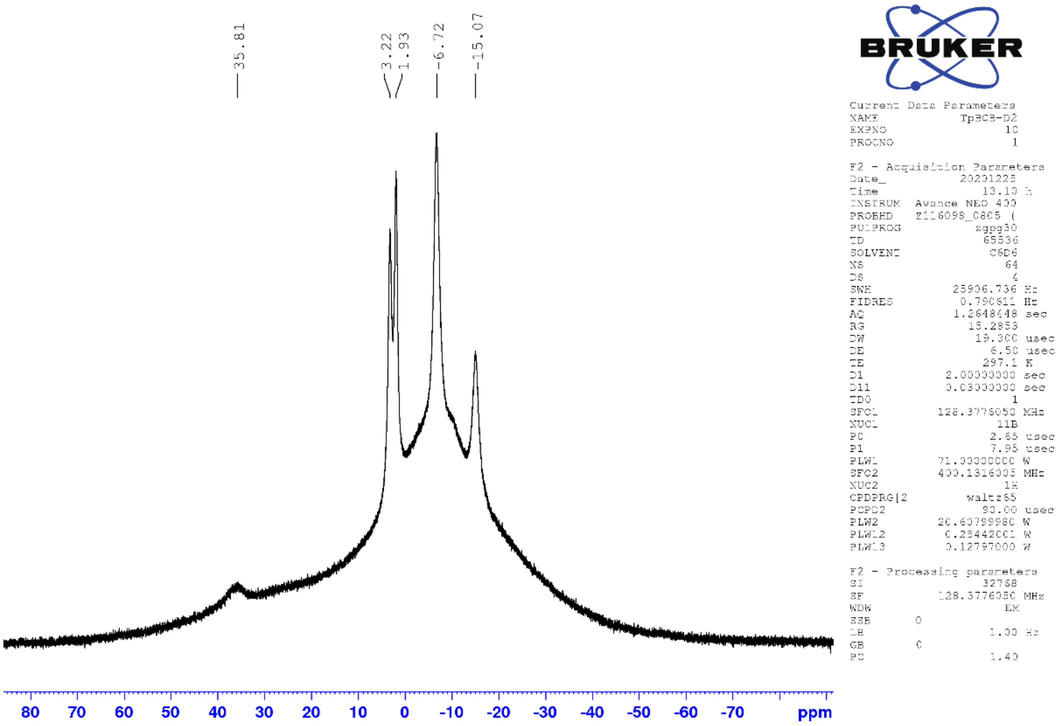

Figure S4. $^{11}B\{^1H\}$ NMR spectrum of **1** in C_6D_6

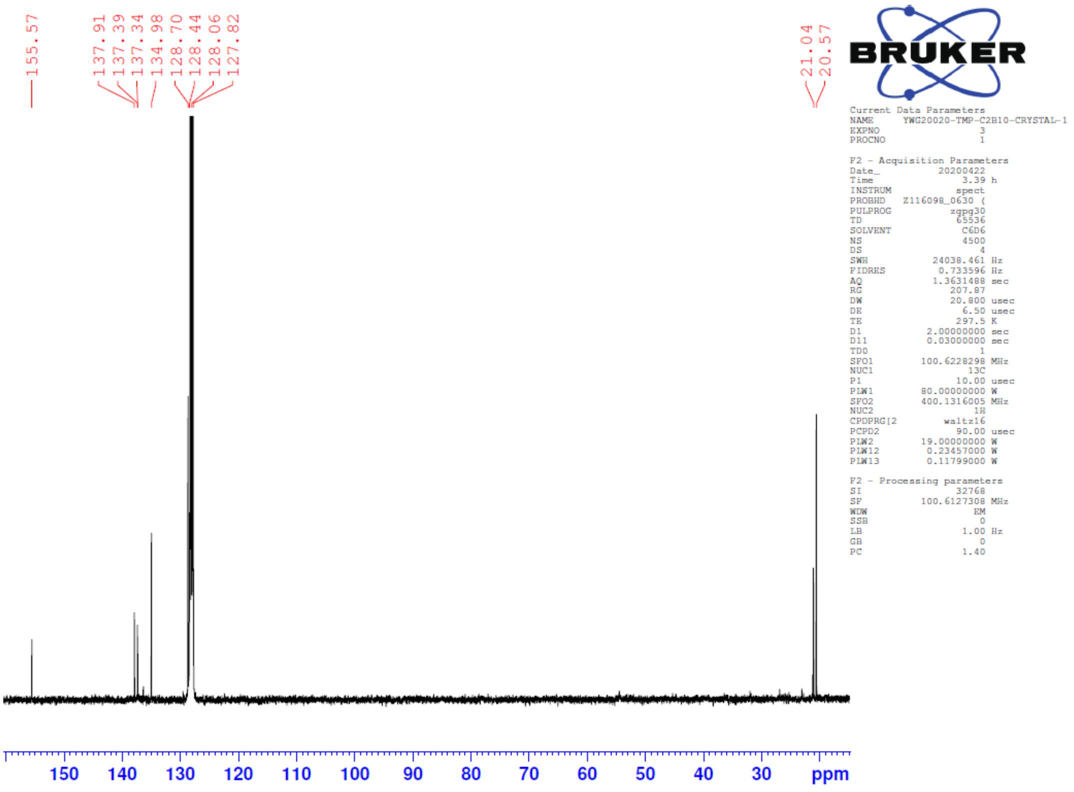

Figure S5. $^{13}C\{^{1}H\}$ NMR spectrum of **1** in C_6D_6

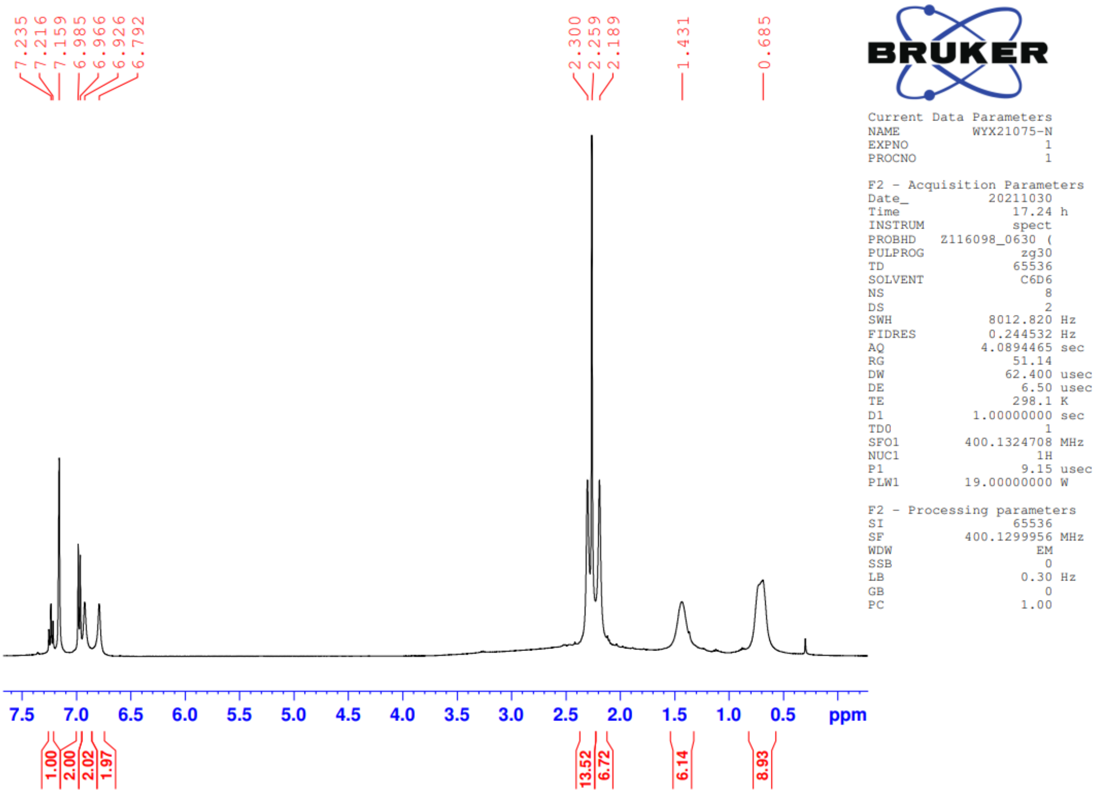

Figure S6. ^{1}H NMR spectrum of **2** in C_6D_6

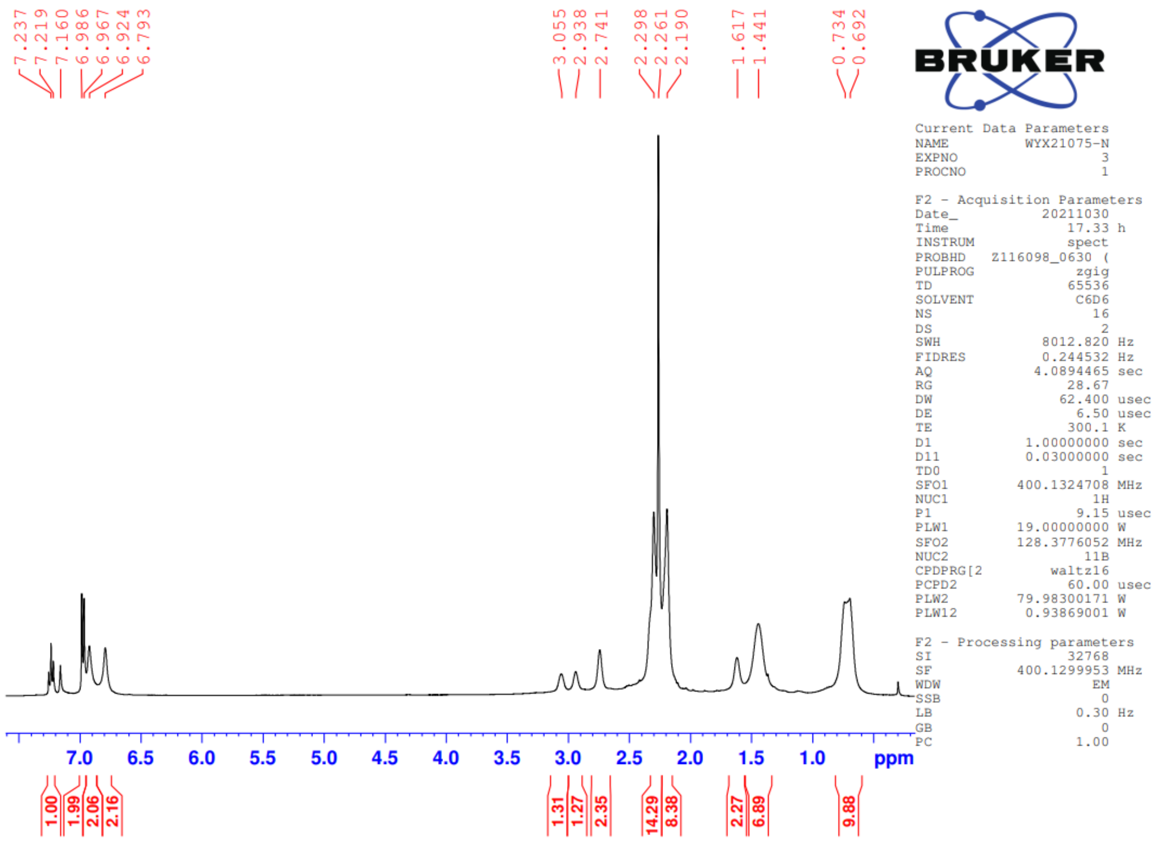

Figure S7. $^{1}H\{^{11}B\}$ NMR spectrum of **2** in C_6D_6

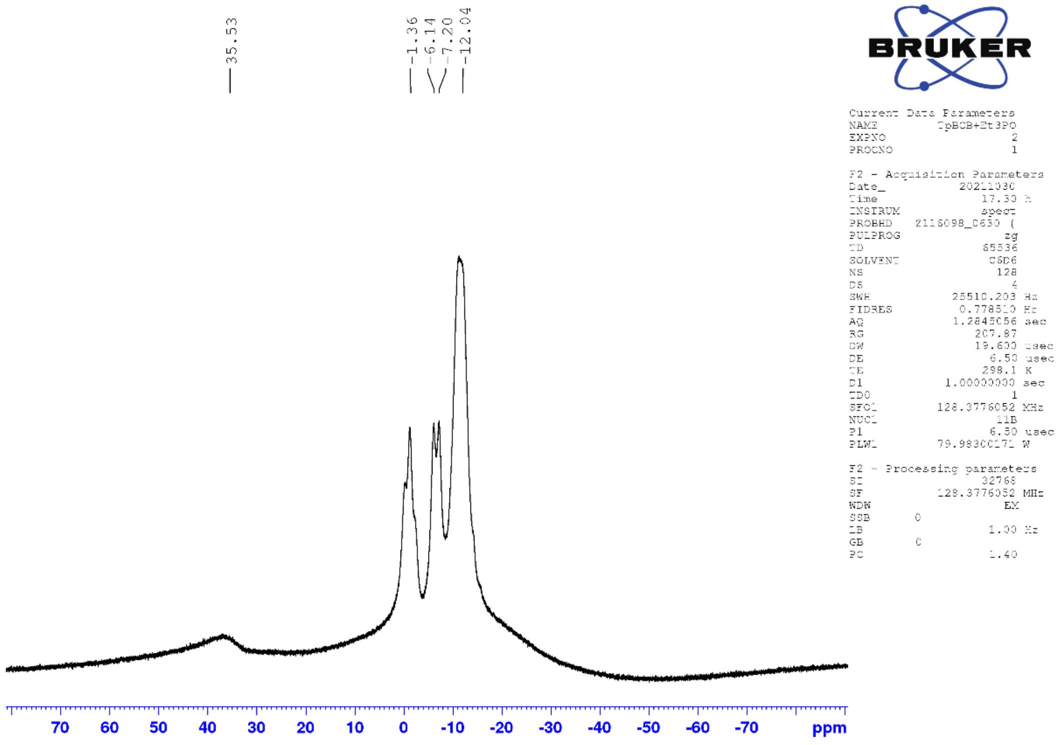

Figure S8. ^{11}B NMR spectrum of **2** in C_6D_6

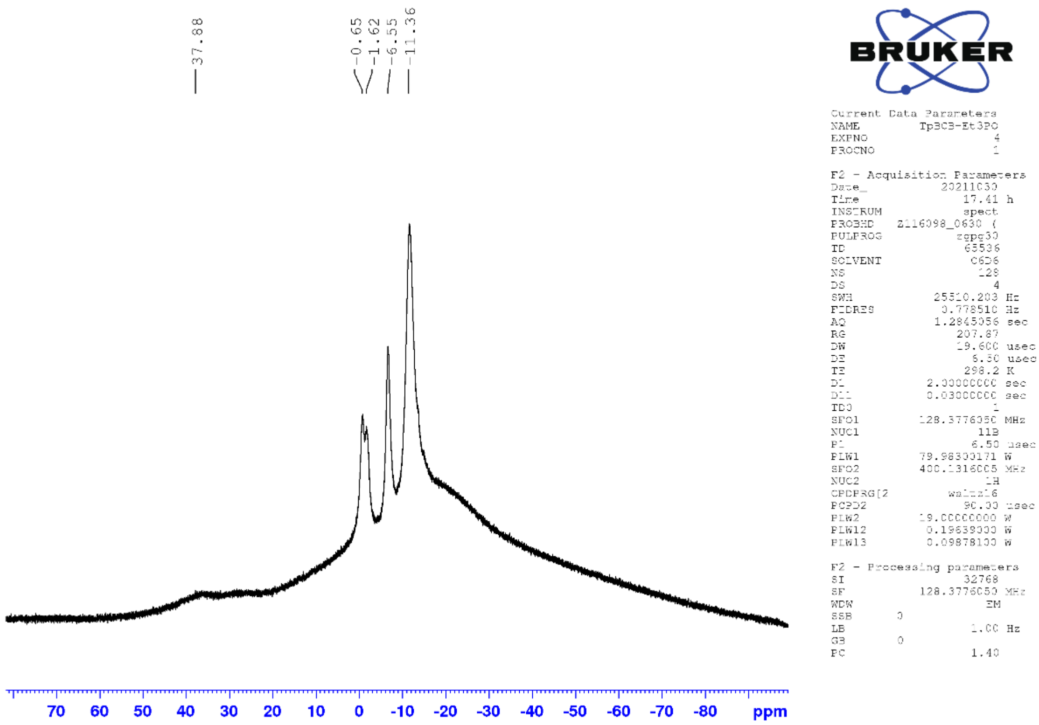

Figure S9. $^{11}B\{^{1}H\}$ NMR spectrum of **2** in C_6D_6

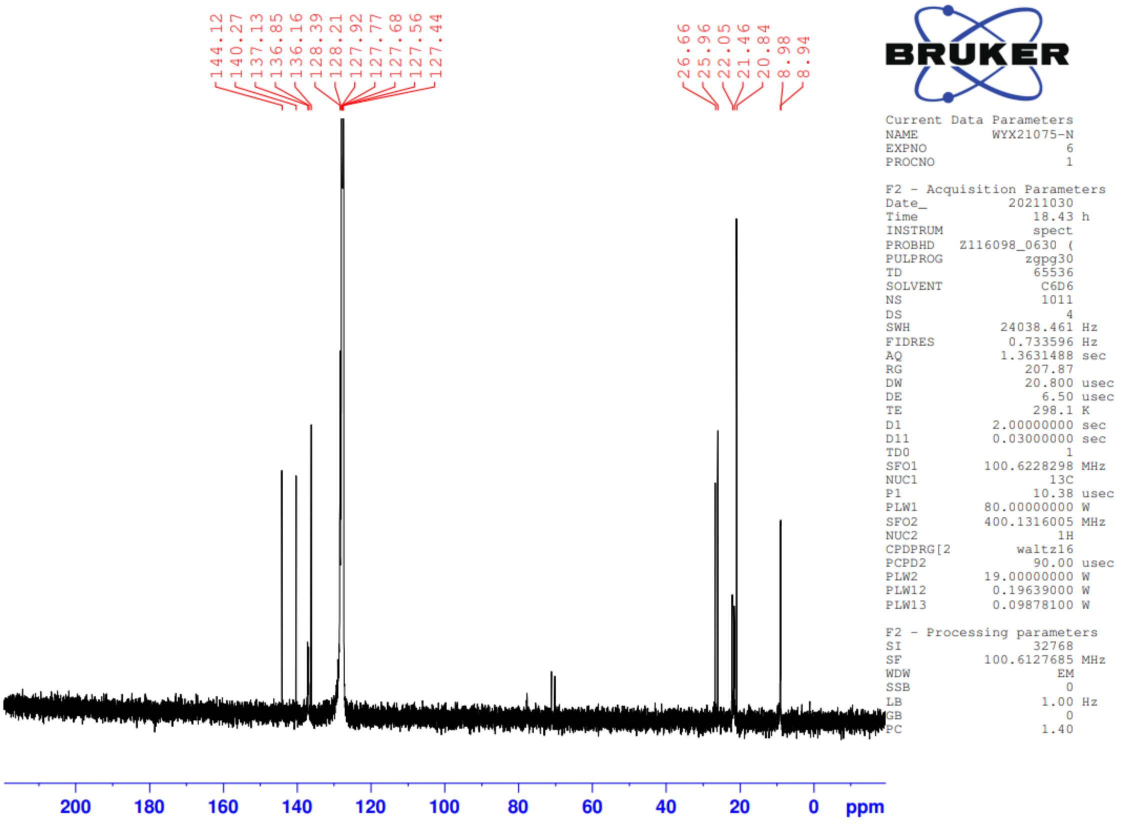

Figure S10. $^{13}C\{^{1}H\}$ NMR spectrum of **2** in C_6D_6

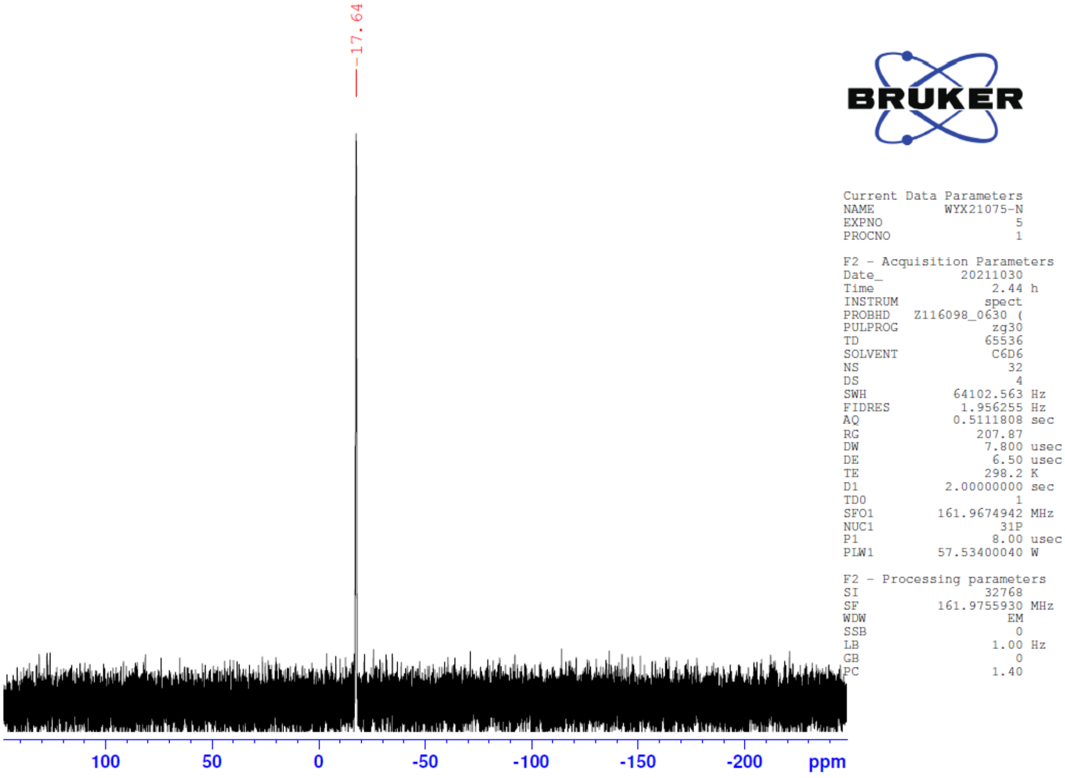

Figure S11. ^{31}P NMR spectrum of **2** in C_6D_6

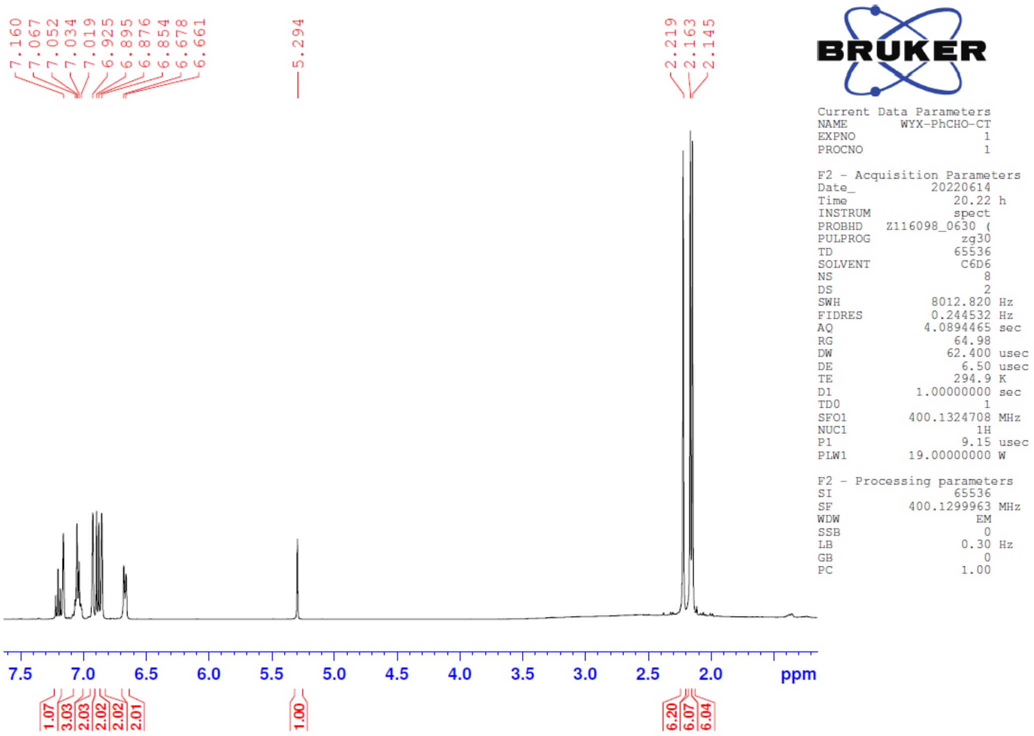

Figure S12. ^{1}H NMR spectrum of **3** in C_6D_6

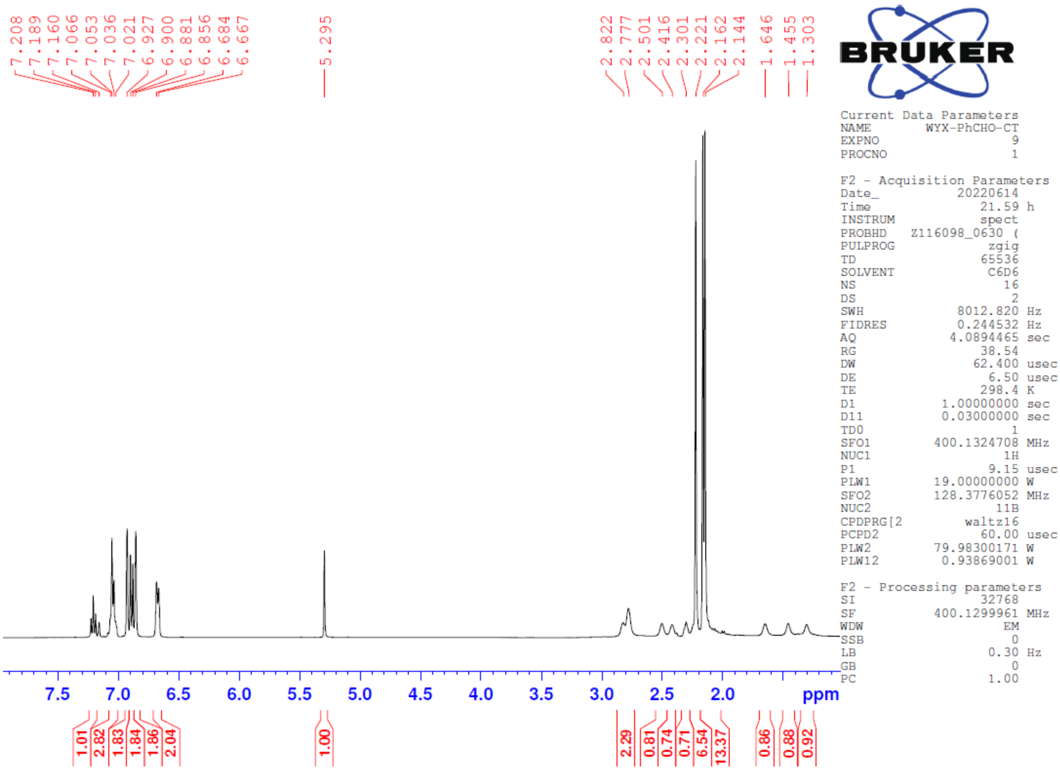

Figure S13. $^{1}H\{^{11}B\}$ NMR spectrum of **3** in C_6D_6

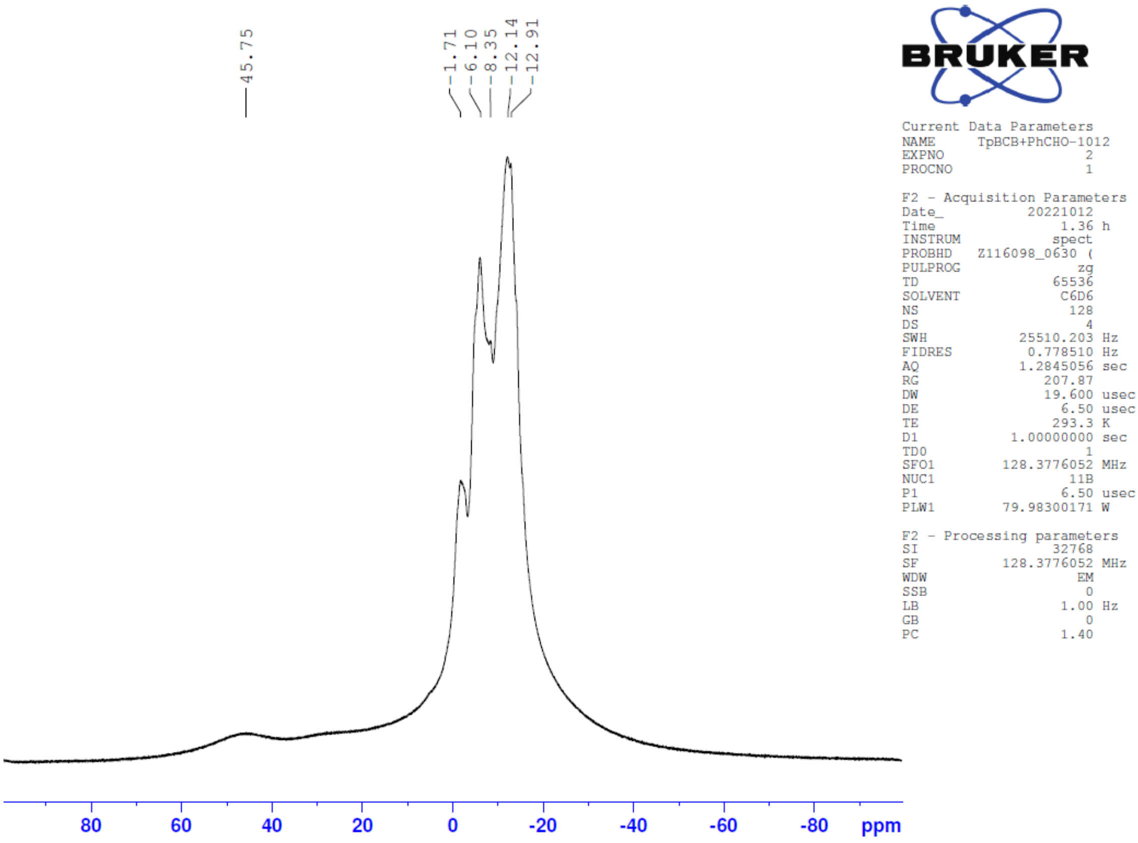

Figure S14. ^{11}B NMR spectrum of **3** in C_6D_6

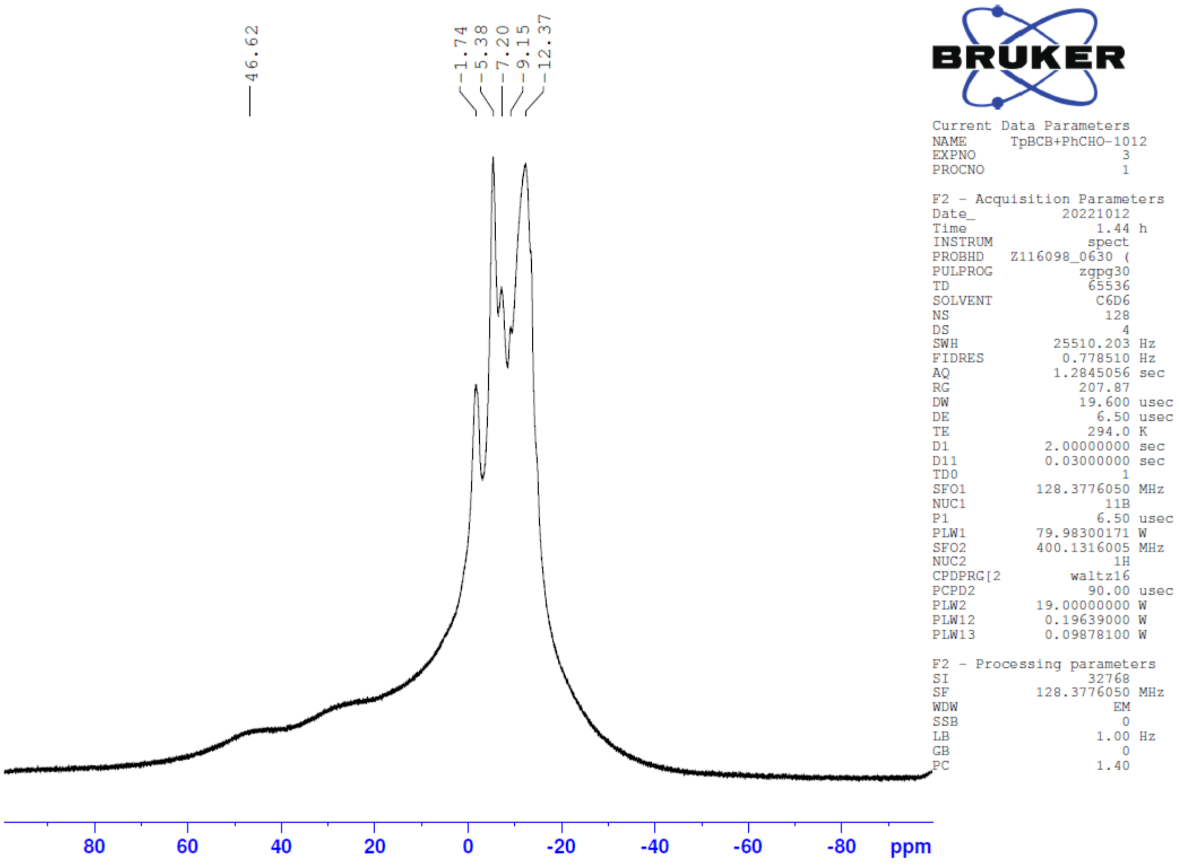

Figure S15. $^{11}B\{^1H\}$ NMR spectrum of **3** in C_6D_6

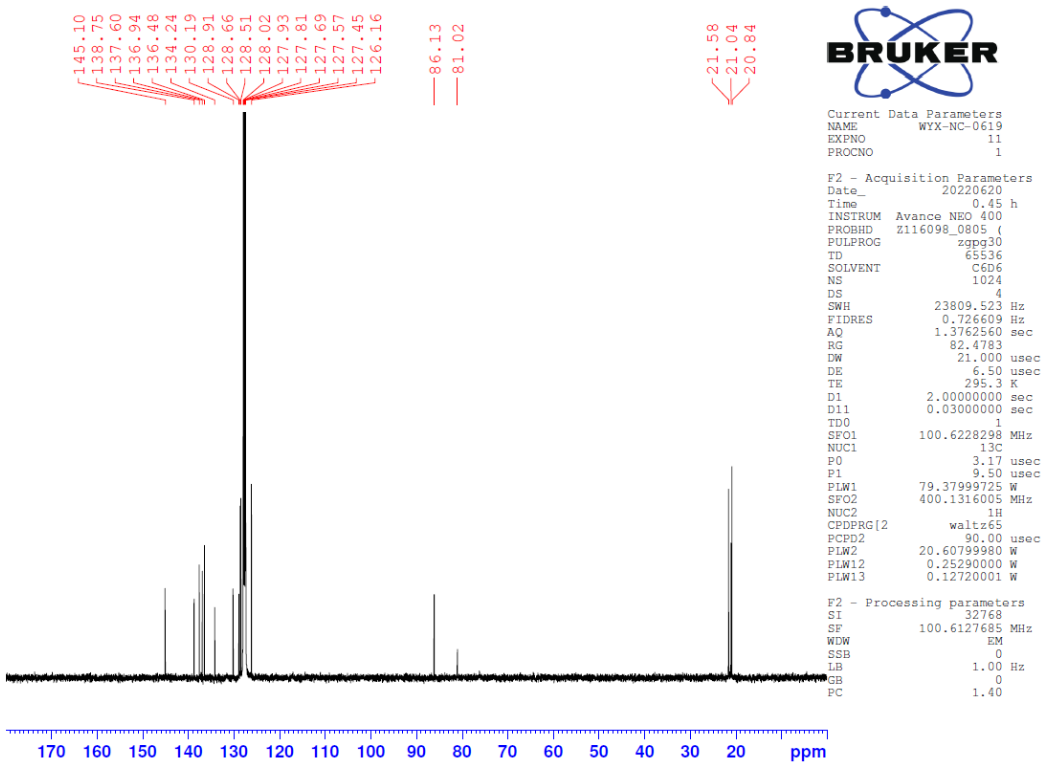

Figure S16. $^{13}C\{^1H\}$ NMR spectrum of **3** in C_6D_6

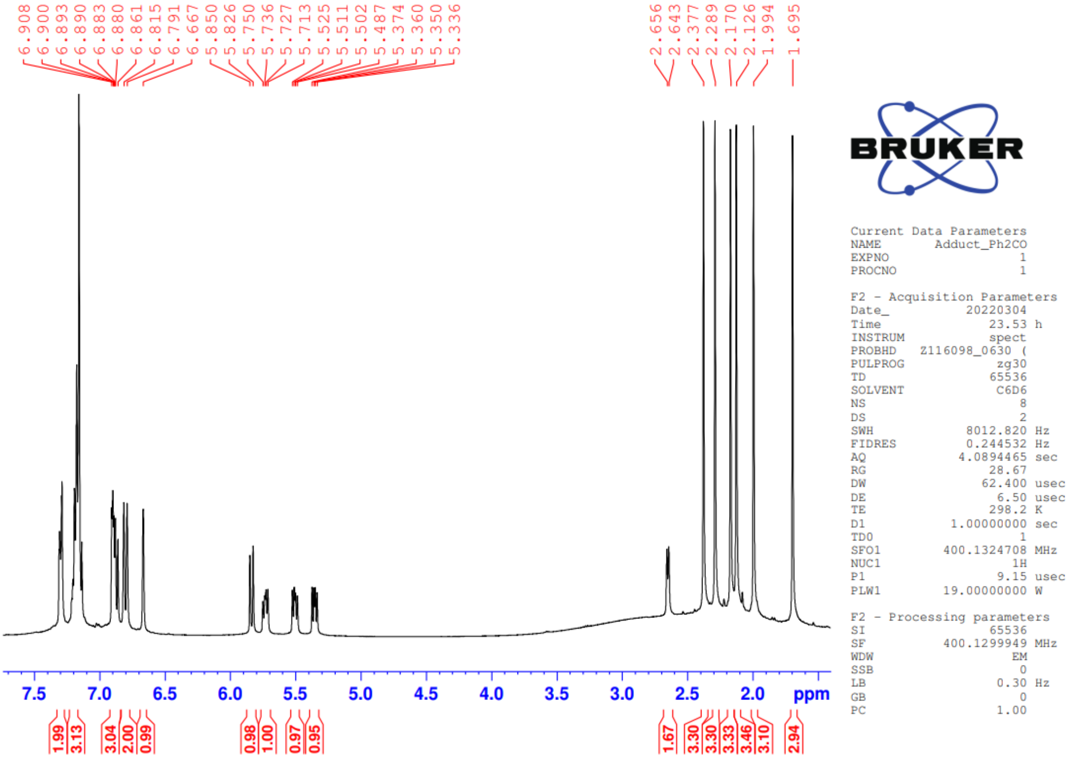

Figure S17. ^{1}H NMR spectrum of **4** in C_6D_6

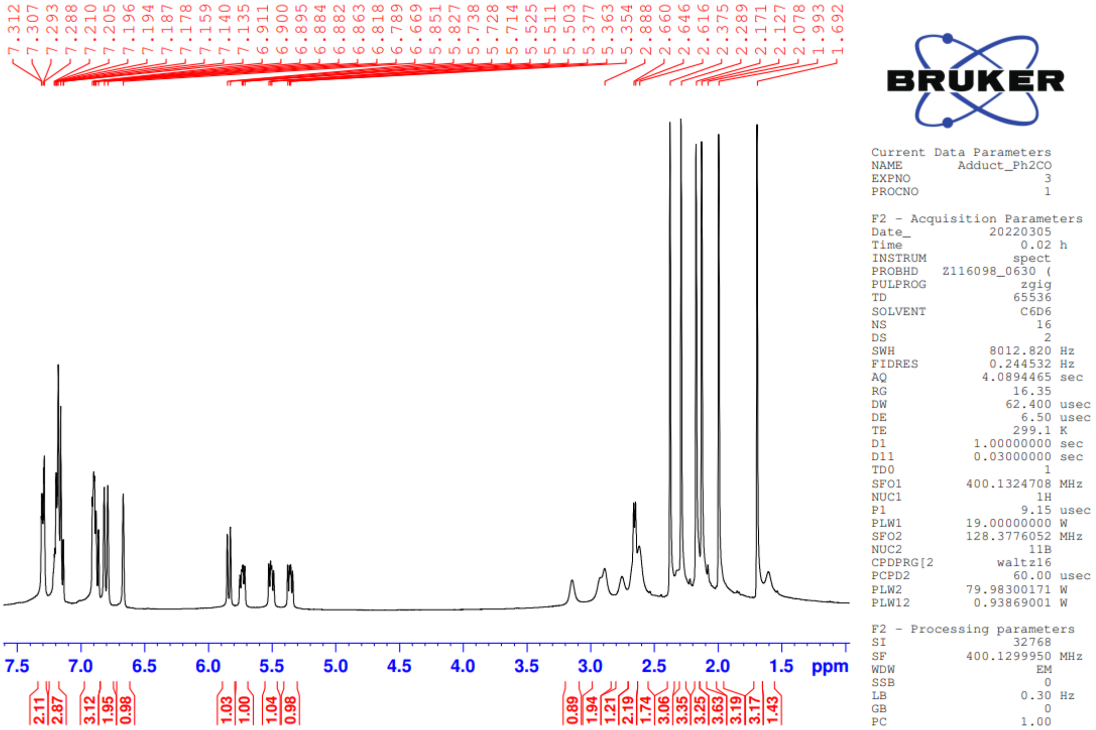

Figure S18. ^{1}H{^{11}B} NMR spectrum of **4** in C_6D_6

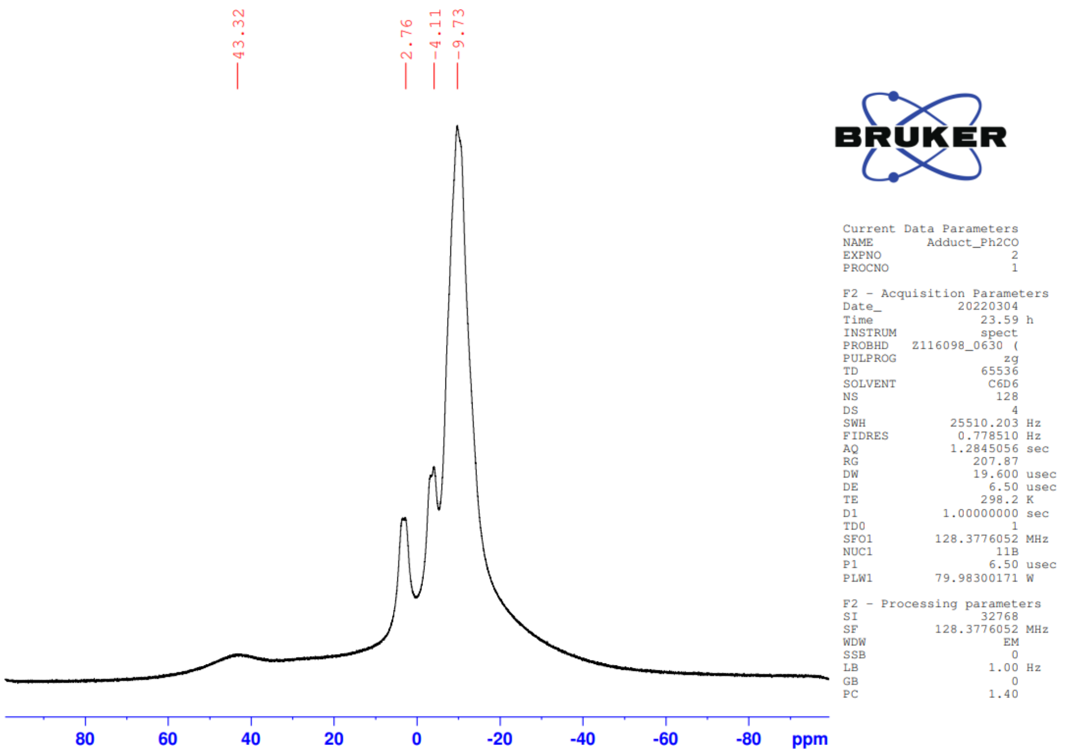

Figure S19. ^{11}B NMR spectrum of **4** in C_6D_6

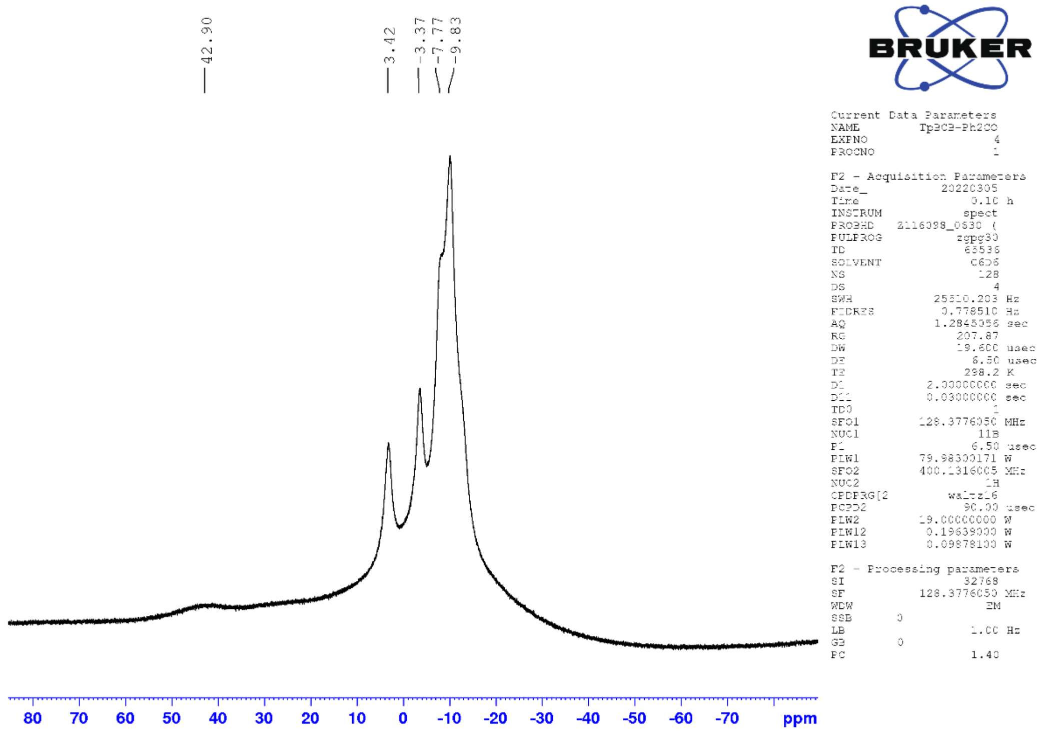

Figure S20. ^{11}B{^{1}H} NMR spectrum of **4** in C_6D_6

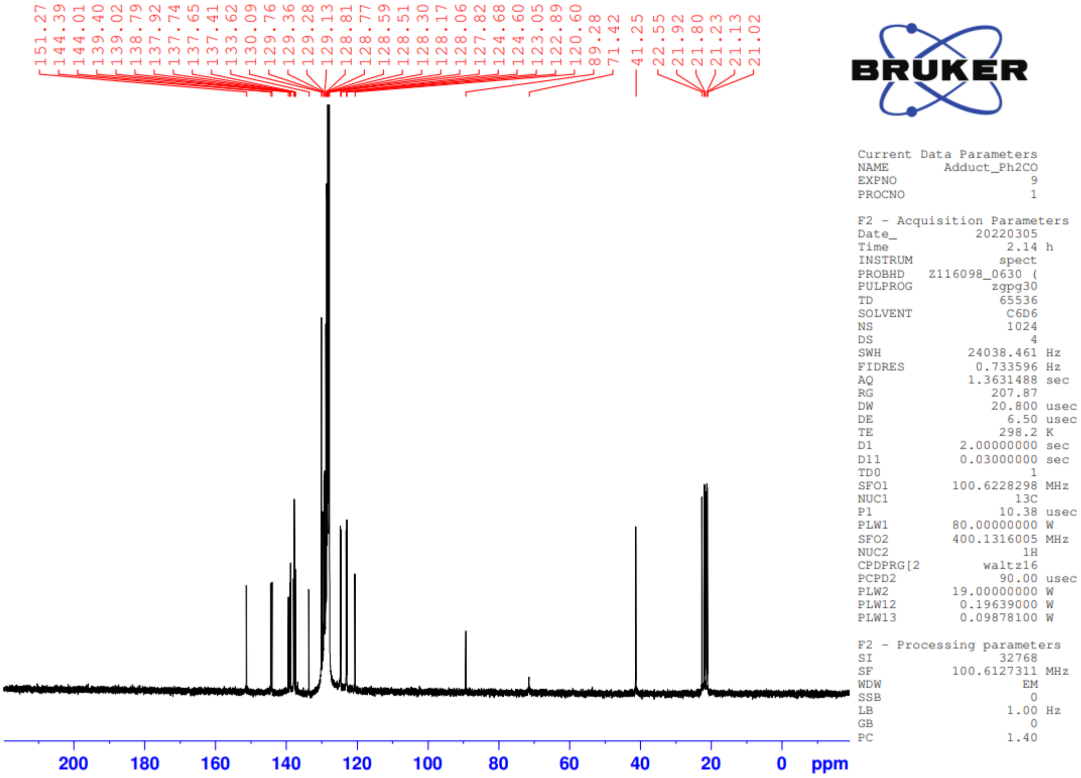

Figure S21 $^{13}C\{^{1}H\}$ NMR spectrum of **4** in C_6D_6

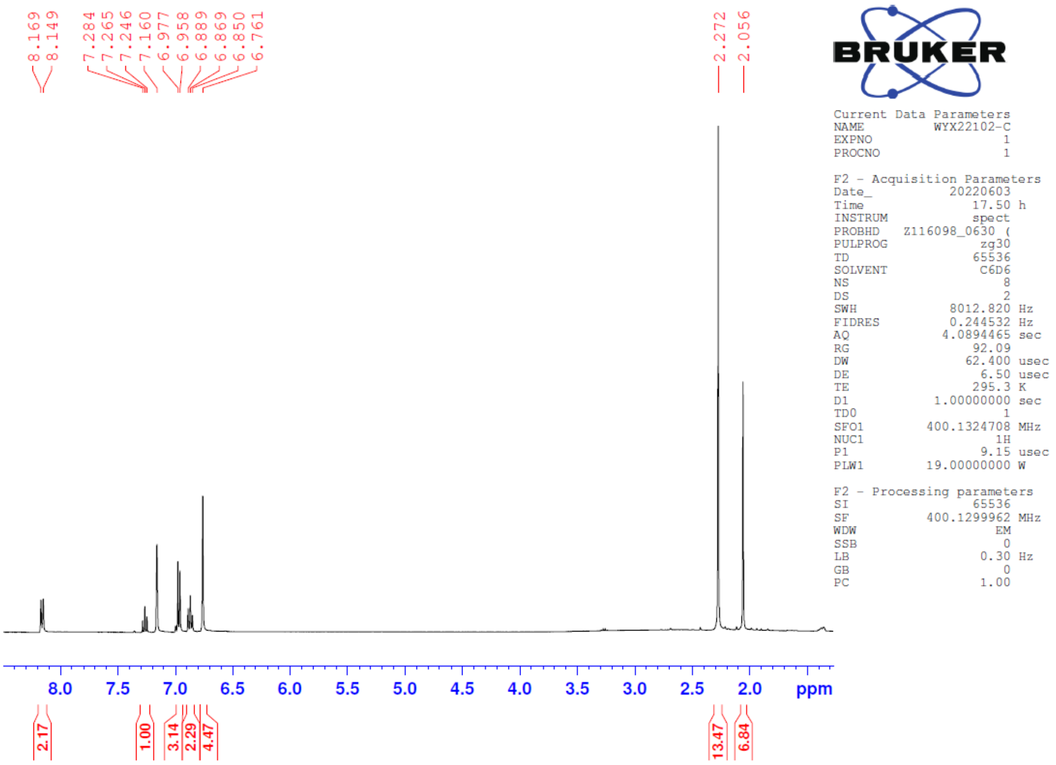

Figure S22. ^{1}H NMR spectrum of **5** in C_6D_6

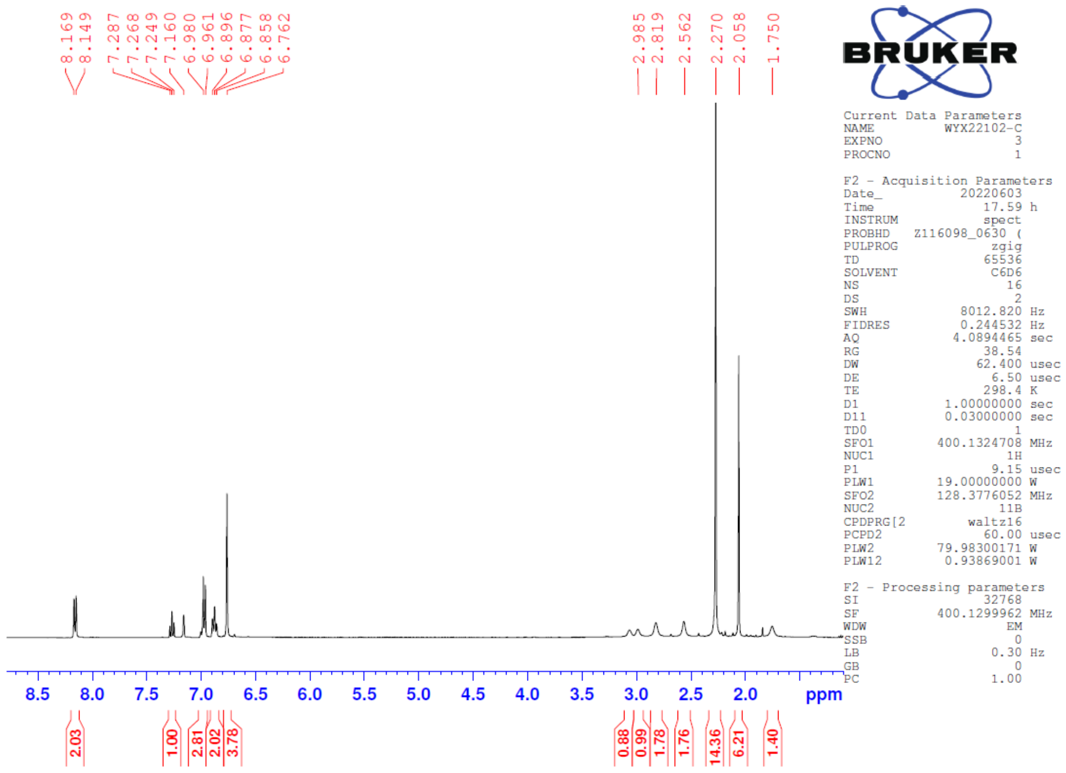

Figure S23. $^{1}H\{^{11}B\}$ NMR spectrum of **5** in C_6D_6

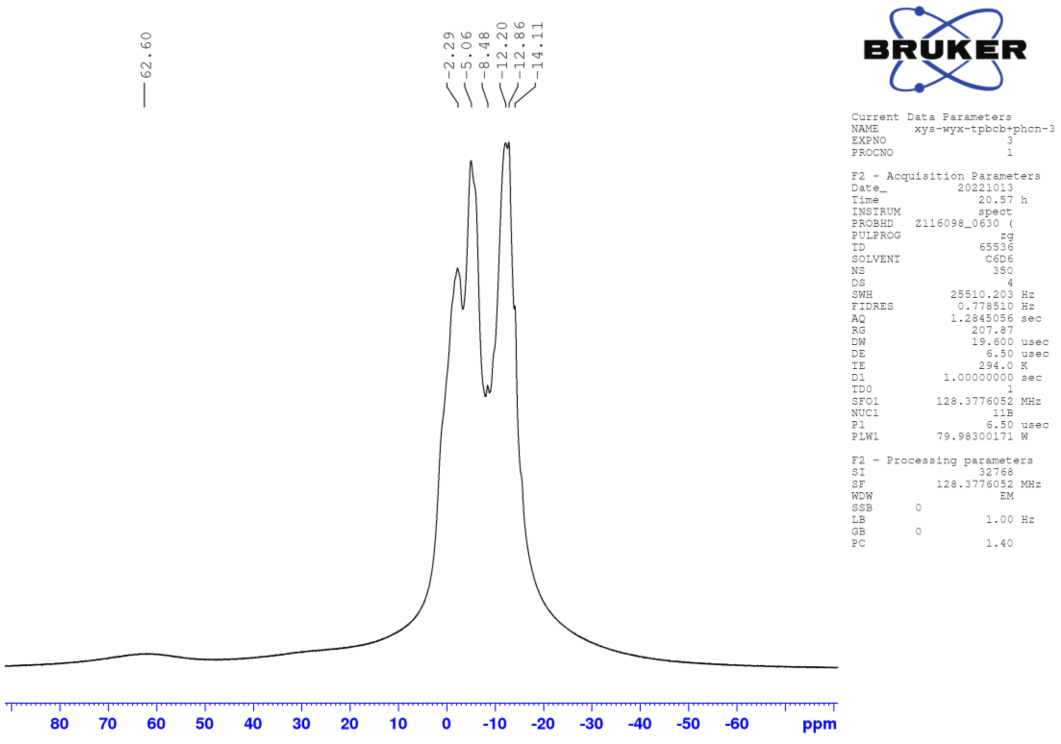

Figure S24. ^{11}B NMR spectrum of **5** in C_6D_6

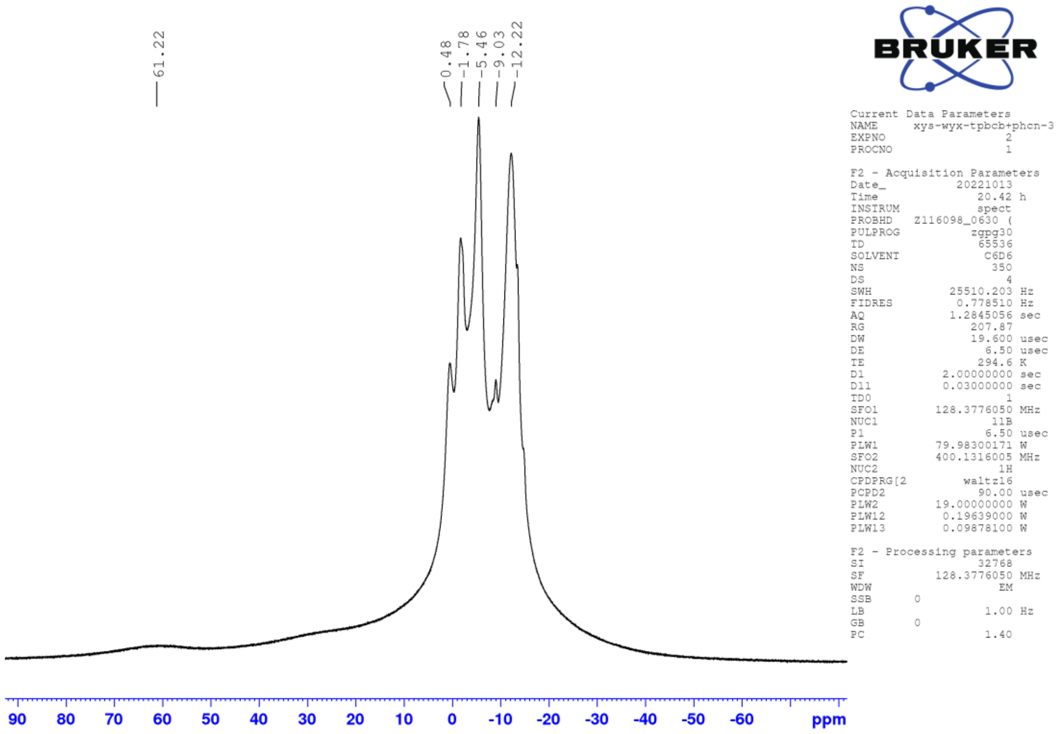

Figure S25. ${}^{11}B\{{}^{1}H\}$ NMR spectrum of **5** in C_6D_6

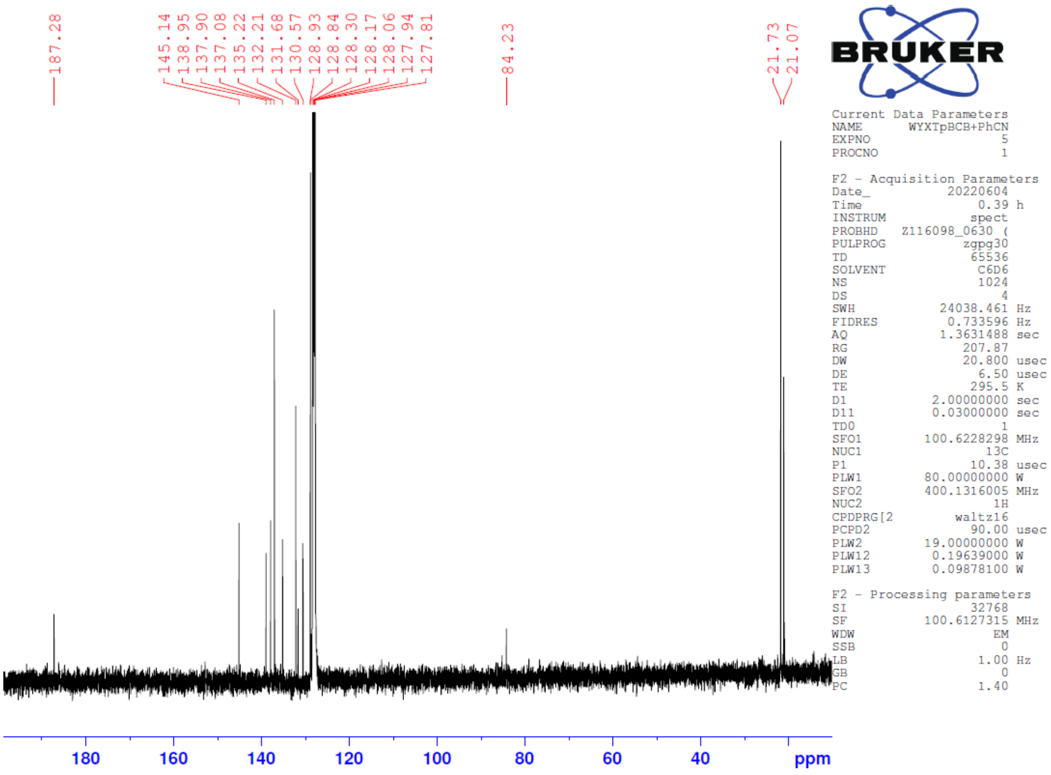

Figure S26. ${}^{13}C\{{}^{1}H\}$ NMR spectrum of **5** in C_6D_6

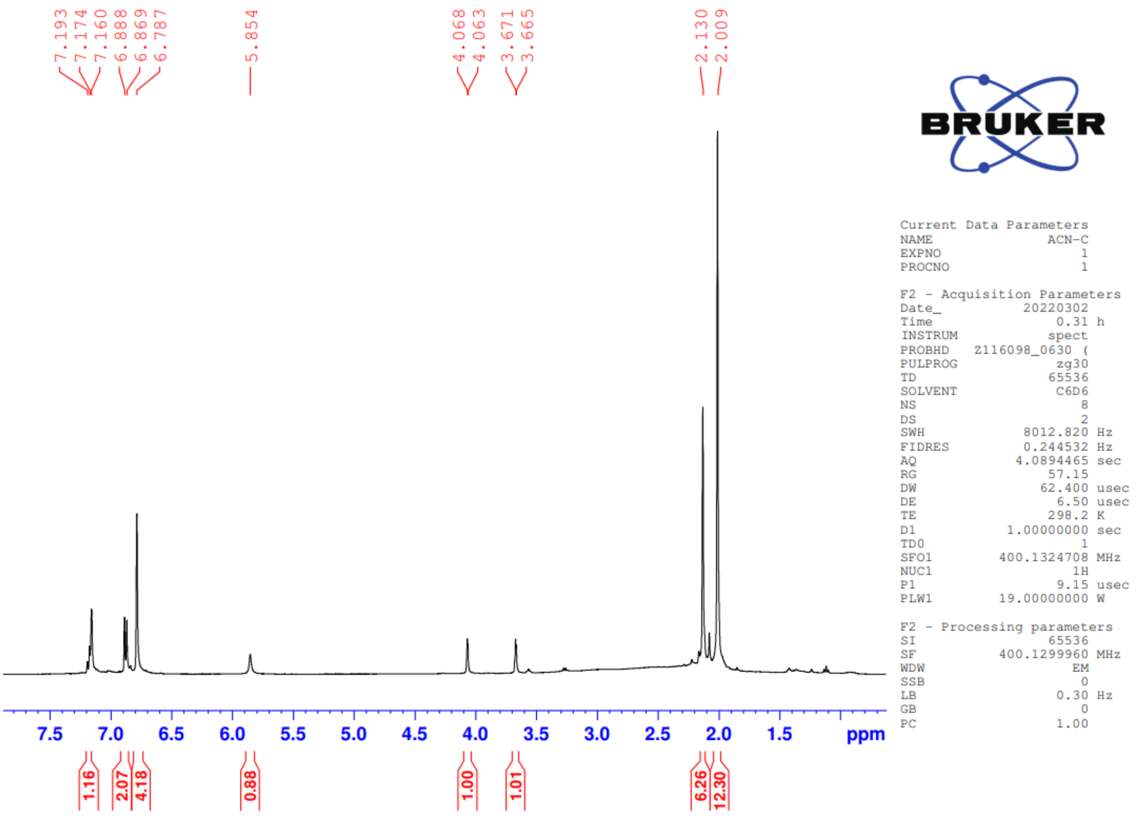

Figure S27. ^{1}H NMR spectrum of **6** in C_6D_6

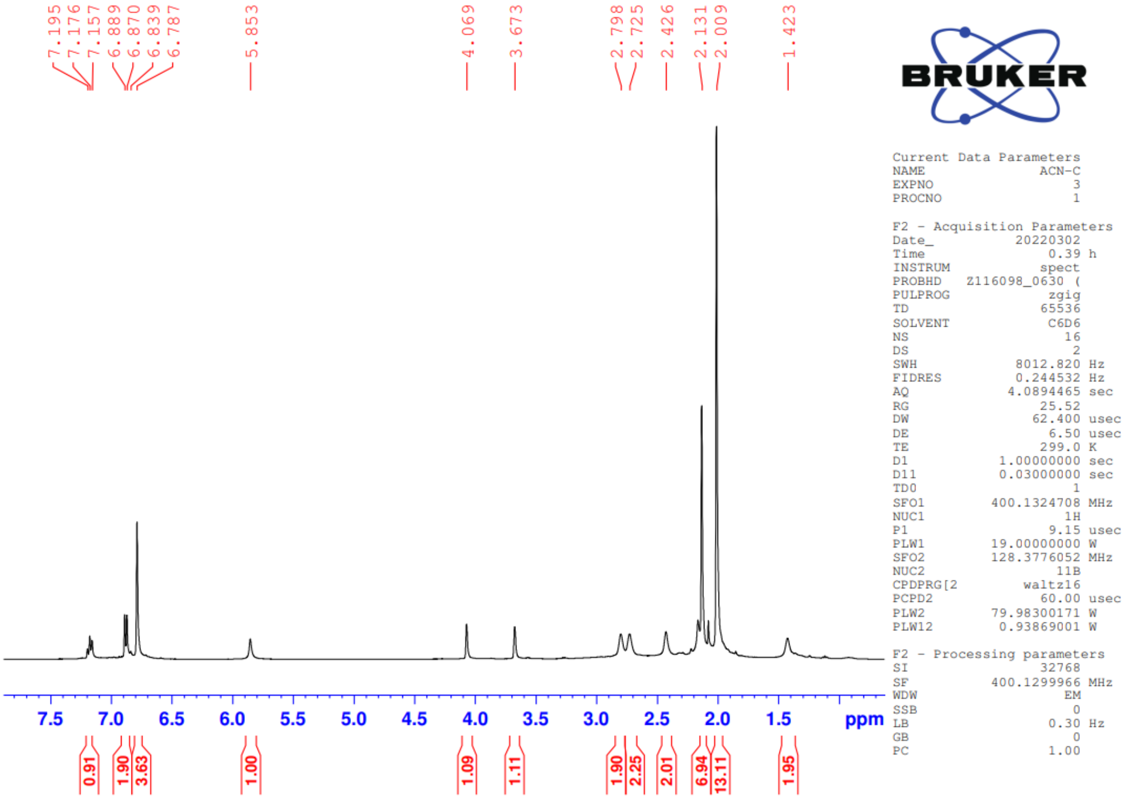

Figure S28. ^{1}H{^{11}B} NMR spectrum of **6** in C_6D_6

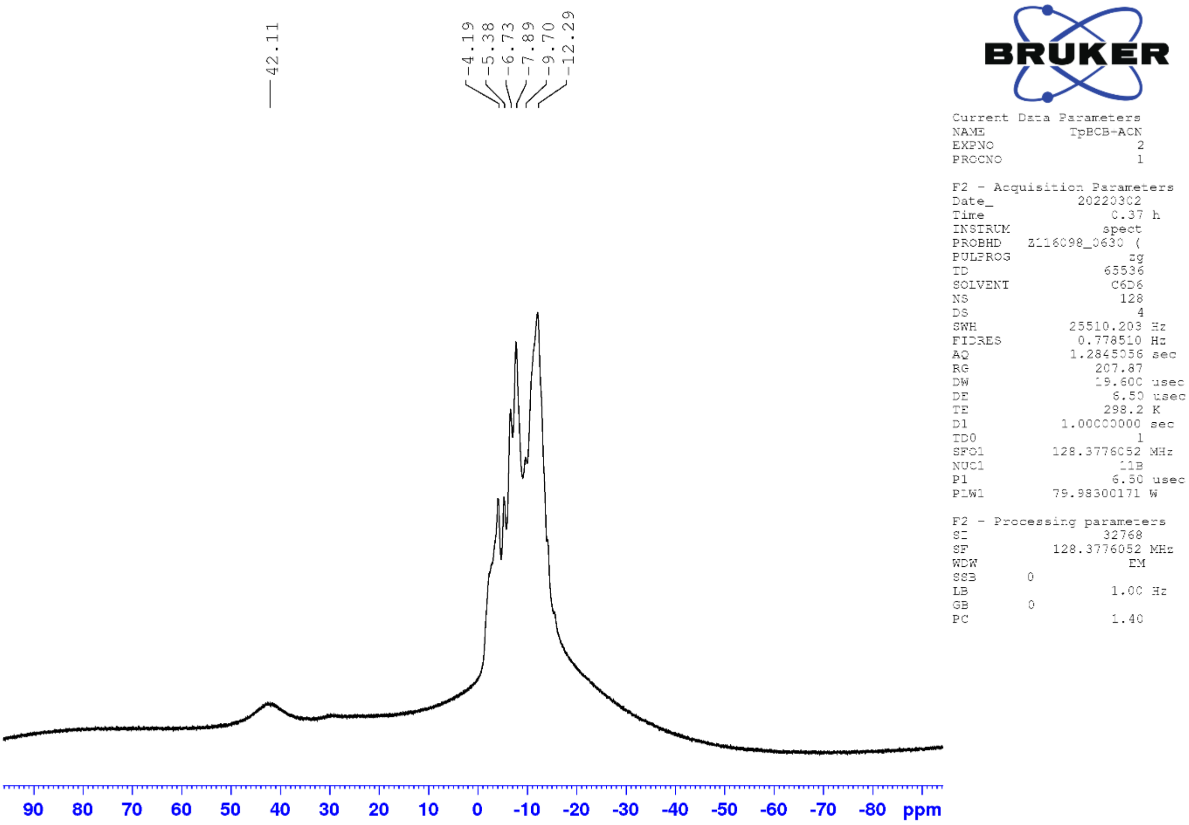

Figure S29. ^{11}B NMR spectrum of **6** in C_6D_6

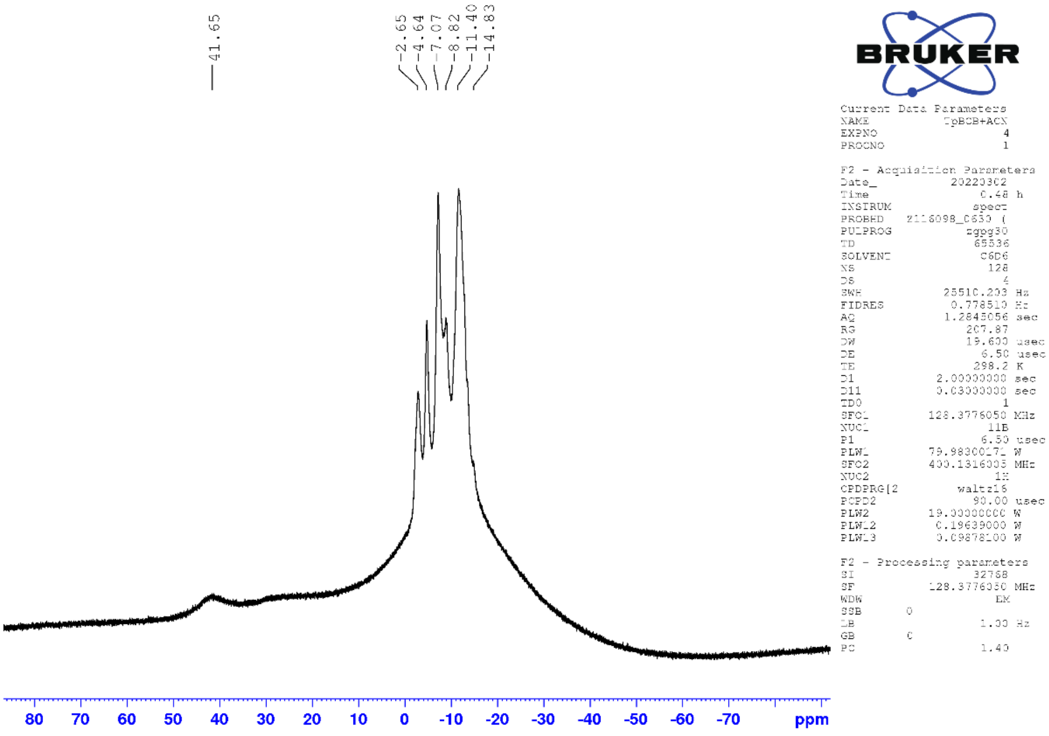

Figure S30. $^{11}B\{^1H\}$ NMR spectrum of **6** in C_6D_6

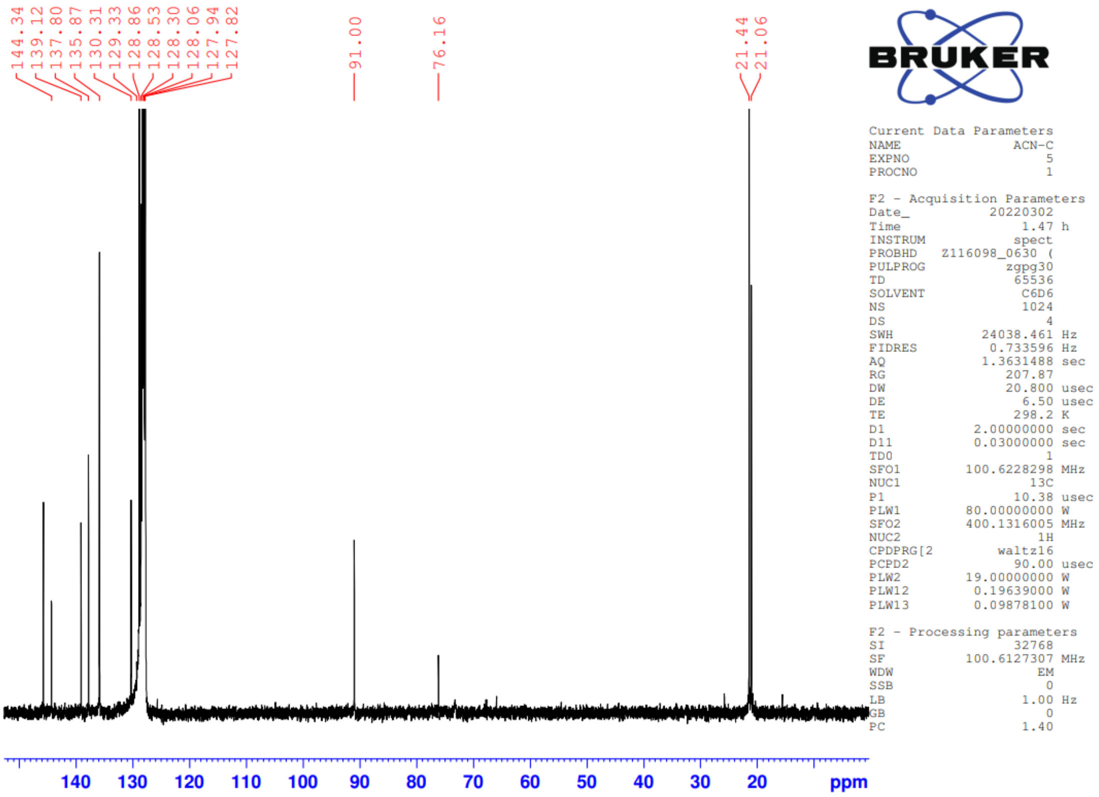

Figure S31 $^{13}C\{^{1}H\}$ NMR spectrum of **6** in C_6D_6

Crystal structures

Crystal data were collected on a Bruker D8 VENTURE diffractometer with graphite monochromated Mo Kα radiation (λ = 0.71073 Å) and Cu Kα (λ = 1.54178). Data reduction, scaling and absorption corrections were performed using SAINT (Bruker, V8.38A, 2013). The structure was solved with the XT structure solution program using the Intrinsic Phasing solution method[3] and by using Olex2 as the graphical interface. The model was refined with the ShelXL program[4] using Least Squares minimization. All non-hydrogen atoms were refined anisotropically. Hydrogen atoms were included in structure factor calculations. All hydrogen atoms were assigned to idealized geometric positions.

Crystallographic data have been deposited with the Cambridge Crystallographic Data as supplementary publication nos. 2206731 (**1**), 2206732 (**2**), and 2206733 (**3**) 2206734 (**4**), 2206735 (**5**), 2206736 (**6**). These data can be obtained free of charge from The Cambridge Crystallographic Data Centre via Data https://www.ccdc.cam.ac.uk

Table S1 Crystal data and structure refinement for 1.

Identification code	2206731
Empirical formula	$C_{26}H_{35}B_{11}$
Formula weight	466.45
Temperature/K	100.0
Crystal system	monoclinic
Space group	C2/c
a/Å	17.3102(15)
b/Å	12.1000(10)
c/Å	14.3004(10)
α/°	90
β/°	112.053(4)
γ/°	90
Volume/Å^3	2776.1(4)
Z	4
ρ_{calc}g/cm^3	1.116
μ/mm^{-1}	0.057
F(000)	984.0
Crystal size/mm^3	0.2 × 0.15 × 0.1
Radiation	MoKα (λ = 0.71073)
2Θ range for data collection/°	4.622 to 55.072
Index ranges	-22 ≤ h ≤ 22, -15 ≤ k ≤ 15, -18 ≤ l ≤ 18
Reflections collected	46603
Independent reflections	3194 [R_{int} = 0.0473, R_{sigma} = 0.0179]
Data/restraints/parameters	3194/0/172
Goodness-of-fit on F^2	1.084
Final R indexes [I>=2σ (I)]	R_1 = 0.0498, wR_2 = 0.1298
Final R indexes [all data]	R_1 = 0.0567, wR_2 = 0.1368
Largest diff. peak/hole / e Å^{-3}	0.36/-0.29

Table S2 Crystal data and structure refinement for 2.

Identification code	2206732
Empirical formula	C32H50B11OP
Formula weight	600.60
Temperature/K	99.99
Crystal system	monoclinic
Space group	P21/n
a/Å	10.5182(3)
b/Å	19.9773(8)
c/Å	16.5265(7)
α/°	90
β/°	91.520(2)
γ/°	90
Volume/Å3	3471.4(2)
Z	4
ρcalcg/cm3	1.149
μ/mm-1	0.105
F(000)	1280.0
Crystal size/mm3	0.13 × 0.12 × 0.11
Radiation	MoKα (λ = 0.71073)
2Θ range for data collection/°	4.378 to 55.04
Index ranges	-13 ≤ h ≤ 13, -25 ≤ k ≤ 25, -21 ≤ l ≤ 21
Reflections collected	110002
Independent reflections	7981 [Rint = 0.0993, Rsigma = 0.0382]
Data/restraints/parameters	7981/0/415
Goodness-of-fit on F2	1.066
Final R indexes [I>=2σ (I)]	R1 = 0.0395, wR2 = 0.0974
Final R indexes [all data]	R1 = 0.0598, wR2 = 0.1122
Largest diff. peak/hole / e Å-3	0.34/-0.33

Table S3 Crystal data and structure refinement for 3.

Identification code	2206733
Empirical formula	$C_{33}H_{41}B_{11}O$
Formula weight	572.57
Temperature/K	101.27
Crystal system	monoclinic
Space group	$P2_1$
a/Å	12.1994(10)
b/Å	11.1414(10)
c/Å	12.4485(10)
α/°	90
β/°	96.983(3)
γ/°	90
Volume/Å^3	1679.4(2)
Z	2
ρ_{calc}g/cm^3	1.132
μ/mm^{-1}	0.061
F(000)	604.0
Crystal size/mm^3	0.1 × 0.1 × 0.1
Radiation	MoKα (λ = 0.71073)
2Θ range for data collection/°	4.922 to 55.052
Index ranges	-15 ≤ h ≤ 15, -14 ≤ k ≤ 14, -16 ≤ l ≤ 15
Reflections collected	30381
Independent reflections	7589 [R_{int} = 0.1293, R_{sigma} = 0.1052]
Data/restraints/parameters	7589/1/412
Goodness-of-fit on F^2	1.043
Final R indexes [I>=2σ (I)]	R_1 = 0.0616, wR_2 = 0.1409
Final R indexes [all data]	R_1 = 0.0829, wR_2 = 0.1509
Largest diff. peak/hole / e Å^{-3}	0.26/-0.33

Table S4 Crystal data and structure refinement for 4.

Identification code	2206734
Empirical formula	$C_{46}H_{53}B_{11}O$
Formula weight	740.79
Temperature/K	99.96
Crystal system	triclinic
Space group	P-1
a/Å	9.1712(7)
b/Å	15.5443(11)
c/Å	15.5443(11)
α/°	86.78
β/°	77.906(2)
γ/°	77.905(2)
Volume/Å^3	2118.5(3)
Z	2
ρ_{calc}g/cm^3	1.161
μ/mm^{-1}	0.063
F(000)	784.0
Crystal size/mm^3	0.12 × 0.12 × 0.12
Radiation	MoKα (λ = 0.71073)
2Θ range for data collection/°	4.638 to 55.258
Index ranges	-11 ≤ h ≤ 11, -20 ≤ k ≤ 20, -20 ≤ l ≤ 19
Reflections collected	32860
Independent reflections	9750 [R_{int} = 0.0481, R_{sigma} = 0.0472]
Data/restraints/parameters	9750/0/466
Goodness-of-fit on F^2	1.034
Final R indexes [I>=2σ (I)]	R_1 = 0.0492, wR_2 = 0.1189
Final R indexes [all data]	R_1 = 0.0671, wR_2 = 0.1329
Largest diff. peak/hole / e Å^{-3}	0.35/-0.26

Table S5 Crystal data and structure refinement for 5.

Identification code	2206735
Empirical formula	$C_{33}H_{40}B_{11}N$
Formula weight	569.57
Temperature/K	99.98
Crystal system	monoclinic
Space group	C2/c
a/Å	32.827(3)
b/Å	10.5175(8)
c/Å	19.8270(15)
α/°	90
β/°	97.737(3)
γ/°	90
Volume/Å^3	6783.1(9)
Z	8
ρcalcg/cm^3	1.115
μ/mm^{-1}	0.059
F(000)	2400.0
Crystal size/mm^3	0.1 × 0.1 × 0.1
Radiation	MoKα (λ = 0.71073)
2Θ range for data collection/°	4.49 to 54.924
Index ranges	-42 ≤ h ≤ 40, -13 ≤ k ≤ 13, -24 ≤ l ≤ 25
Reflections collected	47961
Independent reflections	7645 [R_{int} = 0.1218, R_{sigma} = 0.0833]
Data/restraints/parameters	7645/0/412
Goodness-of-fit on F^2	1.031
Final R indexes [I>=2σ (I)]	R_1 = 0.0729, wR_2 = 0.1882
Final R indexes [all data]	R_1 = 0.0938, wR_2 = 0.2068
Largest diff. peak/hole / e Å^{-3}	0.52/-0.41

Table S6 Crystal data and structure refinement for 6.

Identification code	2206736
Empirical formula	$C_{28}H_{38}B_{11}N$
Formula weight	507.50
Temperature/K	99.98
Crystal system	triclinic
Space group	P-1
a/Å	9.1308(4)
b/Å	11.8344(4)
c/Å	14.7020(5)
α/°	81.2150(10)
β/°	73.9600(10)
γ/°	89.3630(10)
Volume/Å^3	1508.09(10)
Z	2
ρ_{calc}g/cm^3	1.118
μ/mm^{-1}	0.058
F(000)	536.0
Crystal size/mm^3	0.12 × 0.12 × 0.12
Radiation	MoKα (λ = 0.71073)
2Θ range for data collection/°	4.644 to 49.638
Index ranges	-10 ≤ h ≤ 10, -13 ≤ k ≤ 13, -17 ≤ l ≤ 17
Reflections collected	20494
Independent reflections	5156 [R_{int} = 0.0316, R_{sigma} = 0.0287]
Data/restraints/parameters	5156/0/367
Goodness-of-fit on F^2	1.034
Final R indexes [I>=2σ (I)]	R_1 = 0.0516, wR_2 = 0.1285
Final R indexes [all data]	R_1 = 0.0574, wR_2 = 0.1339
Largest diff. peak/hole / e Å^{-3}	0.77/-0.38

Computational details

All calculations were performed with the Gaussian 09 program.[5] All structures were optimized at the B3LYP-D3/6-31G* level of theory.[6] Frequency calculations were performed to confirm that a transition state has only one imaginary frequency and a local minimum has no imaginary frequency. Intrinsic reaction coordinate (IRC) calculations[7] were also carried out to further confirm that transition states link the relevant local minima. Principle interacting orbital analysis was performed at the same level of theory.[8] Nucleus-Independent Chemical Shift (NICS) values were calculated at the B3LYP/6-311+G** level of theory[9] using the gauge-including atomic orbital method (GIAO)[10] implemented in the Gaussian 09 program. Both NICS values at ring critical point (NICS(0)[11]) and 1.0 Å above the critical point (NICS(1)[12]) were calculated. For the FIA and HIA calculations, geometries and final electronic energies were obtained at the BP86-D3/def2-SVP level of theory.[13] The FIA reaction enthalpies were calculated according to the scheme proposed by Krossing using the given G3 anchor points and isodesmic reactions.[14]

Table S7. Strain energies calculated for the parent borirane, benzoborirene and carborane-fused borirane based on the designed isodesmotic equations. The relative energies are given in kcal/mol.

Compounds	Homodesmotic Equations	ΔH
Borirane	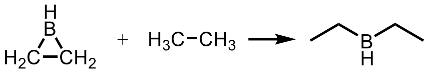	–40.2
Benzoborirene	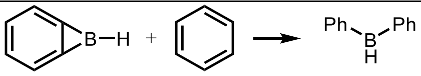	–49.6
Carborane-fused borirane	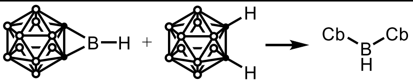	–56.8

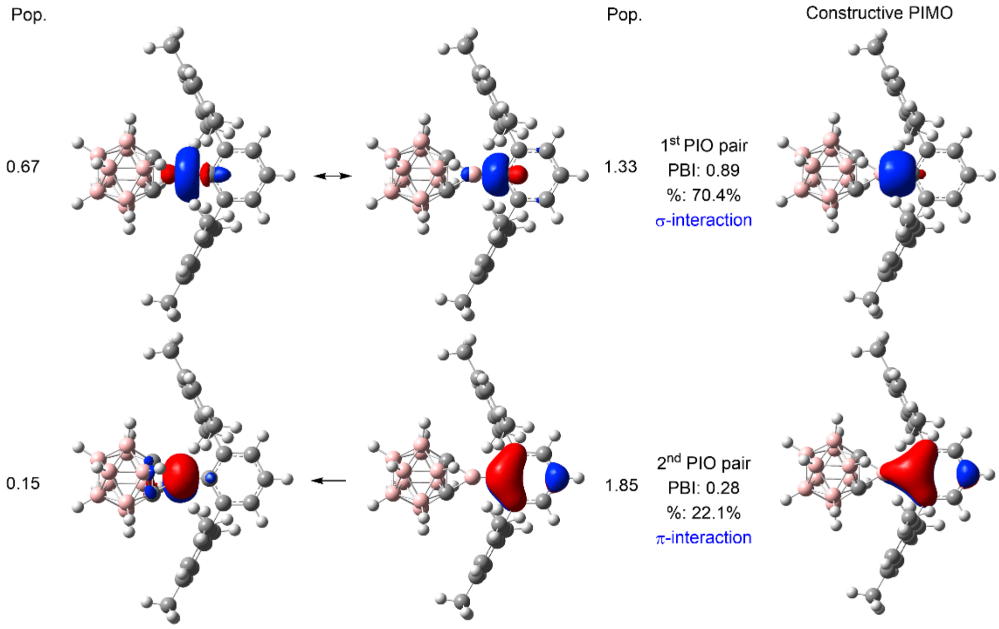

Figure S32. Results of PIO analysis on **1** considering the bonding interaction between BAr and the remaining part of the molecule. The isovalue of 0.05 $(e/Å)^{3/2}$ was used in the isosurface contour plots.

Table S8. Nucleus-independent chemical shift (NICS) of *B*-aryl ring in compound **1** and corresponding ring in TpH.

Coumpound	Position of Bqs	NICS(0)	NICS(1)
**1**	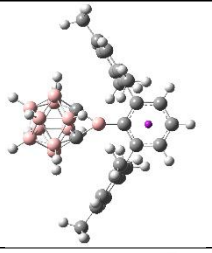	–5.92	–8.25
TpH	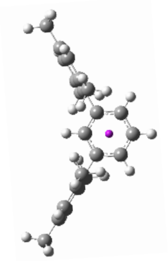	–7.68	–9.29

Note: The NICS value can be regarded as an indicator of the B-Ar p-π interaction since the interaction between the endocyclic π-electrons and the empty p-orbital on boron should result in perturbation in aromaticity. Usually, the aromatic character is reduced as a result of delocalization onto the strong electron deficient empty p-orbital.

Table S9. Calculated fluoride ion affinity energies of the parent borirane, benzoborirene, carborane-fused borirane **1'**, the experimentally characterized *B*-Tp carborane-fused borirane **1** and the *B*-bis(trimethylsilyl)amino carborane-fused borirane **V**. The FIAs are given refer to SbF_5.

	E/ Hartree	corr. Enthalpy/ Hartree	$\Delta E(H)$/kJ mol^{-1} (FIA Ref. to SbF_5)
SbF_5	-739.228	0.019	-
SbF_6^-	-839.160	0.022	0.000
**1'**	-355.999	0.170	-
**1'**-F^-	-455.947	0.174	38.686
The parent borirane	-103.921	0.065	-
The parent borirane-F^-	-203.802	0.069	-136.361
The parent benzoborirene	-256.245	0.094	-
The parent benzoborirene-F^-	-356.118	0.096	-154.477
**1**	-1284.476	0.592	-
**1**-F^-	-1384.406	0.595	-7.905
**V**	-1228.489	0.402	-
**V**-F^-	-1328.403	0.405	-45.533

Table S10. Calculated hydride ion affinity energies of the parent borirane, benzoborirene, carborane-fused borirane **1'**, the experimentally characterized *B*-Tp carborane-fused borirane **1** and the *B*-bis(trimethylsilyl)amino carborane-fused borirane **V**. The HIAs are given refer to $B(C_6F_5)_3$.

	E/ Hartree	corr. Enthalpy/ Hartree	ΔE(H)/kJ mol^{-1} (HIA Ref. to $B(C_6F_5)_3$)
$B(C_6F_5)_3$	-2206.674	0.180	-
$B(C_6F_5)_3$-H^-	-2207.376	0.188	0.000
**1'**	-355.999	0.170	-
**1'**-H^-	-356.716	0.179	34.911
The parent borirane	-103.921	0.065	-
The parent borirane-H^-	-104.575	0.074	-130.441
The parent benzoborirene	-256.245	0.094	-
The parent benzoborirene-H^-	-256.889	0.100	-150.859
**1**	-1284.476	0.592	-
**1**-H^-	-1285.175	0.600	-11.507
**V**	-1228.489	0.402	-
**V**-H^-	-1229.167	0.410	-63.704

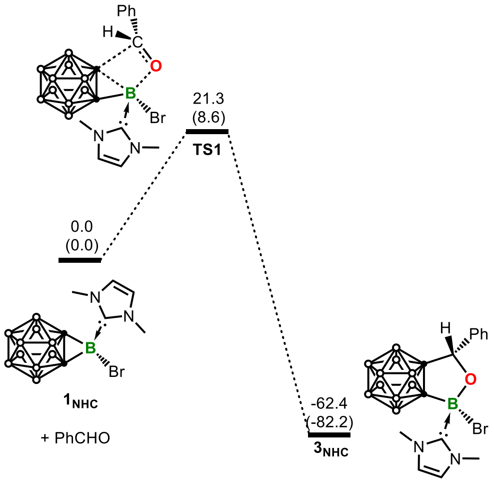

Figure S33. Energy profile calculated for the formation of **4$_{NHC}$**. The relative free energies (calculated at 298 K) and electronic energies (in parentheses) are given in kcal/mol.

Cartesian coordinates

Borirane

SCF Done: -104.008188

C	0.77682700	-0.35276900	0.00000100
B	0.00000200	0.97055400	-0.00000300
C	-0.77682800	-0.35276600	0.00000100
H	0.00000500	2.15654500	-0.00000200
H	1.28230100	-0.69402400	0.90460400
H	1.28229800	-0.69403200	-0.90460100
H	-1.28230400	-0.69401900	0.90460400
H	-1.28230100	-0.69402700	-0.90460100

Ethane

SCF Done: -79.832223

C	0.00000000	0.00000000	0.76565000
H	-0.51044400	0.88462000	1.16394900
H	1.02132500	-0.00025300	1.16394900
H	-0.51088100	-0.88436700	1.16394900
C	0.00000000	0.00000000	-0.76565000
H	0.51088100	-0.88436700	-1.16394900
H	-1.02132500	-0.00025300	-1.16394900
H	0.51044400	0.88462000	-1.16394900

Diethyl borane

SCF Done: -183.908445

B	0.00000100	0.31260000	0.00001200
H	-0.00000200	1.51824200	-0.00010000
C	-1.35069400	-0.44502900	0.25470400
H	-1.33631000	-0.61112400	1.35102400
H	-1.33426100	-1.45996000	-0.16984500
C	1.35068400	-0.44508000	-0.25459000
H	1.33624300	-0.61141500	-1.35087300
H	1.33426600	-1.45992100	0.17017700
C	2.64684300	0.29039400	0.12292800
H	3.54155300	-0.26336300	-0.18617400
H	2.68559400	1.28146600	-0.34369700
H	2.71292600	0.44057600	1.20726000
C	-2.64683100	0.29037300	-0.12303500
H	-3.54155900	-0.26331600	0.18614000
H	-2.68560100	1.28154300	0.34337900
H	-2.71285900	0.44032500	-1.20740100

Benzoborirene

SCF Done: -256.441756

B	0.00000000	0.00000000	2.23357700
H	0.00000000	0.00000000	3.41912300
C	0.00000000	0.71332500	-1.43608600
C	0.00000000	-0.71332500	-1.43608600
C	0.00000000	-1.44870600	-0.26323100
C	0.00000000	-0.70210900	0.93052600
C	0.00000000	0.70210900	0.93052600
C	0.00000000	1.44870600	-0.26323100
H	0.00000000	1.22633300	-2.39522400
H	0.00000000	-1.22633300	-2.39522400
H	0.00000000	-2.53629900	-0.28553400
H	0.00000000	2.53629900	-0.28553400

Benzene

SCF Done: -232.253741

C	1.38463100	0.18348300	-0.00000100
C	0.85113500	-1.10725800	0.00002800
C	-0.53336100	-1.29070900	-0.00002100
C	-1.38463800	-0.18343100	0.00000500
C	-0.85117700	1.10722600	0.00001800
C	0.53341000	1.29068900	-0.00002200
H	2.46215500	0.32619900	-0.00000600
H	1.51358000	-1.96902100	0.00002200
H	-0.94842600	-2.29529000	-0.00003000
H	-2.46214500	-0.32627900	-0.00000600

H	-1.51351900	1.96906900	0.00001700
H	0.94835400	2.29531900	-0.00004000

Bisphenyl borane

SCF Done: -488.778414

B	-0.00001200	-1.22208200	0.00021400
H	-0.00001700	-2.42490600	-0.00002400
C	-1.38219900	-0.51867900	-0.04288800
C	-1.57528400	0.80013900	-0.51077000
C	-2.52741000	-1.22643700	0.38380600
C	-2.83931700	1.38629300	-0.53460100
H	-0.72351000	1.36164800	-0.88382800
C	-3.79112000	-0.63932900	0.38767100
H	-2.41337300	-2.25330500	0.72332000
C	-3.94800500	0.67037700	-0.07412200
H	-2.96403500	2.39842600	-0.91100500
H	-4.65433700	-1.20108500	0.73526400
H	-4.93398700	1.12839500	-0.08609100
C	1.38219400	-0.51869900	0.04306500
C	2.52732600	-1.22644800	-0.38384500
C	1.57536500	0.80010000	0.51096800
C	3.79103900	-0.63934700	-0.38790600
H	2.41322700	-2.25330500	-0.72337400
C	2.83940700	1.38624400	0.53460900
H	0.72365900	1.36159700	0.88419800
C	3.94801200	0.67034100	0.07391000
H	4.65419400	-1.20109500	-0.73566700
H	2.96419600	2.39836000	0.91103600
H	4.93399900	1.12835000	0.08573000

1'

SCF Done: -356.298132

C	-1.11980900	0.00001200	0.82668300
B	-2.41695700	-0.00003100	0.00000000
B	1.59875900	0.00010700	0.90537800
H	2.60668800	0.00017900	1.53478500
B	0.17358200	0.90055500	1.46701400
H	0.03195200	1.50301600	2.47761700
B	0.17363900	-0.90036200	1.46713100
H	0.03205000	-1.50270000	2.47781200
B	1.07076500	-1.45813000	0.00009500
H	1.66560400	-2.48615600	0.00016300
B	-0.67695700	-1.45982500	0.00009400
H	-1.47540100	-2.34080800	0.00015300
C	-1.11980800	-0.00009900	-0.82668300
B	1.59875900	-0.00001000	-0.90537800
H	2.60668800	-0.00001800	-1.53478500
B	0.17364000	-0.90055300	-1.46701500
H	0.03205100	-1.50302200	-2.47761800
B	0.17358000	0.90036400	-1.46713000
H	0.03195100	1.50269400	-2.47781100
B	1.07066900	1.45819200	-0.00009500
H	1.66543400	2.48626100	-0.00016300
B	-0.67705300	1.45976800	-0.00009400
H	-1.47556000	2.34069700	-0.00015300
H	-3.59589500	0.00000100	0.00000000

Carborane

SCF Done: -332.130191

C	0.29950000	0.75578100	-1.27664700
B	0.32903100	0.83036200	1.46436800
H	0.56644400	1.43020400	2.46233200
B	1.36214600	1.01131500	0.02616100
H	2.28292600	1.73910500	-0.14186000
B	-0.29950000	1.66933100	0.02592800
H	-0.47230300	2.83021800	-0.14208300
B	-1.35334900	0.53639800	0.91242300
H	-2.31042200	0.91589000	1.50514200
B	-1.35639000	0.53719800	-0.85404000

H	-2.17179700	0.86031300	-1.64831800
C	-0.29950000	-0.75578100	-1.27664700
B	-0.32903100	-0.83036200	1.46436800
H	-0.56644400	-1.43020400	2.46233200
B	-1.36214600	-1.01131500	0.02616100
H	-2.28292600	-1.73910500	-0.14186000
B	0.29950000	-1.66933100	0.02592800
H	0.47230300	-2.83021800	-0.14208300
B	1.35334900	-0.53639800	0.91242300
H	2.31042200	-0.91589000	1.50514200
B	1.35639000	-0.53719800	-0.85404000
H	2.17179700	-0.86031300	-1.64831800
H	0.47478700	1.19941700	-2.24952800
H	-0.47478700	-1.19941700	-2.24952800

Biscarboranyl borane

SCF Done: -688.523443

B	0.00000000	-1.08234000	-0.00000200
C	-1.39554100	-0.38602900	-0.00290600
B	-1.76390500	1.20706600	0.51586400
B	-1.90293900	0.73892000	-1.19459100
B	-2.43484500	-0.97013400	-1.26880700
C	-2.65384400	-1.40580000	0.36665500
B	-2.18717900	-0.21188200	1.51655400
B	-3.19990700	1.67684400	-0.41517100
H	-0.86842800	1.88659100	0.87730400
B	-3.36665000	1.08685700	1.27105500
H	-1.09934800	1.09437300	-1.98770700
B	-3.60290100	0.32828400	-1.53074700
H	-1.98765400	-1.77995800	-2.00802100
B	-4.05440700	-1.07499400	-0.52592500
B	-3.89933200	-0.61590100	1.18931800
H	-2.36274000	-2.41940600	0.61586400
H	-1.60369800	-0.56636200	2.48431100
H	-3.38516900	2.81318200	-0.70697100
B	-4.52252200	0.55649400	0.00046700
H	-3.67237900	1.78741600	2.17975100
H	-4.07052600	0.49425100	-2.60966100
H	-4.48554900	-1.22106900	2.02311700
H	-5.66326500	0.88787900	0.00915300
C	1.39554300	-0.38603400	0.00299200
B	1.76379700	1.20731900	-0.51505500
B	1.90317200	0.73831800	1.19514200
B	2.43509700	-0.97077000	1.26839700
C	2.65377700	-1.40561600	-0.36732700
B	2.18688500	-0.21112500	-1.51653700
B	3.19998100	1.67663800	0.41593400
H	0.86823900	1.88701700	-0.87598200
B	3.36639600	1.08749600	-1.27061900
H	1.09974400	1.09336900	1.98859700
B	3.60320100	0.32752000	1.53075600
H	1.98805400	-1.78096600	2.00729300
B	4.05451400	-1.07525200	0.52514300
B	3.89910200	-0.61530100	-1.18983800
H	2.36262800	-2.41909900	-0.61698600
H	1.60321600	-0.56512400	-2.48435700
H	3.38529500	2.81283100	0.70826600
B	4.52251900	0.55650100	-0.00052400
H	3.67194400	1.78851000	-2.17902400
H	4.07103800	0.49294800	2.60966100
H	4.48515900	-1.22005000	-2.02405400
H	5.66325900	0.88789500	-0.00926800
H	4.74046500	-1.99063000	0.83624600
H	-4.74029200	-1.99021900	-0.83762000
H	0.00000100	-2.27529600	-0.00047300

1

SCF Done: -1285.454992

C	-0.00000500	1.46104200	0.00000100

C	2.52109400	1.41562500	0.04187200
C	3.16308300	1.12652800	-1.17698200
C	-0.80168000	-1.36510700	-0.17851000
C	1.22967200	2.16883500	0.03238900
C	3.04843400	0.93764800	1.25612600
C	4.86441800	-0.14249700	0.03146000
C	4.32776100	0.35367200	-1.15966500
H	4.82099600	0.12313700	-2.10174000
C	4.21564500	0.17004500	1.22957400
H	4.62034900	-0.20545100	2.16727800
C	1.21348000	3.56524700	0.02953800
H	2.15398500	4.10856200	0.05011200
C	2.59191700	1.61921400	-2.48770400
H	1.57905700	1.23165700	-2.65217000
H	3.21243900	1.30395200	-3.33160800
H	2.51561400	2.71264800	-2.51069700
C	2.33999500	1.20260800	2.56467100
H	2.05493700	2.25531900	2.66873900
H	2.96961000	0.93060900	3.41698400
H	1.41600500	0.61447600	2.63635500
C	-0.00001300	4.25600200	-0.00000400
H	-0.00001600	5.34311300	-0.00000400
B	0.00000100	-0.05260300	0.00000000
C	6.08779300	-1.02788600	0.02224100
H	5.80049800	-2.08619400	-0.03375000
H	6.68391900	-0.90010300	0.93225500
H	6.73077900	-0.81774100	-0.83905600
B	-0.88343100	-4.08349600	-0.19977500
H	-1.49712900	-5.09280500	-0.33869500
B	-1.62935400	-2.65488100	0.55460700
H	-2.74763300	-2.51127000	0.91913100
B	-1.23174200	-2.65200400	-1.19836800
H	-2.08542700	-2.50965300	-2.00875200
B	0.32067000	-3.55726700	-1.41965500
H	0.54565700	-4.15547500	-2.42198700
B	0.32295500	-1.81541800	-1.42078100
H	0.51740200	-1.02069600	-2.28240500
C	-2.52110400	1.41561100	-0.04187400
C	-3.16308300	1.12651200	1.17698500
C	0.80169400	-1.36510000	0.17850500
C	-1.22968600	2.16882800	-0.03239700
C	-3.04845300	0.93763500	-1.25612400
C	-4.86442200	-0.14251800	-0.03144500
C	-4.32775900	0.35365300	1.15967700
H	-4.82098700	0.12311900	2.10175400
C	-4.21566000	0.17002800	-1.22956400
H	-4.62037200	-0.20546800	-2.16726400
C	-1.21350200	3.56524000	-0.02954600
H	-2.15400900	4.10855000	-0.05011800
C	-2.59191300	1.61920900	2.48770100
H	-1.57905800	1.23164000	2.65217300
H	-3.21244100	1.30396700	3.33160900
H	-2.51559400	2.71264200	2.51067900
C	-2.34002900	1.20260400	-2.56467600
H	-2.05498800	2.25531800	-2.66875000
H	-2.96964500	0.93059100	-3.41698400
H	-1.41602900	0.61448700	-2.63636500
C	-6.08779500	-1.02791100	-0.02221700
H	-5.80049600	-2.08621800	0.03375500
H	-6.68393600	-0.90011700	-0.93222000
H	-6.73076700	-0.81777900	0.83909300
B	0.88346900	-4.08348800	0.19976400
H	1.49717700	-5.09279200	0.33868100
B	1.62937900	-2.65486400	-0.55461500
H	2.74765700	-2.51124300	-0.91913800
B	1.23176700	-2.65199600	1.19836100
H	2.08545100	-2.50963900	2.00874400
B	-0.32063700	-3.55727300	1.41964500
H	-0.54561800	-4.15548600	2.42197600

B	-0.32293700	-1.81542400	1.42077600
H	-0.51739100	-1.02070700	2.28240100

TpH

SCF Done: -930.308426

C	0.00000000	0.00000000	0.27326600
C	2.51141100	-0.01636000	0.20217200
C	3.16971000	1.19629700	-0.07767700
C	1.22186100	-0.00747500	0.95764700
C	3.06607700	-1.23763200	-0.22663000
C	4.94773200	-0.03357300	-1.21454100
C	4.37858300	1.16665500	-0.77994000
H	4.88813100	2.10467200	-0.99309900
C	4.27674400	-1.22544300	-0.92586800
H	4.70751300	-2.17053000	-1.25181600
C	1.20958400	-0.00759300	2.35914800
H	2.15340200	-0.01452600	2.89831800
C	2.57767200	2.51282800	0.37272400
H	1.57156800	2.65868200	-0.03904700
H	3.20034800	3.35606900	0.05813800
H	2.47296000	2.55378600	1.46329700
C	2.36590400	-2.54480300	0.06988600
H	2.21281100	-2.68107700	1.14694200
H	2.94164400	-3.39696000	-0.30438500
H	1.37081200	-2.57812900	-0.39016600
C	0.00000000	0.00000100	3.05468400
H	0.00000000	0.00000100	4.14173600
C	6.23753500	-0.04121000	-2.00183300
H	6.04276200	0.00417700	-3.08183500
H	6.81663000	-0.95234800	-1.81527600
H	6.86761800	0.81856000	-1.74927300
C	-2.51141100	0.01636000	0.20217200
C	-3.16971000	-1.19629800	-0.07767600
C	-1.22186100	0.00747600	0.95764700
C	-3.06607700	1.23763200	-0.22663100
C	-4.94773200	0.03357200	-1.21454100
C	-4.37858300	-1.16665500	-0.77993900
H	-4.88813000	-2.10467300	-0.99309800
C	-4.27674400	1.22544200	-0.92586800
H	-4.70751300	2.17052900	-1.25181700
C	-1.20958400	0.00759500	2.35914800
H	-2.15340200	0.01452700	2.89831800
C	-2.57767200	-2.51282800	0.37272500
H	-1.57156700	-2.65868200	-0.03904500
H	-3.20034800	-3.35606900	0.05814000
H	-2.47296000	-2.55378600	1.46329800
C	-2.36590500	2.54480300	0.06988500
H	-2.21281100	2.68107700	1.14694100
H	-2.94164400	3.39696000	-0.30438700
H	-1.37081300	2.57812900	-0.39016700
C	-6.23753500	0.04120900	-2.00183300
H	-6.04276100	-0.00417100	-3.08183500
H	-6.81663400	0.95234400	-1.81527000
H	-6.86761400	-0.81856500	-1.74927900
H	0.00000000	0.00000000	-0.81408800

1NHC

SCF Done: -3232.368927

Br	-1.26830200	2.23884700	-0.00159000
N	-2.24261900	-1.04398700	-1.07980900
N	-2.24368700	-1.04128500	1.08076600
C	1.01248100	0.05968900	-0.78853700
C	1.01236500	0.06074800	0.78866800
C	-1.59017300	-0.57344800	0.00021500
C	-3.31063700	-1.82828600	-0.67957800
H	-3.96643800	-2.30823100	-1.38840800
C	-3.31129900	-1.82659500	0.68144300
H	-3.96781600	-2.30474500	1.39082300
B	1.92118500	1.33278400	-0.00072500

H	1.46295400	2.41789900	-0.00149300
B	2.51961900	0.54757000	-1.45446600
H	2.55887100	1.15346400	-2.47405800
B	2.02202200	-1.17155800	-1.45236700
H	1.72026300	-1.71128100	-2.46813300
B	1.09988500	-1.49464700	0.00116100
H	0.13336400	-2.18055600	0.00162100
B	2.51936400	0.54957500	1.45416000
H	2.55844500	1.15689500	2.47291000
B	3.56719300	0.81674000	-0.00026100
H	4.43456900	1.63058000	-0.00077600
B	3.65856000	-0.71828200	-0.90376800
H	4.62023300	-0.99851300	-1.54642800
B	2.75846700	-1.96107100	0.00158000
H	3.03822900	-3.11778800	0.00238600
B	2.02180400	-1.16959000	1.45436800
H	1.71999300	-1.70795000	2.47083500
B	3.65843700	-0.71705200	0.90536800
H	4.62005000	-0.99637500	1.54851000
B	-0.36219600	0.43016700	-0.00035900
C	-1.86135200	-0.71244600	2.45473800
H	-1.79846500	0.37431200	2.54775700
H	-2.62237100	-1.10158200	3.13223300
H	-0.89034700	-1.15568200	2.68602100
C	-1.85897000	-0.71847000	-2.45420500
H	-2.61821100	-1.11117700	-3.13163200
H	-1.79806200	0.36812600	-2.55041400
H	-0.88675600	-1.16047700	-2.68271700

TS1

SCF Done: -3577.936833

Br	-0.03136900	-1.70238500	-1.30787900
N	-2.85069700	-1.36664700	1.32175100
N	-3.50623700	-0.85609300	-0.68074600
C	0.58218100	1.28880500	0.77773100
C	-0.64906600	1.12583300	-0.31971400
C	-2.43177300	-0.87699400	0.14031600
C	-4.20448400	-1.64260700	1.25103900
H	-4.74520600	-2.03661900	2.09676000
C	-4.61594400	-1.32563500	-0.00581100
H	-5.58414400	-1.39263300	-0.47598500
B	0.88578400	1.54279000	-0.89495800
H	1.52429200	0.74663900	-1.49649100
B	1.51336600	2.66019800	0.29152100
H	2.68224900	2.66697500	0.50936700
B	0.34183500	2.78641500	1.59905900
H	0.68694400	2.87247600	2.73536600
B	-1.01734000	1.77304900	1.20330300
H	-1.63723400	1.12269600	1.98246600
B	-0.61439700	2.25172300	-1.60297500
H	-0.96749100	1.88974600	-2.68197000
B	0.77408800	3.28749500	-1.20826800
H	1.42714300	3.78647000	-2.07026300
B	0.43157000	4.08069200	0.36006600
H	0.84965800	5.16453900	0.62583100
B	-1.15962400	3.51894900	0.94049900
H	-1.89219400	4.17485400	1.61342000
B	-1.80016000	2.39719000	-0.27559200
H	-2.94741400	2.12349400	-0.45804600
B	-0.89712400	3.81957500	-0.81044700
H	-1.43786400	4.71053500	-1.38911200
B	-1.01212300	-0.37952100	-0.37133700
C	-3.46396800	-0.43127100	-2.08488100
H	-2.76949700	-1.07130700	-2.63388600
H	-4.46579500	-0.52307200	-2.50487200
H	-3.13343500	0.60768500	-2.14355400
C	-2.03959300	-1.54652900	2.53426800
H	-2.38204000	-2.45393800	3.03590500
H	-0.98410700	-1.65908600	2.26974800

H	-2.17277500	-0.68125100	3.18692800
C	1.75326900	-1.15155900	1.80825600
O	0.92490700	-2.05784600	1.73010600
H	1.68437500	-0.38281100	2.59454500
C	2.93150600	-1.05959300	0.92553000
C	3.17952200	-2.06216900	-0.02419400
C	3.80605400	0.02881000	1.03591900
C	4.28117400	-1.96229800	-0.86814500
H	2.49866500	-2.90529800	-0.07973100
C	4.90796900	0.12841100	0.19012700
H	3.59758100	0.80830400	1.76260300
C	5.14407700	-0.86576500	-0.76348600
H	4.47457700	-2.73664800	-1.60578200
H	5.57446100	0.98272700	0.26526600
H	6.00153400	-0.78645700	-1.42669900

4NHC

SCF Done: -3578.081476

Br	-0.85967800	-1.52484100	-1.90361900
N	-2.63438400	-1.27877800	1.73661000
N	-3.49111700	-0.38659700	-0.03553500
C	1.03625100	1.11582100	0.56891500
C	-0.38487600	1.04421000	-0.24154600
C	-2.31310200	-0.72846200	0.53989700
C	-4.00763500	-1.28352000	1.90601000
H	-4.47184500	-1.67492200	2.79717400
C	-4.54461000	-0.72472600	0.79153400
H	-5.56991800	-0.53772500	0.51448700
B	1.07717200	1.23465200	-1.14201300
H	1.47521500	0.30740600	-1.74839900
B	2.11566400	2.24275900	-0.11391800
H	3.27495800	2.00204400	-0.08468100
B	1.23819100	2.59493900	1.39508100
H	1.80941000	2.57594500	2.43655200
B	-0.34776100	1.81357800	1.30268400
H	-0.82842600	1.22269800	2.21195800
B	-0.38980800	2.13788700	-1.54148800
H	-0.98160100	1.84196900	-2.52654600
B	1.19896700	2.95773000	-1.47041300
H	1.74554000	3.33836600	-2.45498000
B	1.30047000	3.81210100	0.10134900
H	1.92875900	4.81344100	0.23202900
B	-0.22845600	3.53483000	0.98968600
H	-0.69928600	4.31383700	1.75537900
B	-1.26077800	2.49926700	-0.03549000
H	-2.44646600	2.44194000	0.01283700
B	-0.25196200	3.75121500	-0.78874700
H	-0.73966600	4.70965200	-1.29717900
B	-0.81732600	-0.52383700	-0.06190500
C	-3.67675500	0.15193600	-1.38688400
H	-3.69142900	-0.66787800	-2.10679100
H	-4.61915700	0.70243900	-1.40472200
H	-2.86168300	0.82451900	-1.63690000
C	-1.70051200	-1.78718000	2.74942600
H	-2.29186400	-2.23558100	3.54959800
H	-1.03714500	-2.52618100	2.30579100
H	-1.10111500	-0.96712800	3.14610000
C	1.30317600	-0.29866000	1.14268100
O	0.17388100	-1.09064800	0.81040700
H	1.33529200	-0.19204700	2.23576200
C	2.60487200	-0.92778800	0.68779500
C	2.63453700	-1.85640200	-0.35637000
C	3.79628900	-0.56227200	1.32392000
C	3.85137100	-2.40578400	-0.76309200
H	1.70867600	-2.13099800	-0.84977800
C	5.01158900	-1.10873200	0.91279500
H	3.77310800	0.15771600	2.13939100
C	5.04054700	-2.03458700	-0.13263800
H	3.86806200	-3.12509300	-1.57757300

H	5.93227600	-0.81581800	1.41021000
H	5.98578300	-2.46508300	-0.45261400

PhCHO

SCF Done: -345.581538

O	-2.84490100	-0.39706100	-0.00048000
C	-1.99218800	0.47050500	0.00025200
H	-2.27693100	1.54724700	0.00077800
C	-0.53355100	0.21768900	0.00019600
C	0.36354700	1.29377300	0.00007800
C	-0.04813100	-1.09915400	0.00024700
C	1.73789100	1.05872900	-0.00017900
H	-0.01950400	2.31237000	0.00021700
C	1.32283300	-1.33272300	0.00009800
H	-0.76512700	-1.91459400	0.00035200
C	2.21590000	-0.25403500	-0.00017600
H	2.43420000	1.89260700	-0.00036100
H	1.70189300	-2.35095000	0.00014000
H	3.28687800	-0.43889000	-0.00038700

Ph2CO

SCF Done: -576.651230

O	-0.00013400	2.31657500	-0.00052400
C	0.00003900	1.09020800	-0.00032600
C	1.30088000	0.34378500	-0.02734300
C	1.44413800	-0.91523300	-0.63060300
C	2.43220800	0.97902500	0.50881600
C	2.69440100	-1.53237300	-0.68287200
H	0.58327600	-1.40006600	-1.07987200
C	3.67483900	0.35364200	0.47364400
H	2.31228800	1.96471400	0.94691300
C	3.80828800	-0.90444600	-0.12272600
H	2.79834400	-2.50147600	-1.16317900
H	4.54227500	0.84654700	0.90415300
H	4.77995500	-1.39017500	-0.15593800
C	-1.30086100	0.34359100	0.02732500
C	-1.44390800	-0.91556600	0.63011300
C	-2.43238300	0.97912600	-0.50814900
C	-2.69417000	-1.53279000	0.68238900
H	-0.58299500	-1.40071700	1.07899200
C	-3.67504000	0.35381900	-0.47273700
H	-2.31260000	1.96497200	-0.94595200
C	-3.80827900	-0.90459500	0.12301000
H	-2.79782000	-2.50222900	1.16210400
H	-4.54264200	0.84706100	-0.90255800
H	-4.77992100	-1.39038800	0.15628900

1$_{\mathbf{Ph}}$

SCF Done:-587.387365

C	-0.72819000	0.82430600	-0.00020600
B	0.58489000	0.00054100	-0.00001500
B	-3.45367700	0.90404700	-0.00016700
H	-4.46076500	1.53589400	-0.00023200
B	-2.02470600	1.46360800	0.89905800
H	-1.88591300	2.47579900	1.50092200
B	-2.02478900	1.46319000	-0.89966600
H	-1.88590900	2.47518100	-1.50185400
B	-2.92660800	-0.00049400	-1.45634400
H	-3.52430100	-0.00086200	-2.48340000
B	-1.18187200	-0.00015700	-1.46100000
H	-0.38779300	-0.00013700	-2.34422400
C	-0.72782100	-0.82395800	0.00013700
B	-3.45335100	-0.90458100	0.00024400
H	-4.46020200	-1.53680100	0.00048700
B	-2.02424800	-1.46355900	-0.89906600
H	-1.88515900	-2.47579000	-1.50080400
B	-2.02412900	-1.46321900	0.89964400
H	-1.88509100	-2.47515900	1.50188100
B	-2.92644400	0.00013100	1.45627900

H	-3.52399100	0.00018900	2.48343300
B	-1.18172200	0.00047100	1.46102200
H	-0.38785700	0.00086000	2.34442600
C	2.09565900	0.00036800	0.00005500
C	2.81295700	-1.21458500	0.00004700
C	2.81360300	1.21492700	-0.00000500
C	4.20463700	-1.21513700	-0.00000500
H	2.26734900	-2.15439600	0.00010000
C	4.20528200	1.21473900	-0.00004000
H	2.26851300	2.15504000	-0.00004300
C	4.89822200	-0.00038400	-0.00005200
H	4.75103700	-2.15387400	-0.00002700
H	4.75217800	2.15318600	-0.00008400
H	5.98509000	-0.00067200	-0.00011900

TS2

SCF Done:-932.979301

C	-2.20148700	-0.06888700	0.82625900
B	-1.13482000	0.66432000	-0.04720700
B	-4.54196800	-1.48057200	0.99019700
H	-5.42014200	-1.93814100	1.64925200
B	-2.86994700	-1.42513300	1.60788500
H	-2.47882300	-1.74500900	2.68098700
B	-3.81132100	0.08772000	1.39682900
H	-4.04688800	0.78740700	2.32549500
B	-4.82015900	-0.08637600	-0.09304200
H	-5.85970200	0.48087600	-0.19789300
B	-3.34307900	0.83049700	-0.14298300
H	-3.14434600	1.99326700	-0.26949400
C	-2.14263100	-0.25311000	-0.79895300
B	-4.47423700	-1.69002500	-0.80609600
H	-5.30597600	-2.29221800	-1.40634800
B	-3.70171400	-0.24755800	-1.50451900
H	-3.86306900	0.21962000	-2.58294800
B	-2.75932800	-1.76204700	-1.29462700
H	-2.29369500	-2.31498100	-2.23461300
B	-3.30031400	-2.53644200	0.24768500
H	-3.27943200	-3.71732300	0.38509200
B	-1.81113100	-1.64467200	0.20228500
H	-0.69253500	-2.00394200	0.29067400
O	1.11583600	-0.64408800	-0.28605800
C	2.16449000	-0.14701000	0.10074400
C	3.46222300	-0.83870400	0.07866200
C	3.55508300	-2.16050800	-0.38774500
C	4.60915200	-0.16923600	0.52919100
C	4.78799700	-2.80239500	-0.40110900
H	2.65166700	-2.65630100	-0.72931000
C	5.84396700	-0.81487400	0.51381100
H	4.52721600	0.85416300	0.88895500
C	5.93126300	-2.12992500	0.04924300
H	4.86564600	-3.82477900	-0.75961100
H	6.73416700	-0.29914100	0.86196300
H	6.89338500	-2.63498500	0.03787000
C	-0.24262600	1.90022300	-0.09580000
C	0.30007000	2.36407300	-1.30945100
C	0.08041000	2.58264700	1.09189900
C	1.13703000	3.47654800	-1.33512900
H	0.06677200	1.83742200	-2.23075100
C	0.91935700	3.69739400	1.06937200
H	-0.33297700	2.23300100	2.03447300
C	1.44714300	4.14376800	-0.14473000
H	1.54913900	3.82672700	-2.27753500
H	1.15925100	4.21763300	1.99266300
H	2.09996900	5.01256800	-0.16528500
H	2.17513300	0.88825500	0.49521900

Int1

SCF Done:-932.996319

C	1.36650000	-0.63218600	-0.68404500

```
B                   0.51745300    0.48684000    0.16459900
B                   3.38381200   -2.51078800   -0.92890100
H                   4.00236300   -3.26985500   -1.60602900
B                   1.63577000   -2.25990000   -1.19043000
H                   0.95197800   -2.74100700   -2.03680900
B                   2.79367900   -0.95810500   -1.58934300
H                   2.89480600   -0.56857500   -2.70637700
B                   4.10454500   -0.99146300   -0.34322100
H                   5.21226500   -0.64232500   -0.60103200
B                   2.84808500    0.19171100   -0.26302600
H                   2.89778300    1.35776800   -0.42787300
C                   1.65552100   -0.43511600    0.86824200
B                   3.72282400   -2.27340500    0.83138000
H                   4.58221400   -2.86512200    1.40429400
B                   3.33491200   -0.57654700    1.24243500
H                   3.81739400    0.08246900    2.10356900
B                   2.18242300   -1.88254500    1.64301700
H                   1.88073100   -2.09925600    2.77045900
B                   2.23259100   -3.09698900    0.29993800
H                   2.00282900   -4.24826900    0.49569500
B                   0.95483900   -1.94579200    0.39630300
H                  -0.18346800   -2.15111100    0.64915000
O                  -0.96758200    0.14326500    0.44710500
C                  -1.68851000   -0.47926400   -0.37017000
C                  -3.08678400   -0.70779700   -0.13701900
C                  -3.71925300   -0.22869300    1.03017300
C                  -3.81969200   -1.43130200   -1.09886600
C                  -5.07035600   -0.47481800    1.22343500
H                  -3.13587600    0.32590800    1.75769400
C                  -5.17311200   -1.67457000   -0.89507700
H                  -3.32131100   -1.79913700   -1.99202100
C                  -5.79441500   -1.19633600    0.26321600
H                  -5.56721200   -0.11092000    2.11729600
H                  -5.74373300   -2.23327600   -1.63003700
H                  -6.85212000   -1.38707200    0.42183400
C                   0.52467500    2.05952500    0.00362200
C                   0.82705800    2.89941300    1.08681100
C                   0.15353200    2.65653500   -1.21144700
C                   0.77186800    4.28803700    0.95908500
H                   1.12622400    2.45741800    2.03443300
C                   0.09862700    4.04401500   -1.34889300
H                  -0.07543300    2.02648300   -2.06987400
C                   0.40681000    4.86349500   -0.26023100
H                   1.01820000    4.92149600    1.80761000
H                  -0.17799300    4.48641100   -2.30275800
H                   0.36722000    5.94491100   -0.36329000
H                  -1.22569300   -0.85377600   -1.28954700
```

TS3

```
SCF Done:-932.982066
C                   0.87444700    1.16979400    0.92908300
B                   0.56146600   -0.68814800   -0.24181200
B                   2.41458200    3.48580400    0.57782400
H                   2.79726200    4.52942100    1.00653200
B                   0.75888700    2.90403400    0.90384900
H                  -0.07424900    3.44519700    1.55928300
B                   2.13343600    2.09036600    1.66541700
H                   2.25268800    2.07888000    2.84781400
B                   3.49191600    2.06537100    0.48002000
H                   4.63137900    2.07521600    0.82456500
B                   2.50758200    0.64813300    0.75949200
H                   2.81823700   -0.38155400    1.25167100
C                   1.33621800    0.64842300   -0.51876900
B                   2.92165500    2.84588500   -1.02506800
H                   3.66936000    3.43046400   -1.74491400
B                   2.95941900    1.07163400   -0.91305100
H                   3.62979800    0.32262300   -1.54632100
B                   1.57392900    1.89089000   -1.68688900
H                   1.30620300    1.67854300   -2.82493100
```

B	1.23852200	3.38929300	-0.76105200
H	0.75706700	4.33879200	-1.29479300
B	0.26915300	1.96644200	-0.48571200
H	-0.86553800	1.81023000	-0.80203200
O	-0.90597300	-0.67456500	-0.34893900
C	-1.69805100	-0.09565100	0.45953500
C	-3.09312400	0.02621500	0.17737400
C	-3.65652800	-0.47811500	-1.01610400
C	-3.90530100	0.67957500	1.12832600
C	-5.01655400	-0.33402400	-1.24198100
H	-3.01431400	-0.96892100	-1.73988600
C	-5.26607500	0.82550500	0.88830000
H	-3.45745700	1.07303400	2.03675100
C	-5.81811100	0.31751300	-0.29251700
H	-5.46060100	-0.71788000	-2.15507300
H	-5.89635300	1.33266200	1.61169000
H	-6.88217200	0.43228300	-0.47926100
C	1.10476300	-2.12945800	-0.06185000
C	2.40691600	-2.48487700	-0.46709200
C	0.28870300	-3.14358500	0.47991800
C	2.87232300	-3.79093000	-0.33410800
H	3.05866700	-1.72791000	-0.88845800
C	0.75321700	-4.44831000	0.62866300
H	-0.72560300	-2.90971600	0.79409300
C	2.04862500	-4.77418600	0.21971700
H	3.87917800	-4.04152300	-0.65639600
H	0.10916800	-5.20999400	1.06006500
H	2.41430100	-5.79163400	0.33155500
H	-1.24077200	0.32246000	1.36350400

3'

SCF Done:-933.116984

C	-0.97432800	-0.86045100	-0.47259100
B	1.21370400	0.33124500	-0.38868000
B	-1.64761000	-3.41144600	0.27394700
H	-2.47854500	-4.25425000	0.38330800
B	-2.04497300	-1.69955400	0.55234000
H	-3.09684600	-1.21006500	0.78770000
B	-1.67398600	-2.27378100	-1.09538500
H	-2.47994900	-2.16260600	-1.96009700
B	-0.37168700	-3.50274000	-0.98396300
H	-0.28996100	-4.39039600	-1.76964200
B	0.01395000	-1.86289900	-1.47003600
H	0.35307100	-1.40461000	-2.50889900
C	0.59395800	-1.08749700	-0.04394200
B	0.05915700	-3.67108400	0.74736000
H	0.44834900	-4.69949800	1.19839700
B	1.08588100	-2.69678900	-0.33057200
H	2.20807600	-2.89081600	-0.66170700
B	0.71636100	-2.11481600	1.30932800
H	1.58941400	-1.91858800	2.08736100
B	-0.97078200	-2.55617400	1.69892800
H	-1.31059100	-2.77469500	2.81626800
B	-0.58871600	-0.91394700	1.20218500
H	-0.60339200	0.10757100	1.79739100
O	0.24351900	1.14232000	-0.91175600
C	-1.06829600	0.57155000	-1.05421600
C	-2.12798900	1.44492900	-0.42242900
C	-1.80736300	2.41126700	0.53359500
C	-3.46319500	1.25852900	-0.79816500
C	-2.81695600	3.17995500	1.11516500
H	-0.77024400	2.56570000	0.81032900
C	-4.47115000	2.02207000	-0.21174600
H	-3.71375000	0.50881800	-1.54529400
C	-4.14907800	2.98526800	0.74745900
H	-2.56015200	3.93212400	1.85594100
H	-5.50529600	1.86827700	-0.50669800
H	-4.93335300	3.58421600	1.20198600
C	2.65399600	0.82397100	-0.17611900

C	3.64837900	-0.02531200	0.34654900
C	3.00568000	2.15204900	-0.49191200
C	4.94876100	0.43450100	0.54772200
H	3.40043900	-1.05233600	0.59684400
C	4.30285400	2.61532600	-0.29053700
H	2.24704900	2.81656900	-0.89688400
C	5.27533000	1.75511500	0.23019100
H	5.70546000	-0.23224600	0.95185400
H	4.56052700	3.64190100	-0.53667800
H	6.28849400	2.11582100	0.38834300
H	-1.26705900	0.48284100	-2.12717300

TS4

SCF Done:-932.967931

C	-1.03671300	-0.42630400	0.53632400
B	0.88758400	-0.38882300	-0.74109000
B	-3.09113300	-2.31244700	0.80184500
H	-4.11097000	-2.64065100	1.32228500
B	-1.83123300	-1.41200100	1.70488100
H	-1.88321700	-1.04545200	2.83512800
B	-2.75010100	-0.59662800	0.43719900
H	-3.43106700	0.34789900	0.68086800
B	-3.03173600	-1.76407100	-0.89778700
H	-3.98336700	-1.68247800	-1.60835300
B	-1.75875300	-0.56635200	-0.99768800
H	-1.73328100	0.35221600	-1.75139100
C	-0.30712300	-1.38521400	-0.58399800
B	-2.26402000	-3.31513500	-0.44098100
H	-2.69883500	-4.36065400	-0.81019900
B	-1.42928300	-2.22170500	-1.57132700
H	-1.17164600	-2.39729800	-2.71768400
B	-0.51050600	-3.05557900	-0.28305500
H	0.37542800	-3.79889100	-0.55345800
B	-1.54254900	-3.10769400	1.18091600
H	-1.42697600	-3.98999200	1.97147300
B	-0.28191000	-1.89586400	1.04889300
H	0.72684200	-1.86479500	1.65872700
O	0.73331100	0.63850800	-1.74963600
C	0.13303700	1.77571800	-1.79729600
C	-0.37879900	2.50697900	-0.71237200
C	-0.12886100	2.15337000	0.64739900
C	-1.32401800	3.53300200	-1.01390300
C	-0.83874400	2.81015700	1.66025100
H	0.72121500	1.54351100	0.90197400
C	-2.02524500	4.14687300	-0.00247200
H	-1.51545200	3.78958800	-2.05258200
C	-1.78625500	3.77127100	1.33970600
H	-0.64379900	2.55575700	2.69664000
H	-2.76055300	4.91191000	-0.22930500
H	-2.33882700	4.26430100	2.13475100
C	2.33870500	-0.37828600	-0.15812100
C	3.38305300	-0.41729200	-1.10589900
C	2.69179700	-0.26849500	1.19890600
C	4.72027300	-0.39579400	-0.71178000
H	3.14714500	-0.47880000	-2.16564400
C	4.02834000	-0.21911000	1.59383500
H	1.91560900	-0.21432800	1.95410400
C	5.04641500	-0.29437500	0.64162500
H	5.50544600	-0.44974300	-1.46106400
H	4.27373700	-0.12735200	2.64834400
H	6.08751100	-0.26885100	0.95198700
H	0.00159400	2.15251400	-2.81178800

3"

SCF Done:-933.059011

C	-1.33180800	-0.45629200	0.26446500
B	1.18295000	0.62143900	-0.29339700
B	-1.93489300	-3.12200500	-0.22130200
H	-2.73915900	-3.99695900	-0.18596500

B	-1.88529700	-1.88375100	1.04615900
H	-2.61713800	-1.77897900	1.96975700
B	-2.42756800	-1.44697600	-0.59148100
H	-3.52011200	-1.02153100	-0.76484800
B	-1.42479500	-2.35914500	-1.75641800
H	-1.84840100	-2.67307300	-2.82160900
B	-1.07612300	-0.67481200	-1.43527100
H	-1.19424200	0.24281200	-2.16247600
C	0.22649900	-0.66141200	-0.28049900
B	-0.27127800	-3.35446900	-0.82254600
H	0.13496300	-4.39565700	-1.22731300
B	0.26875600	-1.82101400	-1.55399100
H	1.06467300	-1.65359500	-2.41771400
B	0.79118000	-2.26063000	0.07596400
H	1.94200600	-2.41086400	0.29637000
B	-0.55203500	-3.05233900	0.91662100
H	-0.36191600	-3.85796600	1.76965600
B	-0.19983200	-1.35079200	1.23092300
H	0.20451000	-0.81699900	2.20895100
O	0.71963000	1.85949500	-0.65971700
C	-0.51516700	2.37395600	-0.91276400
C	-1.65447200	2.04213900	-0.27343800
C	-1.65523000	0.97427500	0.81334600
C	-2.89792800	2.69082400	-0.64399000
C	-2.93053900	0.96662600	1.62341600
H	-0.82432500	1.19982100	1.49827700
C	-4.03219800	2.49352700	0.06313700
H	-2.88864100	3.35020100	-1.50850700
C	-4.02254200	1.65436200	1.25265600
H	-2.92969200	0.36722200	2.52636000
H	-4.95557800	2.98494300	-0.22785200
H	-4.92167100	1.60696800	1.86134100
C	2.70710100	0.58329900	0.01439500
C	3.56581200	1.40352800	-0.74667300
C	3.27858900	-0.17915400	1.05126800
C	4.93786200	1.43301000	-0.50895200
H	3.14603800	2.01955600	-1.53711300
C	4.64765200	-0.13183200	1.31081800
H	2.64789400	-0.79935400	1.67823200
C	5.48169700	0.66429800	0.52335500
H	5.58239000	2.05824400	-1.12107900
H	5.06407000	-0.71953300	2.12439900
H	6.55101700	0.69010700	0.71649200
H	-0.48102500	3.14421400	-1.67716400

TS5

SCF Done:-1164.052604

C	2.27367300	-0.33168600	0.23797600
B	1.50452600	0.95506400	-0.19773100
B	4.07139100	-2.31327500	-0.34708700
H	4.75452400	-3.21806400	0.01313000
B	3.83730800	-0.87640500	0.67329200
H	4.25013600	-0.67907500	1.76819500
B	2.47787000	-2.01550300	0.39935600
H	1.97696300	-2.58815900	1.30847100
B	2.58806700	-2.60092200	-1.30764000
H	2.20535300	-3.68478200	-1.61246300
B	1.44422700	-1.36589400	-0.88689100
H	0.27049200	-1.39527700	-0.84569300
C	2.19743900	0.13607300	-1.32997500
B	3.98942300	-1.79650700	-2.07960500
H	4.61492700	-2.33990800	-2.93309500
B	2.34724900	-1.18028000	-2.40127200
H	1.75773700	-1.17852500	-3.43033100
B	3.70587800	-0.04165800	-2.12577500
H	4.02943900	0.73022500	-2.96624000
B	4.78377800	-0.75672500	-0.86245100
H	5.94832100	-0.51782100	-0.84577800
B	3.65504600	0.49525100	-0.43672300

H	3.82279700	1.62229800	-0.10844500
O	-1.11488500	0.60527900	-0.55984100
C	-1.96439800	-0.13682000	-0.06214200
C	-3.32596400	-0.21170000	-0.66610900
C	-4.45362000	-0.61717900	0.06553600
C	-3.47976800	0.19078600	-2.00334100
C	-5.71212800	-0.62777900	-0.53508800
H	-4.34764500	-0.90437100	1.10661800
C	-4.73366600	0.16416100	-2.60443200
H	-2.60084600	0.51519500	-2.55077500
C	-5.85232400	-0.24489700	-1.87053300
H	-6.58227300	-0.93281700	0.03931500
H	-4.84376700	0.46298100	-3.64311000
H	-6.83244100	-0.26145900	-2.33962800
C	-1.61559700	-0.97066400	1.12667000
C	-0.75906700	-0.42868000	2.09672500
C	-2.03239600	-2.30538700	1.24207600
C	-0.33572000	-1.20452200	3.17131300
H	-0.44003300	0.60253100	1.99689100
C	-1.58578700	-3.08826400	2.30657900
H	-2.67580900	-2.73662500	0.48145500
C	-0.74241700	-2.53834900	3.27391500
H	0.32103200	-0.77578000	3.92301300
H	-1.89183700	-4.12808000	2.37724400
H	-0.39768600	-3.14869600	4.10399200
C	1.04799600	2.33262200	0.26904700
C	0.49299000	3.25994000	-0.63174600
C	1.20749700	2.71070700	1.61555200
C	0.10387000	4.52462000	-0.19853100
H	0.35574400	2.97336700	-1.67009000
C	0.82489400	3.97827200	2.05072100
H	1.64530100	2.00536900	2.31821200
C	0.27057000	4.88435500	1.14259700
H	-0.32838300	5.23173800	-0.90118300
H	0.95767800	4.26132700	3.09132200
H	-0.03091700	5.87281100	1.47949700

Int2

SCF Done:-1164.073906

C	1.60084600	-0.65790800	0.14643900
B	0.84152200	0.65206300	-0.45553100
B	3.20058800	-2.90822400	-0.02466600
H	3.76228600	-3.81217400	0.50967900
B	3.07467200	-1.30540300	0.75582600
H	3.46120200	-1.00153300	1.83768300
B	1.62090300	-2.32673300	0.57154300
H	1.02260800	-2.70445900	1.52578000
B	1.74690400	-3.18257200	-1.01826400
H	1.24818900	-4.25086800	-1.18510500
B	0.74254900	-1.79437000	-0.85038000
H	-0.43844800	-1.76400000	-0.89049100
C	1.64389300	-0.42211400	-1.42413900
B	3.24900000	-2.65136000	-1.81468900
H	3.84535800	-3.37208800	-2.55145700
B	1.70144000	-1.90993900	-2.30824500
H	1.15607500	-2.00410600	-3.35934500
B	3.15472200	-0.89302600	-2.12213300
H	3.59900500	-0.30133500	-3.05020400
B	4.09874600	-1.53662300	-0.71731600
H	5.28294300	-1.42934700	-0.66786100
B	3.11983900	-0.12403000	-0.54753700
H	3.45126800	0.99485400	-0.38742800
O	-0.68257700	0.62078000	-0.63141000
C	-1.62750600	0.11270700	0.04792600
C	-2.92162000	0.02912300	-0.63257200
C	-4.13106300	-0.00052200	0.08822300
C	-2.94758600	0.00280300	-2.04165400
C	-5.34375700	-0.06049100	-0.59162300
H	-4.11551000	0.05038500	1.17143400

C	-4.16191900	-0.07257900	-2.71181100
H	-2.00988500	0.01846700	-2.58630600
C	-5.35978000	-0.10264500	-1.98847200
H	-6.27554600	-0.07041800	-0.03439400
H	-4.17921100	-0.11142800	-3.79667000
H	-6.30789900	-0.15753500	-2.51611600
C	-1.43768600	-0.36792500	1.41934900
C	-0.60311600	0.33442300	2.30756900
C	-2.04942900	-1.56676300	1.83492600
C	-0.39443800	-0.15228300	3.59271300
H	-0.14312800	1.26382700	1.99383000
C	-1.81023200	-2.06158200	3.11235100
H	-2.66403700	-2.12666200	1.13832800
C	-0.98661500	-1.35378500	3.99222200
H	0.24272100	0.39772000	4.27827200
H	-2.25608900	-3.00286500	3.41836600
H	-0.80103900	-1.74249400	4.98943200
C	1.18670300	2.17514600	-0.16735800
C	0.73798500	3.17441000	-1.04729500
C	1.85357700	2.58768000	0.99905800
C	0.95683100	4.52754000	-0.78382200
H	0.21058300	2.88698500	-1.95422800
C	2.07089500	3.93830700	1.27489300
H	2.22547400	1.83399000	1.69007300
C	1.62275100	4.91366900	0.38131600
H	0.60811200	5.28059800	-1.48634400
H	2.59498300	4.22937800	2.18191100
H	1.79378400	5.96669300	0.58995000

TS6

SCF Done:-1164.038412

C	-0.43808500	1.15298200	0.62595500
B	0.78068500	0.53087900	-0.79784000
B	-1.48512900	3.45556800	1.78464600
H	-2.12843100	3.96667600	2.64659100
B	-1.94392500	1.86873600	1.10321000
H	-2.84954000	1.17069200	1.42991900
B	-0.49314600	2.00605600	2.11979000
H	-0.41946900	1.40297900	3.14235100
B	0.29460800	3.59060400	1.75912800
H	0.92505000	4.17835800	2.57928000
B	0.91906000	2.11223000	1.10039400
H	1.93827700	1.57069000	1.33747300
C	0.24401900	2.00517400	-0.53428300
B	-0.65743800	4.39514200	0.48802700
H	-0.71411900	5.58235300	0.41690500
B	0.83338700	3.52015900	0.03318000
H	1.83763700	3.96848300	-0.41176400
B	-0.63540800	3.40247900	-0.98960000
H	-0.61524100	3.77116600	-2.11768900
B	-2.06695200	3.36336300	0.10371800
H	-3.12118900	3.78312600	-0.25557100
B	-1.44952300	1.89482200	-0.58072400
H	-1.93761400	1.22583300	-1.41577100
O	-0.10197400	-0.46868700	-1.46691600
C	-0.72071300	-1.12073700	-0.50248700
C	0.07709100	-1.90891600	0.48127200
C	0.92214000	-2.88704700	-0.06262400
C	-0.02277300	-1.76392000	1.87042600
C	1.65334200	-3.72160200	0.78194900
H	1.01609400	-2.97679800	-1.14008700
C	0.71925300	-2.59455800	2.70732100
H	-0.63817500	-0.97100800	2.28065100
C	1.55403300	-3.57742100	2.16589000
H	2.31415000	-4.46976300	0.35436300
H	0.65736900	-2.46235500	3.78348800
H	2.13472100	-4.21866500	2.82285000
C	-2.13070500	-1.41103600	-0.69459900
C	-2.75765900	-1.04412400	-1.90681500

C	-2.88297900	-2.07947600	0.29605300
C	-4.09921700	-1.33303600	-2.11484600
H	-2.17301900	-0.54960400	-2.67232000
C	-4.23222200	-2.33650200	0.09158200
H	-2.40932500	-2.38654800	1.22044700
C	-4.84130600	-1.96552100	-1.11184300
H	-4.57250400	-1.05610600	-3.05173500
H	-4.81014100	-2.83278200	0.86500300
H	-5.89562000	-2.17472100	-1.27019900
C	2.31248700	0.17066100	-0.82302800
C	2.98246500	0.54744700	-2.00092900
C	3.04712400	-0.50945900	0.16084900
C	4.33089600	0.24189400	-2.19761000
H	2.44706200	1.09493100	-2.77459300
C	4.39540200	-0.80994600	-0.02704300
H	2.56408700	-0.79669000	1.08916700
C	5.04115400	-0.43994700	-1.20934000
H	4.82578200	0.54548200	-3.11654900
H	4.94374400	-1.33195400	0.75307600
H	6.09263300	-0.67394000	-1.35469900

4'

SCF Done:-1164.172436

C	-0.57442700	1.15134100	0.00003600
B	1.50353000	-0.24977100	0.00025700
B	-0.78382400	3.90902300	-0.00028100
H	-1.48761700	4.86720800	-0.00032900
B	-1.25865200	2.44634900	-0.89054300
H	-2.25696800	2.27377500	-1.49886800
B	-1.25881400	2.44657400	0.89037100
H	-2.25711700	2.27409400	1.49870900
B	0.13798100	3.41133200	1.45229900
H	0.10170200	3.99970800	2.48400700
B	0.22971500	1.65547800	1.44739800
H	0.27508500	0.87713000	2.33546600
C	1.07694300	1.26599500	0.00023000
B	0.99819400	4.01386200	-0.00011500
H	1.57970500	5.05046300	-0.00008000
B	1.62564100	2.60817300	0.88857100
H	2.64059500	2.50019500	1.49254000
B	1.62578700	2.60800900	-0.88847100
H	2.64092300	2.49967900	-1.49207100
B	0.13827200	3.41097000	-1.45266200
H	0.10219500	3.99917300	-2.48447200
B	0.23005100	1.65520700	-1.44732800
H	0.27498100	0.87628800	-2.33489600
O	0.38795100	-1.03245800	0.00006100
C	-0.90727700	-0.39920300	-0.00001800
C	-1.64475000	-0.87010800	1.26724200
C	-1.06933500	-1.85214600	2.08281200
C	-2.90628700	-0.36945200	1.61462900
C	-1.73432100	-2.31316900	3.22011100
H	-0.09843500	-2.25848300	1.82955500
C	-3.56825800	-0.82757700	2.75227000
H	-3.38082700	0.39322900	1.01208300
C	-2.98548200	-1.80296000	3.56220300
H	-1.26643000	-3.07440000	3.83848200
H	-4.54176300	-0.41621600	3.00394000
H	-3.50161700	-2.15969900	4.44913100
C	-1.64449100	-0.87041600	-1.26737900
C	-1.06836100	-1.85186400	-2.08315300
C	-2.90625000	-0.37039300	-1.61479200
C	-1.73292800	-2.31299600	-3.22064000
H	-0.09726400	-2.25769300	-1.82982800
C	-3.56780100	-0.82860800	-2.75267000
H	-3.38138200	0.39182000	-1.01214200
C	-2.98435000	-1.80342600	-3.56277200
H	-1.26453300	-3.07380300	-3.83915400
H	-4.54150500	-0.41771800	-3.00434100

H	-3.50012600	-2.16027700	-4.44986200
C	2.90992600	-0.87267000	0.00030600
C	4.07159000	-0.07737300	-0.00046200
C	3.05900800	-2.27448200	0.00117200
C	5.33778400	-0.66078300	-0.00038900
H	3.98180800	1.00452600	-0.00116900
C	4.32141200	-2.86094400	0.00119900
H	2.17021200	-2.90039700	0.00171600
C	5.46256000	-2.05208300	0.00044000
H	6.22534900	-0.03416300	-0.00096600
H	4.42120400	-3.94303300	0.00179500
H	6.44938400	-2.50786700	0.00053400

TS7

SCF Done:-1164.047865

C	1.60784900	-1.21534300	0.68652800
B	0.81840200	0.76826600	-0.51392800
B	2.84232000	-3.50637800	-0.40966800
H	3.33683100	-4.57605400	-0.23272400
B	3.04478300	-2.17146700	0.77132700
H	3.64516700	-2.21911200	1.79725300
B	1.47191500	-2.93771200	0.58981100
H	0.95125800	-3.51724600	1.48928800
B	1.22493200	-3.32269400	-1.14594300
H	0.53871100	-4.23255900	-1.49363100
B	0.50172600	-1.89880400	-0.41228100
H	-0.66754300	-1.73812300	-0.26693800
C	1.50187900	-0.59246400	-0.85276800
B	2.67698100	-2.77614300	-2.04216500
H	3.04971800	-3.32654600	-3.03143600
B	1.20662800	-1.77734300	-2.04899200
H	0.49632600	-1.50063400	-2.96496100
B	2.80876600	-1.00991800	-1.86754700
H	3.19122100	-0.21148700	-2.66065200
B	3.81343300	-2.07845000	-0.84997400
H	4.99732600	-2.09808400	-0.97825800
B	3.03456000	-0.67084400	-0.12558500
H	3.58245700	0.31502100	0.22561800
O	-0.61196900	0.77973800	-0.52029700
C	-1.61461400	0.30337500	0.14932200
C	-2.84175400	0.16160800	-0.63268600
C	-4.10801600	0.41979200	-0.07430200
C	-2.73680100	-0.20516000	-1.98965800
C	-5.25029500	0.30175300	-0.86073600
H	-4.18611600	0.74438400	0.95796300
C	-3.88450300	-0.33993700	-2.76078600
H	-1.75817700	-0.41900200	-2.40729500
C	-5.14073800	-0.08461600	-2.19933400
H	-6.22457200	0.51654000	-0.43228700
H	-3.80312700	-0.64789000	-3.79869200
H	-6.03512600	-0.18467600	-2.80777200
C	-1.49073900	-0.00717700	1.54828000
C	-0.39800700	0.49023000	2.29835600
C	-2.37991500	-0.93237500	2.15738000
C	-0.24273400	0.12242700	3.63389000
H	0.25950900	1.23522400	1.87275400
C	-2.19680800	-1.30997700	3.47424400
H	-3.16644000	-1.38948300	1.56856800
C	-1.13088200	-0.77482400	4.21815900
H	0.58704900	0.52527600	4.20519500
H	-2.86591100	-2.03507500	3.92667400
H	-0.99430100	-1.07418500	5.25347100
C	1.38725000	2.21025400	-0.33507900
C	0.84577700	3.21656400	-1.16186400
C	2.33746700	2.59251800	0.62939800
C	1.27231600	4.54008300	-1.06342400
H	0.08976900	2.95745200	-1.89925400
C	2.74387100	3.92125200	0.74869200
H	2.74738300	1.84695200	1.30084300

C	2.22294300	4.89581700	-0.10482400
H	0.85688200	5.29359300	-1.72723600
H	3.47276800	4.19417800	1.50695300
H	2.55118700	5.92827600	-0.01907000

4"

SCF Done:-1164.130217

C	1.34826000	-1.44711900	0.21663200
B	0.46444700	1.16833600	0.18182800
B	3.43534300	-2.36113700	-1.36405200
H	4.12537200	-3.26947900	-1.69936600
B	2.88047800	-2.21299200	0.31357000
H	3.08026300	-2.96853000	1.20184200
B	1.71366900	-2.61962900	-0.96668400
H	1.11341800	-3.64016900	-0.92482600
B	2.16196900	-1.68924600	-2.42663200
H	1.92878100	-2.10116200	-3.51695500
B	0.84370900	-1.14306800	-1.41572900
H	-0.30375700	-1.10745100	-1.67529100
C	1.50132700	0.09242900	-0.38721000
B	3.60721600	-0.71446800	-2.03001600
H	4.41834300	-0.42472000	-2.84943100
B	1.99523100	0.04823400	-2.03635000
H	1.58082200	0.89829600	-2.75314800
B	3.15663400	0.43720900	-0.76226500
H	3.54139100	1.54829500	-0.64618700
B	4.04701100	-1.03300000	-0.32719200
H	5.15891900	-0.98419400	0.09053200
B	2.71065500	-0.48358700	0.68709900
H	2.70693600	-0.05585500	1.79267200
O	-0.84184500	0.84080200	0.42864300
C	-1.62303100	-0.29072400	0.35593000
C	-2.96214000	0.02175700	-0.17718500
C	-4.13165800	-0.51096400	0.38990500
C	-3.07804900	0.90748100	-1.26346400
C	-5.38216000	-0.18817300	-0.13526600
H	-4.05719300	-1.15670800	1.25893800
C	-4.32819200	1.22603700	-1.78690600
H	-2.17999700	1.33328500	-1.70032900
C	-5.48459500	0.67661500	-1.22657100
H	-6.27819400	-0.60276300	0.31823800
H	-4.40089000	1.90271300	-2.63381400
H	-6.46007900	0.92828000	-1.63336500
C	-1.15866200	-1.50366300	0.76171300
C	0.25328300	-1.60833700	1.32437500
C	-1.91296400	-2.73022600	0.58226100
C	0.47720400	-2.87346400	2.11929400
H	0.40907800	-0.75746700	2.00127900
C	-1.51747400	-3.88940400	1.15848100
H	-2.79579300	-2.70587600	-0.04718500
C	-0.34615000	-3.92927300	2.01916900
H	1.35222100	-2.90051200	2.75919700
H	-2.08341500	-4.80162300	0.99480500
H	-0.14318800	-4.83387500	2.58633400
C	0.78728000	2.66524900	0.45977700
C	-0.23238200	3.61532700	0.23702100
C	2.01075200	3.12906600	0.97988900
C	-0.02668600	4.97019500	0.48412200
H	-1.19719200	3.27834800	-0.13203600
C	2.21223900	4.48147600	1.25355200
H	2.80695000	2.42722800	1.19899300
C	1.19898100	5.40632900	0.99431200
H	-0.82174600	5.68506000	0.28915300
H	3.16053700	4.81341600	1.66744000
H	1.36026800	6.46208400	1.19649500

References

[1] Grigsby, W.; Power, P., *J. Am. Chem. Soc.* **1996**, *118*, 7981–7988.

[2] Ren, S.; Xie, Z., *Organometallics* **2008**, *27*, 5167–5168.

[3] Sheldrick, G. *Acta Cryst.* **2015**, *A71*, 3–8.

[4] Sheldrick, G. *Acta Cryst.* **2008**, *A64*, 112–122.

[5] Frisch, M. J.; Trucks, G. W.; Schlegel, H. B.; Scuseria, G. E.; Robb, M. A.; Cheeseman, J. R.;Scalmani, G.; Barone, V.; Mennucci, B.; Petersson, G. A.; Nakatsuji, H.; Caricato, M.; Li, X.;Hratchian, H. P.; Izmaylov, A. F.; Bloino, J.; Zheng, G.; Sonnenberg, J. L.; Hada, M.; Ehara, M.;Toyota, K.; Fukuda, R.; Hasegawa, J.; Ishida, M.; Nakajima, T.; Honda, Y.; Kitao, O.; Nakai, H.;Vreven, T.; Montgomery, Jr., J. A.; Peralta, J. E.; Ogliaro, F.; Bearpark, M.; Heyd, J. J.; Brothers, E.;Kudin, K. N.; Staroverov, V. N.; Kobayashi, R.; Normand, J.; Raghavachari, K.; Rendell, A.; Burant,J. C.; Iyengar, S. S.; Tomasi, J.; Cossi, M.; Rega, N.; Millam, J. M.; Klene, M.; Knox, J. E.; Cross, J.B.; Bakken, V.; Adamo, C.; Jaramillo, J.; Gomperts, R.; Stratmann, R. E.; Yazyev, O.; Austin, A. J.;Cammi, R.; Pomelli, C.; Ochterski, J. W.; Martin, R. L.; Morokuma, .; Zakrzewski, V. G.; Voth, G.A.; Salvador, P.; Dannenberg, J. J.; Dapprich, S.; Daniels, A. D.; Farkas, Ö.; Foresman, J. B.; Ortiz,J. V.; Cioslowski, J.; Fox, D. J. Gaussian 09, Revision D.01; Gaussian, Inc.: Wallingford, CT, **2009**.

[6] a) Becke, A. D. *J. Chem. Phys.* **1993**, *98*, 5648-5652; b) Lee, C.; Yang, W.; Parr, R. G. *Phys. Rev. B: Condens. Matter* **1998**, *37*, 785-789; c) Stephens, P. J.; Devlin, F. J.; Chabalowski, C. F.; Frisch, M. J. S17 *J. Phys. Chem.* **1994**, *98*, 11623-11627; d) Vosko, S. H.; Wilk, L.; Nusair, M. Can. J. Chem. 1980, 58, 1200-1211. e) P. C. Hariharan, J. A. Pople, Theor. Chim. Acta 1973, 28, 213–222; f) V. A. Rassolov, M. A. Ratner, J. A. Pople, P. C. Redfern, L. A. Curtiss, J. Comput. Chem. 2001, 22, 976–984; g) W. J. Hehre, R. Ditchfield, J. A. Pople, J. Chem. Phys. 1972, 56, 2257–2261; h) M. M. Francl, W. J. Pietro, W. J. Hehre, J. S. Binkley, M. S. Gordon, D. J. DeFrees, J. A. Pople, J. Chem. Phys. 1982, 77, 3654–3665; i) R. Ditchfield, W. J. Hehre, J. A. Pople, J. Chem. Phys. 1971, 54, 724–728; j) J. D. Dill, J. A. Pople, J. Chem. Phys. 1975, 62, 2921–2923; gM. S. Gordon, J. S. Binkley, J. A. Pople, W. J. Pietro, W. J. Hehre, J. Am. Chem. Soc. 2002, 104, 2797–2803.

[7] a) Fukui, K. *J. Phys. Chem.* **1970**, *74*, 4161–4163; b) Fukui, K. *Acc. Chem. Res.* **1981**, *14*, 363–368.

[8] a) Zhang, J.-X.; Sheong, F. K.; Lin, Z. *Chem. Eur. J.* **2018**, *24*, 9639–9650; b) Zhang, J.-X.; Sheong, F. K.; Lin, Z. *WIREs Comput. Mol. Sci.* **2020**, *10*, e1469.

[9] a) Clark, T.; Chandrasekhar, J.; Spitznagel, G. W.; Schleyer, P. Von R. *J. Comput. Chem.* **1983**, *4*, 294-301; b) Krishnan, R.; Binkley, J. S.; Seeger, R.; Pople, J. A. *J. Chem. Phys.* **1980**, *72*, 650-654.

[10] Wolinski, K.; Hinton, J. F.; Pulay, P. *J. Am. Chem. Soc.* **1990**, *112*, 8251-8260.

[11] Schleyer, P. V.; Maerker, C.; Dransfeld, A.; Jiao, H. J.; Hommes, N. J. R. V. *J. Am. Chem. Soc.* **1996**, *118*, 6317-6318.

[12] a) Schleyer, P. V.; Manoharan, M.; Jiao, H. J.; Stahl, F. *Org. Lett.* **2001**, *3*, 3643-3646; b) Corminboeuf, C.; Heine, T.; Seifert, G.; Schleyer, P. V.; Weber, J. *Phys. Chem. Chem. Phys.* **2004**, *6*, 273-276.

[13] a) Perdew, J. P. *Phys. Rev. B* **1986**, *33*, 8822–8824; b) Becke, A. D. *Phys. Rev. A* **1988**, *38*, 3098–3100; c) Grimme, S.; Ehrlich, S.; Goerigk, L. *J. Comput. Chem.* **2011**, *32*, 1456–1465; d) Johnson, E. R.; Becke, A. D. *J. Chem. Phys.* **2005**, *123*, 24101; e) Grimme, S.; Antony, J.; Ehrlich, S.; Krieg, H. *J. Chem. Phys.* **2010**, *132*, 154104; f) Becke, A. D.; Johnson, E. R. *J. Chem. Phys.* **2005**, *122*, 154104.

[14] a) Böhrer, H.; Trapp, N.; Himmel, D.; Schleep, M.; Krossing, I. *Dalton Trans.* **2015**, *44*, 7489–7499; b) Greb, L. *Chem. Eur. J.* **2018**, *24*, 17881–17896; c) Müller, L. O.; Himmel, D.; Stauffer, J.; Steinfeld, G.; Slattery, J.; Santiso-Quiñones, G.; Brecht, V.; Krossing, I. *Angew. Chem. Int. Ed.* **2008**, *47*, 7659–7663.

Author Contributions

Q.Y. conceived the project. Y.W. and W.Y. carried out all the experiments. J.W. and Z.L. performed the DFT calculations. All authors discussed the results and contributed to the final manuscript.
